# The impact of political ideologies on cultural erosion: Indigenous education policy in Brazil (2009–2022)

**DOI:** 10.1371/journal.pone.0354262

**Published:** 2026-07-23

**Authors:** André Calixto Gonçalves, Rodolfo Valentim, Francisco Aparecido Rodrigues, Guilherme Antônio de Almeida Lopes Fernandes, Ivan Filipe Fernandes

**Affiliations:** 1 CECS - Center for Engineering, Modeling and Applied Social Sciences, Federal University of ABC, São Bernardo do Campo, Brazil; 2 Department of Physics, Federal University of São Paulo, Diadema, Brazil; 3 Instituto de Ciências Matemáticas e de Computação, Universidade de São Paulo, São Carlos, São Paulo, Brazil; 4 Faculty of Law, University of São Paulo, São Paulo, Brazil; 5 School of Engineering and Management, Faculty of Law, ESEG Grupo Etapa, São Paulo, Brazil; 6 Laboratory for Prosocial Behavior and Public Policy - Pensi Institute, São Paulo, Brazil; 7 Centre for Interdisciplinary Studies – CEIS20, University of Coimbra, Coimbra, Portugal; Public Library of Science, UNITED KINGDOM OF GREAT BRITAIN AND NORTHERN IRELAND

## Abstract

Indigenous schools in Brazil aim to provide equitable education to both Indigenous and non-Indigenous communities. As of 2022, there were 3,411 such schools operating nationwide, serving the entire Indigenous population. However, an analysis of educational data reveals significant non-compliance with legislation designed to integrate Indigenous culture into the curriculum. Our research demonstrates that the school system frequently falls short of its legal obligation to promote and safeguard Indigenous cultural values. The systematic suppression of culturally specific materials and native languages is particularly alarming and appears to be associated with the influence of far-right political administrations. This study analyzes school census data from 2009 to 2022, employing chi-square tests, Cramer’s V tests, descriptive statistics and for robustness hierarchical mixed models to examine the relationship between political ideology and educational practices.

## Introduction

Political ideology plays a central role in shaping public policy in Brazil [1]. Elected officials often frame education and cultural policies according to their ideological orientations. For example, left-wing leaders tend to emphasize multiculturalism and minority rights, whereas conservative or far-right leaders typically prioritize national cohesion and cultural assimilation [2]. This ideological influence is particularly pronounced in Indigenous affairs, despite the 1988 Federal Constitution’s explicit commitment to preserving Indigenous languages and cultures—most relevant through Articles 231–232 on Indigenous rights and Article 210 on education. However, the way the constitutional commitment is implemented has varied significantly across different administrations. Here, we show political ideology shapes policy approaches to Indigenous schooling [3].

Despite Brazil’s highly fragmented party system—with dozens of parties spanning the ideological spectrum—ideological orientation remains a meaningful predictor of policy choices [4]. Fragmentation has not erased substantive ideological differences [5]. The Workers’ Party (PT) dominates the center-left, alongside smaller allies such as the Socialism and Freedom Party (PSOL) and the Brazilian Socialist Party (PSB). On the right, however, a multitude of parties exists, ranging from neoliberal fiscal conservatives to fundamentalist nationalists. Although these right-leaning parties diverge on economic issues, they tend to converge on socially conservative agendas.

Even as Brazil’s party system has become more fragmented, ideological cleavages have persisted, and in some cases, ideological differences actually narrowed prior to the rise of the far right [6]. In other words, the divide between left and right-wing values continues to predict policy behavior on many issues. For instance, mayors affiliated with the Workers’ Party and other leftist parties have historically played an active role in advancing reforms in health and education. Left and center-left administrations are generally more inclined to implement redistributive social policies [7]. Further studies demonstrate that a mayor’s partisan affiliation significantly influences local budgetary decisions and public spending priorities [8]. Building on this body of evidence, we argue that party affiliation serves as a reliable proxy for identifying ideological orientation [9].

This ideological dynamic is especially consequential for Indigenous rights and education. Following the 1988 Constitution, Brazil shifted from a model of coercive assimilation toward one that recognizes Indigenous autonomy and culture. State and municipal education policies were intended to provide classroom instruction in Indigenous languages and incorporate cultural materials, consistent with constitutional guarantees of bilingual and intercultural education [10].

Indigenous peoples have the right to specific, intercultural, bilingual/multilingual, community-based, and differentiated school education, according to Brazilian legislation, which underpins Indigenous School Education. The concept of bilingual/multilingual education involves reaffirming each indigenous people’s identity in contrast to the historical imposition of a colonial education model that sought to suppress local customs and languages, deeming them inferior, pagan, or simply uncivilized. In this sense, the education values indigenous languages and provides a bridge to Portuguese, as a broader language, if that is the wish of each community, in accordance with the collaborative regime established by the Brazilian Federal Constitution of 1988 and the National Education Guidelines Law (LDB). It is worth noting that the national coordination of Indigenous School Education policies is under the Ministry of Education’s responsibility. However, it is the role of the states and municipalities within the Brazilian federation to implement measures to guarantee these rights for indigenous peoples [11–13].

In practice the degree of implementation of those policies has fluctuated with political shifts. Since the 1990s, left-wing governments have often supported the training of Indigenous teachers, the inclusion of native languages in school curriculum, and the funding of Indigenous cultural projects. Conversely, center-right administrations have at times curtailed support for these initiatives, citing budgetary constraints or appeals to “national unity.” The far-right Bolsonaro administration (2019–2022) represented a particularly stark departure: it openly challenged Indigenous rights, proposing, for example, that FUNAI (the National Indian Foundation) be subordinated to the Ministry of Agriculture, while adopting an adversarial stance toward Indigenous land claims [14].

Hence. the main objective of this study is to quantitatively analyze how political ideology influences Indigenous education in Brazil between 2009 and 2022. Specifically, it investigates whether the ideological orientation of state and municipal governments—ranging from left to far-right—affects the implementation of culturally responsive education in Indigenous schools. To do so, the study examines four key indicators: the use of Indigenous, Portuguese or both Indigenous and Portuguese in classroom instruction and the use of teaching materials based on Indigenous cultural content.

Brazil represents an interesting case to investigate the relation between indigenous education policy and ideology as the Brazilian education system operates under a multi-level federal arrangement in which subnational authorities retain substantial decision-making competence over curriculum design, continuing education, material adoption, and language policies [15]. The system is formally decentralized, yet highly permeable to partisan competition, coalition bargaining, and judicial intervention—particularly in domains involving minority rights, where courts, prosecutors, and constitutional oversight agencies frequently intervene [16]. The institutional configuration interacts with an unusually large and diverse Indigenous population, producing strong regional heterogeneity. Therefore, the Brazilian case combines high multi-level institutional complexity, a historically mobilized Indigenous movement, and systematic nationwide microdata on schools. Together, these features provide a rare opportunity to empirically observe how subnational partisan ideology translates into concrete policy outcomes within the same constitutional framework—making Brazil adequate for studying political drivers of cultural erosion in Indigenous education.

While several studies examine Indigenous rights, state capacity, and partisan dynamics in Brazil and other democracies, the literature has not yet empirically demonstrated whether the ideological orientation of subnational authorities translates into measurable patterns of cultural erosion within Indigenous education systems over time. Even in countries where Indigenous schooling is a longstanding policy field — such as Canada, Australia, and New Zealand — most analyses emphasize historical trauma, systemic exclusion, and unequal outcomes, but do not test partisan ideological effects longitudinally at the subnational level [17–19]. Our study directly addresses it by linking partisan ideology to concrete indicators of Indigenous cultural protection in the largest Indigenous school system in Latin America. By doing so, the study shows how cultural erosion can operate through formal legal change and routine administrative decisions over curriculum, language, and materials.

Since the colonial period, schooling in Brazil has been used as an instrument of cultural assimilation rather than intercultural dialogue. The Jesuit missions of the sixteenth century aimed to replace Indigenous cosmologies with Christian and European norms, a pattern later reinforced under the military dictatorship through large-scale development projects that displaced Indigenous populations. Only after the 1988 Federal Constitution did the country formally recognize the right to bilingual and intercultural education, reaffirmed by the National Education Guidelines Law (LDB, 1996) and subsequent policy frameworks. Yet, despite this robust legal foundation, implementation has remained inconsistent, with persistent suppression of Indigenous languages and cultural content in schools. This enduring gap between legislation and practice highlights the importance of analyzing how political ideology affects the protection or erosion of Indigenous education and culture across different administrations (Carneiro, 2009; Gallois et al., 2020) [20,21].

Using data from the Brazilian School Census spanning 2009–2022, we identify all officially recognized Indigenous schools. Our findings indicate that ideological shifts have tangible effects: many local governments controlled by right-leaning coalitions have reduced bilingual offerings and cultural content in Indigenous schools, while left-leaning governments have tended to expand or at least safeguard these programs. In short, right-wing and especially far-right governments in Brazil have been more likely to erode Indigenous educational protections, whereas left-wing administrations have largely defended or strengthened them.

Our methodological approach combines descriptive analysis with statistical testing. We first present descriptive trends, illustrating how rates of Indigenous language instruction and the inclusion of cultural materials have changed between 2009 and 2022 across different regions. We categorize observations by the ideological orientation of governing administrations, distinguishing between left, center-left, center, center-right, right and far-right leadership. To assess the association between ideology and educational indicators, we employ Pearson’s chi-square tests of independence and Cramer’s V tests, applying these procedures to multiple indicators at both the state and municipal levels. For robustness, we employed hierarchical mixed models weighted by the number of schools per municipality or state-year, giving greater influence to estimates derived from larger educational networks and accounting for heteroscedasticity in proportional outcomes.

Thus, our analytical strategy operationalizes “ideological spectra” as categorical distinctions among governments, while leveraging school census data to capture educational practices. In sum, the research investigates whether the provision of culturally responsive education in Indigenous schools correlates with political ideology over time. A detailed account of our empirical strategy is provided in the Data and Methods section.

This study focuses on the period 2009–2022 because it corresponds to the complete and standardized availability of school census microdata on Indigenous education provided by INEP. This period also encompasses major political transitions in Brazil — from left to far-right administrations — allowing for a comprehensive assessment of how ideological shifts affected Indigenous education policies. The analysis is restricted to schools officially located within recognized Indigenous lands, as defined by FUNAI and the Ministry of Education, to ensure that the institutions analyzed are genuinely Indigenous in nature and subject to specific legal protections under Brazilian law. Finally, the study employs binary variables (coded 1 for presence and 0 for absence) to capture whether language instruction and culturally specific teaching materials are present in each school year. This operationalization follows the structure of INEP’s dataset, which records such features categorically rather than continuously.

The remainder of the paper is organized as follows. First, we offer a contextual background and review the literature on Brazilian politics and Indigenous education. Next, we describe the data and methodological approach in detail, explaining how we classified ideological orientations and which census indicators we analyzed. We then present the results of our descriptive, chi-square, Cramer’s V and for robustness tests a hierarchical mixed models analyses, emphasizing key differences across ideological groups over time. Finally, we discuss the findings and their broader implications for understanding how political ideologies influence cultural preservation and education policy in Brazil.

### Overview of indigenous characteristics and cultural suppression in Brazil

Brazil has a population of approximately 210 million people, including about 1.7 million Indigenous individuals belonging to 305 distinct ethnic groups. Collectively, these groups speak 274 languages, making Brazil one of the most socio-culturally diverse countries in the Americas [22–27]. This demographic and linguistic plurality forms the foundation for understanding the historical and contemporary challenges faced by Indigenous peoples.

The descendants of Indigenous peoples frequently face significant barriers to accessing the essential resources necessary for their survival and well-being. Despite these precarious conditions, Brazil has made efforts to develop an educational infrastructure that is responsive to the needs and cultural specificities of Indigenous peoples. Within this broader context of inequality and policy efforts, a rich intellectual and cultural production has emerged, offering nuanced reflections on Indigenous identities, worldviews, and political struggles [20].

According to the most recent data (2022), there are 3,411 Indigenous schools across the country. Of these, 66.11% are located in the North region, 19.23% in the Northeast, 7.86% in the Midwest, 4.69% in the South, and 2.11% in the Southeast [22,26–28]. The geographic distribution of these schools reflects both the concentration of Indigenous populations and the regional variations in historical settlement, linguistic diversity, and territorial rights.

**Fig 1:**
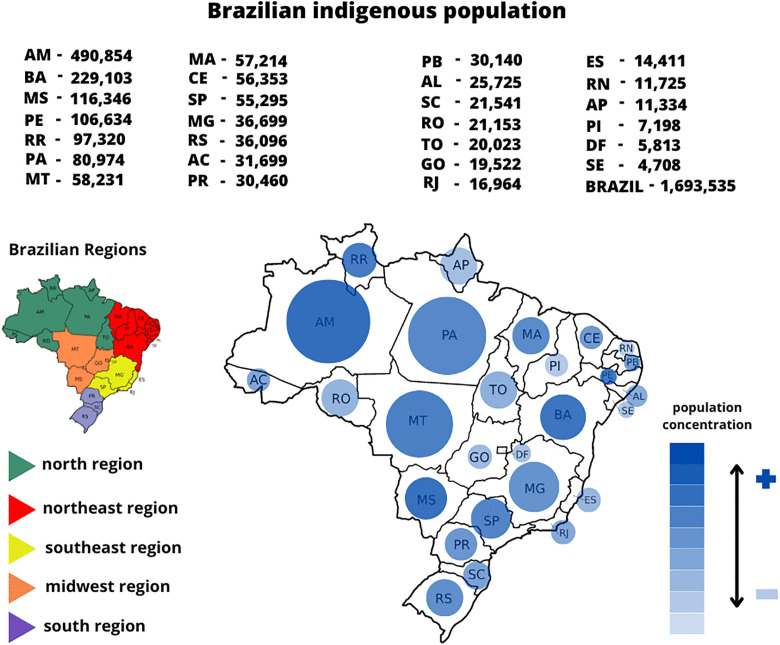
Distribution of Brazil’s indigenous population according to self-identification. Source: IBGE – Demographic Census – 2022.

Brazilian Indigenous communities are often categorized along linguistic lines, with the Tupi and Macro-Jê families representing the two most prominent groupings. Shared linguistic and cultural lineages enable these groups to sustain and transmit their communal traditions, including myths, kinship systems, family structures, festivals, belief systems, dietary customs, agricultural practices, and artisanal crafts. At the same time, each group actively strives to preserve its distinct identity and to assert its legitimate claims over ancestral territories [22,26–30]. Taken together, these elements illustrate how language, education, territoriality, and cultural production are deeply interconnected facets of Indigenous life in Brazil, a pattern that is spatially reflected in the distribution of the Indigenous population across the country (Fig 1).

As illustrated in Fig 1, the significant size and broad geographic distribution of Brazil’s Indigenous population across all states presents considerable challenges for meeting their educational needs, both in terms of scale and complexity. These challenges are further compounded by the country’s historical legacy of extractivism, which has shaped Brazil’s development since colonization. This legacy initially manifested in an Indigenous education model rooted in the Jesuit system established in the 16th century [31,32].

The Jesuit model sought to integrate Indigenous peoples into a Eurocentric worldview aligned with the Catholic Church’s Counter-Reformation agenda. Education functioned as a tool for religious conversion and cultural assimilation, systematically suppressing native cosmologies while imposing Christian doctrines and European social norms. Based on principles of catechization, “civilization,” and coerced assimilation into colonial society, this model promoted European values while undermining Indigenous identities and erasing their unique cultural heritage [31,32].

During the military dictatorship (1964–1985), these coercive assimilation efforts intensified through large-scale infrastructure projects targeting sparsely populated regions of the interior. Notable among these were the construction of the Transamazon Highway in 1972, the planning of the Belo Monte and Itaipu hydroelectric plants in 1975, and the completion of the Perimetral Norte Highway in 1973 [33,34]. These projects caused a deep transformation in the social and cultural structure of Indigenous communities, often dismantling their traditional ways of life. Tragically, these developments also resulted in widespread victimization and the deaths of Indigenous Brazilians at the hands of economic and agrarian elites [33,34].

Within this context, the historical narrative of decimation and suppression experienced by Indigenous communities left deep-seated traumas embedded in educational structures and practices. This somber legacy—spanning from the colonial period through the 20th century—reflects the systematic imposition of a dominant educational model that disregarded and actively suppressed ancestral knowledge and cultural traditions [31,32].

Following the promulgation of the 1988 Federal Constitution, coercive assimilation policies began to give way to more inclusive approaches as civil society organizations mobilized to advance Indigenous rights, fostering broader dialogue within and across Indigenous communities. Since then, the establishment of educational institutions specifically designed for Indigenous populations has undergone a significant transformation [22,26,35,36].

These institutions now play a crucial role in ensuring equitable access to education while simultaneously preserving Indigenous cultural identities and values. They serve as vital instruments for safeguarding Indigenous rights, fostering pride in cultural heritage, and promoting more harmonious engagement with non-Indigenous society [22,26,35,36].

To support the advancement of Indigenous educational institutions, a set of political and legislative measures have been enacted, including Law 9.394/1996, Law 13.005/2014, CNE/CEB Technical Opinions No. 13 and 14, and CEB Resolution No. 3. Importantly, the 1988 Constitution granted formal recognition to Indigenous peoples and their rights, embracing their social structures, ancestral customs, distinct languages, spiritual beliefs, and inalienable territorial claims [36,37].

Culturally relevant teaching materials in the context of Indigenous peoples refers to those produced with the active participation of Indigenous communities. In this sense, they are materials that incorporate the so-called “traditional knowledge”; that is, language, folklore, and the entire cosmology that surrounds each people, utilizing local materials and resources. Through these, it is possible to maintain cultural identity across generations while also fostering dialogue and cultural exchange, both inherent and external to Indigenous culture. Among the local materials and resources, we can highlight the use of clay, seeds, feathers, plant fibers, and wood, which strengthen connections to nature and the environment. All of these can be integrated with books, games, musical instruments, and other objects that aid in learning and the transmission of knowledge. It is important to emphasize that the use of these educational materials is fundamental to effective teaching, as they are embedded in the life context of Indigenous students, facilitating understanding and the learning process.

Today, Indigenous schools seek to integrate contemporary pedagogical practices—which value Western scientific knowledge—with Indigenous education, which is grounded in traditional wisdom and communal knowledge. Through consultation with Indigenous communities and engagement with national representative bodies such as the Articulation of Indigenous Peoples of Brazil (APIB), it is possible to ensure that educational practices protect Indigenous knowledge and properly respect and recognize their cultural values [22,26].

Despite the constitutional protections and regulatory frameworks in place, efforts to assimilate Indigenous communities into mainstream Brazilian society persist [38–40]. These assimilationist policies trace their origins to the military dictatorship, a period marked by egregious violations of Indigenous rights and well-being. During this era, deliberate efforts were made to impose territorial assimilation on Indigenous groups, particularly in the Amazon and Midwest regions, resulting in disproportionately high rates of infant mortality and malnutrition among Indigenous populations compared to the general population. The enduring disparities between Indigenous peoples and the broader society remain a legacy of this historical neglect, highlighting the ongoing failure to fully realize the constitutional principles of Indigenous protection and autonomy [33,41].

Today, the extractive paradigm still continues to marginalize Indigenous communities, particularly through sectors such as mining, agribusiness, illegal logging, and civil construction, all of which show little regard for environmental sustainability [42,43]. This model exacerbates ecological degradation and places at risk those communities whose survival depends on harmonious coexistence with nature. [44–46].

The complex dynamics of Indigenous assimilation in Brazil are closely linked to the competing political projects that have emerged over the past fifteen years. Following a prolonged period of political crisis, Brazil’s political forces have largely split into two camps: one committed to upholding the rights enshrined in the 1988 Constitution, and another seeking to revive initiatives reminiscent of the military regime, reintroducing sociopolitical practices from the 1970s [47–49]. This polarization, combined with the COVID-19 pandemic and the relaxation of environmental regulations and protective institutions from 2019 onward, has led to a resurgence of deforestation, land exploitation, agribusiness expansion, and unregulated mining—reaching levels comparable to those seen during the military regime [44–52,53–55].

Amid this polarized context, our research examines how political divisions affect the preservation of Indigenous culture, particularly through education. By analyzing the use of culturally relevant teaching materials and languages of instruction in schools, we distinguish between democratic educational models that protect Indigenous identity and authoritarian assimilationist approaches. The presence or absence of culturally appropriate materials and native language instruction serves as a key indicator of these contrasting models within Brazil’s public education system. Given this focus on how political orientations shape educational practices, it becomes essential to understand the institutional channels through which ideology can influence what is taught in Indigenous schools.

Brazilian education governance reinforces the expectation that political ideology at the subnational level will affect Indigenous educational content more closely. Implementing curriculum decisions, material adoption, and continuing education policies is largely driven by subnational authorities, especially state governments. The states, acting through their respective Secretariats and State Education Councils, operate as regulatory instances with normative power, determining the effective scope of the curriculum [56]. Research shows the Brazilian federative system produces differentiated policy trajectories across subnational units because councils operate under variable partisan coalitions, asymmetric state capacity, and uneven bureaucratic insulation [57]. Thus, while councils exist as formal participatory and collegial arenas, their decisions are highly conditioned by the political orientation of executive leadership, especially in contexts involving minority rights. This institutional arrangement provides a link between subnational leaders’ ideology and Indigenous educational content.

## Methods and data

We conducted a comprehensive analysis using the School Census Microdata from 2009 to 2022. This rich dataset provides detailed information on Brazilian educational institutions, including Indigenous schools, covering aspects such as enrollment, services, pedagogical resources, faculty, and regional context. The microdata, produced by the Anísio Teixeira Institute for Educational Studies and Research (INEP), include records from exams, surveys, and evaluations.

INEP gathers educational data primarily through the annual School Census, conducted jointly by federal, state, and municipal governments. All public and private institutions participate, providing information on:

(i) regular education (early childhood, elementary, and secondary),
(ii) special education (for students with specific needs),
(iii) youth and adult education, and
(iv) vocational education (technical and continuing training programs).

The Census operates in two phases: the first collects data on institutions, classes, students, and teachers; the second captures student progress and performance [58].

Population data were sourced from the 2022 Demographic Census by the Brazilian Institute of Geography and Statistics (IBGE). Political information was obtained from the Superior Electoral Court (TSE) and transparency portals, allowing us to classify governors and mayors by their party ideologies [27,59].

Our study focuses exclusively on Indigenous schools located within officially recognized Indigenous lands. This process yielded a comprehensive dataset of all Indigenous schools recorded between 2009 and 2022.

Each identified school was then matched to its municipality and state, enabling us to document the political leadership—mayors and governors—and their respective party affiliations for each year. For every school year and location, we recorded indicators such as whether schools offered Indigenous language classes, Portuguese language classes or both languages at the same time and the use of nationally recommended Indigenous materials. By linking these data to political ideology, we could assess, for instance, whether Indigenous schools in left-leaning states were more likely to teach native languages than those in right-leaning states.

To track changes over time, we analyzed four key binary variables: Indigenous Material (use of culturally specific educational materials), use of Indigenous Language, Portuguese Language or both Indigenous and Portuguese to instruction in school classes, coded as 1 for presence and 0 for absence. These indicators allowed us to assess the incorporation of Indigenous cultural content and language in classrooms from 2009 to 2022 and to examine their association with the ideological orientation of state and municipal governments.

To ensure data reliability, we screened for multivariate outliers, as outliers can bias results but may also reveal important patterns. Thus, we carefully evaluated each case, retaining only those that provided meaningful insights [60]. The initial 2009 School Census dataset comprised 255,445 educational institutions nationwide, while in 2022 this number had decreased to 224,649. After applying our filters to focus exclusively on Indigenous schools located within Indigenous lands, the dataset was reduced to 2,441 institutions in 2009 and 3,411 in 2022. This represents a 39.8% increase in the number of Indigenous schools over the period, in contrast to a 12.1% decrease in the total number of educational institutions nationwide, which suggests a growing emphasis on Indigenous education during the period under analysis.

To identify the mayors and governors in office each year, we analyzed data from the Superior Electoral Court (TSE), focusing on election winners from 2008 to 2022. This step was essential given the frequent interruptions in political terms due to impeachment, death, disqualification, or other unforeseen events. By tracking actual officeholders annually, we ensured more accurate alignment between political leadership and educational policy outcomes.

Recognizing the limitations of electoral data—particularly leadership changes within terms—we also consulted official transparency portals of municipalities and states. These sources allowed us to confirm who held office each year. For analytical consistency, we considered the individual who governed for the majority of the year as the incumbent. This approach provided a more precise picture of political leadership, ensuring that our analysis reflected those who most directly influenced Indigenous education policies over time.

To determine political ideology, we classified each officeholder’s party affiliation using the framework developed by Bolognesi et al. (2022). Although Brazil has 31 registered parties, prior research [41,44] shows that party statutes often serve as formalities rather than true indicators of ideological commitments [45,46]. Bolognesi et al.’s classification offers a nuanced, dynamic mapping of party ideologies, accounting for frequent shifts due to internal restructuring, party mergers, and broader political changes. Their 2022 survey of 519 political scientists from the Brazilian Association of Political Science (ABCP) produced the ideological classification presented in Table 1, which we used consistently to track political orientation across all government levels throughout the study period [61].

**Table 1:** Ideological spectrum of political parties in Brazil.

Name	Old Acronym	Current Acronym	Political Spectrum
Democrats	DEM	Brazil Union	Far Right
Christian Social Democratic Party	PSDC	DC	Far Right
National Ecological Party	PEN	Patriot	Far Right
Liberal Party	PL	PL	Far Right
Progressives	PP	PP	Far Right
Progressive Republican Party	PRP	Patriot	Far Right
Christian Social Party	PSC	PSC	Far Right
Social Liberal Party	PSL	Brazil Union	Far Right
NOVO	NOVO	NOVO	Far Right
Brazil Union	Brazil Union	Brazil Union	Far Right
Brazilian Democratic Movement Party	PMDB	MDB	Right
Free Fatherland Party	PPL	PC do B	Right
Party of the Republic	PR	PL	Right
Brazilian Republican Party	PRB	Republicans	Right
Republican Party of Social Order	PROS	SD	Right
Brazilian Renewal Labor Party	PRTB	PRTB	Right
Social Democratic Party	PSD	PSD	Right
Brazilian Social Democracy Party	PSDB	PSDB	Right
Christian Labor Party	PTC	Agir	Right
National Labor Party	PTN	Podemos	Right
Republicans	Republicans	Republicans	Right
Podemos	Podemos	Podemos	Right
Humanist Solidarity Party	PHS	Podemos	Center Right
Brazilian Women’s Party	PMB	PMB	Center Right
National Mobilization Party	PMN	Mobiliza	Center Right
Labor Party of Brazil	PT do B	Avante	Center Right
Brazilian Labor Party	PTB	PTB	Center Right
Solidarity	SD	SD	Center Right
Socialist People’s Party	PPS	Cidadania	Center
Green Party	PV	PV	Center
REDE	REDE	REDE	Center
Democratic Labor Party	PDT	PDT	Center Left
Brazilian Socialist Party	PSB	PSB	Center Left
Communist Party of Brazil	PC do B	PC do B	Left
Workers’ Party	PT	PT	Left
Party of Socialism and Freedom	PSOL	PSOL	Far Left

Source: Data produced from the Bolognesi et. al, 2022 [45] survey.

The relationship between partisanship and ideology in Brazil is not perfectly coherent [62]. Partisan identification and political ideology tend to be less tightly aligned outside the OECD, where parties are often personalistic and lack programmatic cohesion [63–65]. Brazil occupies an intermediate position on this global spectrum: while some national parties maintain consistent ideological orientations, others display internal heterogeneity and shifting coalitions [66]. Mayors, in particular, frequently change parties for strategic or pragmatic reasons [67,68]; although such realignments usually occur among ideologically proximate parties rather than across the left–right divide [69,70].

Despite these discontinuities, research consistently finds that mayors’ and governors’ partisan affiliations correspond to recognizable tendencies that shape policy outcomes [71–76]. Based on this accumulated evidence, our analysis assumes that party identification remains a valid and empirically grounded proxy for the ideological orientation of subnational executives.

Table 1 shows that most political parties in Brazil are positioned on the right of the ideological spectrum. However, a greater number of right-wing parties does not necessarily imply stronger political representation. The Brazilian right is characterized by fragmentation, with frequent party-switching among politicians, whereas the left is more consolidated, primarily around one major party (the Workers’ Party, PT) and two smaller but influential ones (PSB and PSOL). As Bolognesi et al. (2022) emphasize, party-switching is a recurrent phenomenon within Brazil’s conservative political field.

To assess the influence of political ideology on Indigenous education, we conducted an empirical analysis using R software, applying Chi-square, Cramer’s V and for robustness tests a hierarchical mixed models to examine associations between political orientation and educational indicators: the use of Indigenous teaching materials and the use of Indigenous languages, Portuguese language or both in classroom instruction.

As Cohen (1988) emphasizes, effect size measures such as Cramer’s V are essential for complementing significance tests by providing insight into the practical relevance of associations. While the chi-square test effectively identifies whether a statistically significant relationship exists between categorical variables, it does not convey the strength of that association [77].

Cramer’s V is particularly suitable for our analysis, as it offers a standardized measure of effect size for nominal data, ranging from 0 (no association) to 1 (perfect association). This enables us to determine whether significant results reflect meaningful relationships or are simply artifacts of large sample sizes.

Given the categorical structure of our variables and the non-directional nature of our hypotheses, Cramer’s V is the most appropriate statistic for quantifying the association between political ideology and the presence of Indigenous educational materials and language instruction.

By reporting both chi-square significance levels and Cramér’s V values, we provide a more nuanced interpretation of our findings, balancing statistical significance with practical significance.

It is generally advisable to calculate and report Cramer’s V only for those relationships where the chi-square test indicates statistical significance. This is because Cramer’s V quantifies the strength of an association that has already been established as statistically significant. If the chi-square result is not significant (p > 0.05), it suggests that any observed association is likely due to random chance, making the interpretation of effect size less meaningful or even misleading. Therefore, in our analysis, we compute Cramer’s V only for those cases where the chi-square test yielded statistically significant results.

### Robustness test

To ensure the robustness of our findings and to account for potential hierarchical dependencies in the data, we conducted an additional analysis using hierarchical mixed-effects models. This approach allows for the inclusion of both fixed effects—representing ideological orientations of governors—and random effects that capture unobserved heterogeneity across states. Because educational outcomes are not independent across municipalities within the same state, mixed models are particularly suitable for testing whether the observed ideological effects persist when this multilevel structure is taken into account.

Moreover, as the dependent variables in our study represent proportional outcomes (i.e., the share of schools adopting Indigenous teaching materials or languages), observations based on municipalities with more schools provide more reliable estimates than those derived from smaller school systems. To correct for this imbalance and mitigate heteroscedasticity, the models were weighted by the number of schools per state and municipality-year, giving greater influence to estimates derived from larger educational networks. This weighting ensures that results reflect both the scale and precision of local educational systems.

The hierarchical models serve as a robustness check by incorporating random intercepts for states and analytical weights (number of schools per municipality-year)**.** We estimated linear mixed-effects models (LMMs) using the lme4 and lmerTest packages in R. The dependent variables corresponded to four main indicators: (1) the percentage of schools using Indigenous pedagogical materials, (2) the percentage of schools teaching exclusively in Indigenous languages, (3) the percentage of schools teaching exclusively in Portuguese language and (4) the percentage adopting both Portuguese and Indigenous languages (bilingual education). The main explanatory variables were the political ideologies of mayors and governors, coded as categorical factors (left, center-left, center, center-right, right, and far-right). Random intercepts were specified for each state to model unobserved contextual differences, while time trends were included through a linear variable representing the census year (2009–2022) [78,79].

Additionally, the results were further complemented by estimated marginal means (EMMs) and pairwise Tukey-adjusted comparisons to evaluate the direction and strength of ideological differences. These tests confirm the ideological effects observed in the main analysis are not artifacts of sample composition or model specification, but remain statistically consistent when hierarchical data structures and heteroscedasticity are appropriately addressed.

## Results

We examine whether political ideology is associated with four indicators of culturally responsive education: the use of Indigenous teaching materials and the use of Indigenous, Portuguese, or both languages in classroom instruction (each coded as 1 for presence and 0 for absence). Ideology was operationalized as a categorical spectrum ranging from left to far-right, allowing us to test whether shifts in political orientation correspond to changes in educational practices within Indigenous schools.

A clear distinction emerges between municipal and state executives. Mayoral ideology shows no statistically significant association with either the use of Indigenous materials (p = 0.159) or language instruction (p = 0.664), suggesting that municipal political orientation does not systematically shape Indigenous educational practices. By contrast, gubernatorial ideology displays consistent and statistically significant associations across all indicators. In state schools, the governor’s political alignment significantly predicts the use of Indigenous materials (p = 0.003) and Indigenous language instruction (p < 0.001). Similar patterns are observed in municipal schools: the governor’s ideology is significantly associated with Indigenous material use (p = 0.045) and with language instruction (p < 0.001). These results indicate that state-level executives shapes the cultural content of Indigenous education in Brazil.

Next, we assess the magnitude of these relationships. While chi-square tests identify whether the associations are statistically significant, Cramer’s V coefficients quantify their strength. As shown in Table 2, the Cramer’s V coefficients offer further insight, quantifying the strength of these associations and reinforcing the relevance of political factors in shaping Indigenous educational policies

**Table 2:** Strength of association between governor’s ideology and indigenous education practices (Cramer’s V).

Variables	Cramer’s V (0–1 scale)	Data Source
Indigenous Teaching Materials	0.32	Municipal Schools
Indigenous Languages in Classroom Instruction	0.17	Municipal Schools
Portuguese Language in Classroom Instruction	0.30	Municipal Schools
Both Indigenous and Portuguese Language in Classroom Instruction	0.33	Municipal Schools
Indigenous Teaching Materials	**0.92**	State Schools
Indigenous Languages in Classroom Instruction	**0.71**	State Schools
Portuguese Language in Classroom Instruction	**0.74**	State Schools
Both Indigenous and Portuguese Language in Classroom Instruction	**0.87**	State Schools

The analysis of Cramer’s V coefficients reveals significant differences in the strength of association between governors’ political ideologies and Indigenous educational practices across municipal and state networks. In municipal schools, associations are weak to moderate (Cramer’s V = 0.17–0.33), indicating that political ideology has a relatively limited impact on the use of Indigenous materials and languages. In contrast, state schools exhibit markedly stronger associations (Cramer’s V = 0.71–0.92), suggesting that governors’ ideological orientation plays a much more influential role in shaping the integration of Indigenous content and language instruction within state-level educational systems.

These differences align with institutional expectations: Brazilian state governments hold primary responsibility for curricular regulation, material adoption, and oversight of Indigenous education, while municipalities operate with comparatively less autonomy. As established in Brazilian educational legislation (1996), the allocation of resources for the preservation of Indigenous languages and cultural practices depends heavily on state-level priorities and political commitments [36,80].

More broadly, the findings point to a wider discontinuity in the use of Indigenous cultural resources across the school system. There has been a sharp decline in the use of Indigenous languages as the main medium of instruction in schools located within Indigenous territories. Although the magnitude of this decline varies regionally, the overall trajectory suggests that political dynamics at the state level shape the extent to which Indigenous linguistic and cultural components are preserved or eroded. Schools located in states governed by center-right, right, and especially far-right administrations are consistently less likely to adopt Indigenous pedagogical materials or Indigenous-language instruction compared to those under left or center-left governments. These patterns highlight how ideological positioning at the state level translates into concrete differences in the cultural orientation of Indigenous education.

**Fig 2:**
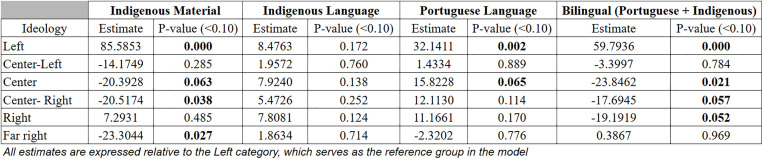
Effects of governors’ political ideology on indigenous educational practices in state-level schools.

Following the results of Cramer’s V test, as a robustness test, we evaluated the four educational indicators previously analyzed using hierarchical mixed-effects models. As shown in Fig 2, the use of Indigenous pedagogical materials exhibits the strongest and most consistent ideological gradients among all educational indicators. Left-governed states, used as the reference category, present a large intercept estimate (+85.6, p < 0.001). In contrast, states led by Center (–20.4, p ≈ 0.063), Center-Right (–20.5, p < 0.05), and Far-Right (–23.3, p < 0.05) administrations show significantly reduced adoption of these materials. This pattern indicates that culturally grounded pedagogical resources are substantially more prevalent under left-leaning governments, declining progressively as ideologies shift toward the center and the right.

Regarding Indigenous Language instruction, Fig 2 shows no statistically significant differences across ideological groups. Although the Left has a positive baseline estimate (+8.48), none of the comparisons for Center-Left, Center, Center-Right, Right, or Far-Right categories reach conventional levels of significance. This suggests that, unlike Indigenous pedagogical materials, the use of Indigenous language alone in classroom instruction is relatively stable and not strongly shaped by the ideological orientation of state governments.

The use of Portuguese Language as the exclusive medium of instruction shows a more nuanced ideological pattern in Fig 2. Left-leaning governments display a statistically positive baseline estimate (+32.1, p < 0.01), indicating higher prevalence of Portuguese-only instruction compared to other ideological categories. The Center group shows a marginally significant increase (+15.8, p ≈ 0.065), while the remaining ideological positions exhibit no meaningful difference from the reference. These results imply that Portuguese-only instruction is widely used across states

Finally, bilingual instruction (Indigenous + Portuguese) displays a clear political gradient in Fig 2. While the Left category exhibits a strong positive baseline (+59.8, p < 0.001), several groups show significantly or near-significantly lower adoption, including Center (–23.8, p < 0.05), Center-Right (–17.7, p ≈ 0.057), and Right (–19.2, p ≈ 0.052). These findings indicate that bilingual practices, which integrate Indigenous and Portuguese linguistic traditions, are meaningfully less prevalent under centrist and right-leaning governments. Compared to Indigenous Language alone, bilingual programs appear more politically sensitive and more strongly shaped by ideological positioning.

**Fig 3:**
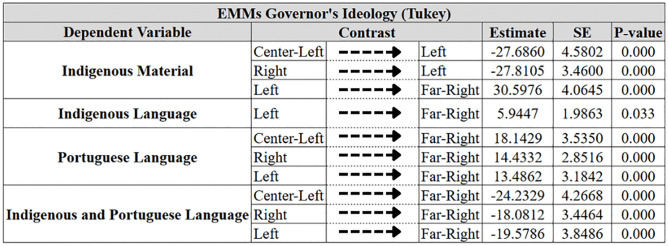
Tukey post-hoc comparisons of governor’s ideology effects on indigenous educational indicators.

The post-hoc Tukey contrasts presented in Fig 3 offer a more granular view of how ideological orientations translate into measurable differences in Indigenous and linguistic educational practices across Brazilian states. By comparing each ideological group directly against others, the table reveals not only whether ideology matters—as established in the main models—but also *where* the ideological distance is most consequential. These pairwise contrasts allow us to identify which ideological transitions produce the largest shifts in educational indicators, particularly regarding the use of Indigenous pedagogical materials, language-of-instruction patterns, and bilingual approaches. In this sense, Fig 3 strengthens the interpretation of the main findings by showing that differences are not uniformly distributed across the ideological spectrum but cluster around specific contrasts, especially those involving the Far-Right.

The results indicate that Center-Left and Right governments show significantly lower adoption of Indigenous pedagogical materials when compared to Left administrations, while the most pronounced gaps emerge in contrasts involving Far-Right states across nearly all indicators. The disparities between Left and Far-Right governments are especially substantial for the use of Indigenous Materials, Portuguese-only instruction, and Indigenous–Portuguese bilingual practices, signaling a systematic erosion of culturally grounded or intercultural educational approaches under ideologically extreme right-wing administrations. Conversely, contrasts that do not involve the Far-Right tend to show smaller or more selective differences. Taken together, these patterns underscore that ideological extremity—rather than simple left–right positioning—is the primary driver of reductions in support for Indigenous cultural content in schools. All detailed estimated marginal means, full contrast matrices, and additional robustness checks are available in the Supplementary Materials (S1 File).

**Fig 4:**
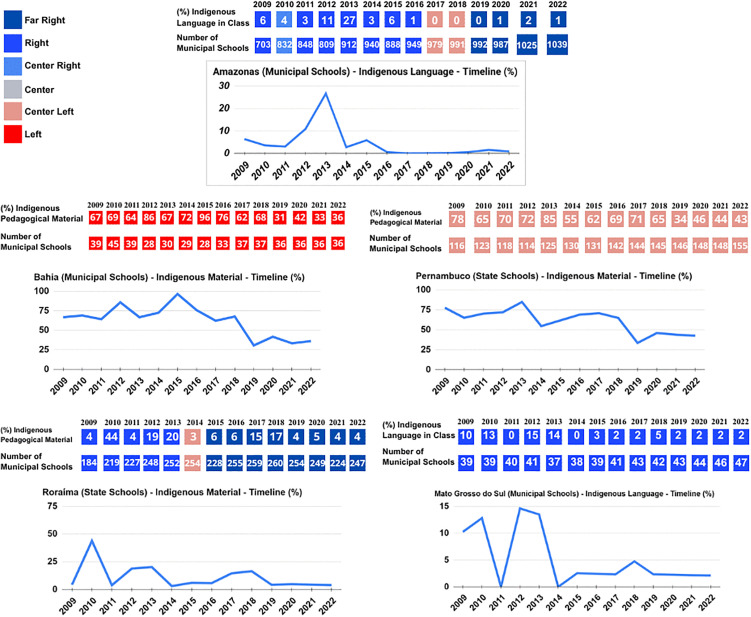
Trends in indigenous education indicators (2009–2022) across the five states with the largest indigenous populations.

Building on these nationwide patterns, we then narrowed the analysis to states with the largest Indigenous populations to better understand the spatial and temporal dynamics underlying these ideological effects. By examining longitudinal trends in the use of Indigenous teaching materials and language instruction, we sought to determine whether the erosion of Indigenous content occurred uniformly across regions or was concentrated in specific political and demographic contexts. These trajectories—together with the evolution of the number of Indigenous schools in each state—are presented in Fig 1 and further detailed in Fig 4.

An analysis of the data presented in Figure 4 reveals that the states of Amazonas and Mato Grosso do Sul have consistently neglected the use of Indigenous languages in classroom instruction. Over several years, this has led to a substantial—and, in some cases, complete—decline in the use of native languages within educational settings. Collectively, these two states account for approximately 33% of Brazil’s Indigenous population, making this decline particularly concerning in terms of linguistic preservation and cultural continuity. Amazonas and Mato Grosso do Sul have been governed predominantly by right-wing and far-right political forces over the past decade, with these ideological alignments appearing to be directly associated with the educational outcomes identified in this study. The trend underscores the urgent need for policies that actively promote and protect linguistic diversity among Indigenous communities.

**Fig 5:**
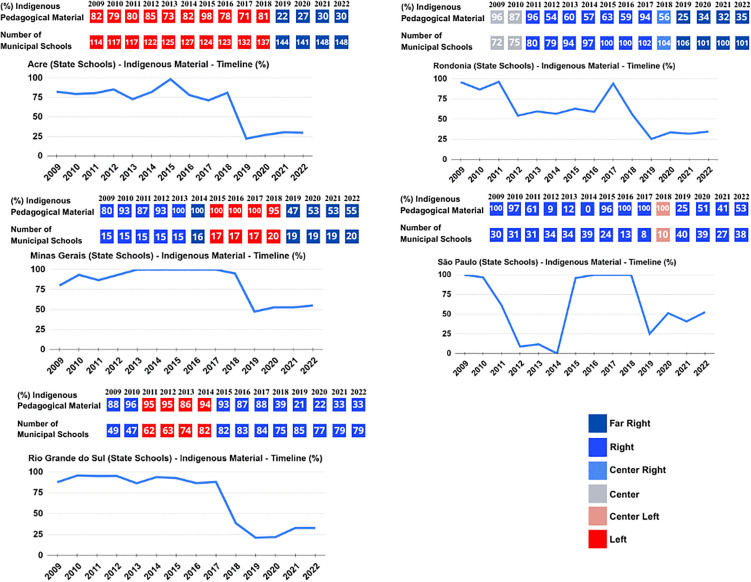
States exhibiting a strong influence of right-wing political forces.

In contrast, the states of Bahia and Pernambuco—home to approximately 17% of the country’s Indigenous population—have historically been governed by left-leaning and center-left administrations. However, since 2019, these states have also experienced a significant reduction—by nearly half—in the use of Indigenous educational materials in classrooms. A closer examination of Roraima, which has been under far-right governance since 2015, reveals a persistently low level of Indigenous content integration within the curriculum. By 2022, only 4% of schools in the state had adopted culturally relevant educational materials. Similar to Roraima, other states influenced by right-wing political forces exhibit clear patterns of deprioritizing Indigenous culture within school curricula, as illustrated in Fig 5.

The examination of Fig 5 offers significant insights, demonstrating that left-leaning administrations in the regions of Acre, Rio Grande do Sul, São Paulo, and Minas Gerais successfully maintained Indigenous content in no less than 70% of their educational institutions throughout the period under analysis. Another key observation from Fig 3 is the sharp decline in Indigenous content across all depicted states, coinciding with the inauguration of right-wing and far-right politicians elected during the 2018 elections, an era commonly referred to as the “far-right wave.”

This effect, observable since 2018, contrasts sharply with the prior political orientation of governors known for aligning themselves with far-right agendas, such as those in Rio Grande do Sul, Minas Gerais, São Paulo, Acre, and Rondônia. These states collectively represent approximately 17% of Brazil’s Indigenous population.

Building upon these findings, our investigation sought to assess the impact of what is commonly referred to as the “far-right hard core.” This group includes prominent figures such as the governors of Roraima, Goiás, Acre, Mato Grosso, Amazonas, and Rondônia—six governors closely aligned with the ideological principles of the far-right. Collectively, these states account for 43% of Brazil’s Indigenous population.

**Fig 6:**
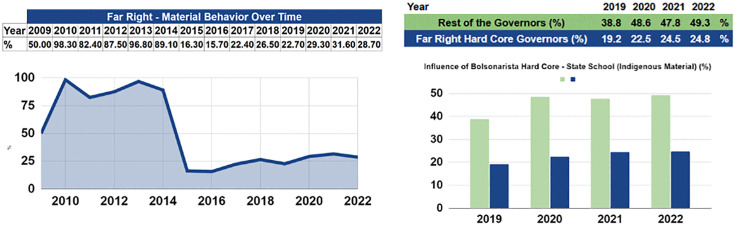
Longitudinal impact of the far-right hard core and broader right-wing spectrum.

In light of this, we undertook an analysis to determine whether this specific group of governors exerted a distinctive influence on the curtailment of Indigenous initiatives when compared to other governors, as illustrated in Fig 6

Upon examining the graphical data, it is clear that the far-right hard core exerts a distinctive and pronounced influence. Compared to other Brazilian governors, this influence highlights a stark disparity affecting Indigenous schools in these states. Specifically, Indigenous educational institutions under the administration of these far-right governors experienced a twofold greater detriment relative to other regions in Brazil.

As shown in Fig 6, we investigated the impact of far-right ideology on the use of Indigenous curricula in the evaluated schools from 2009 to 2022. A striking pattern emerged: the rise of Brazil’s extreme right took on unprecedented historical significance beginning in 2015. This period of radicalization sharply contrasts with the relatively stable ideological environment observed from 2009 to 2014, underscoring a profound shift in political orientation.

**Fig 7:**
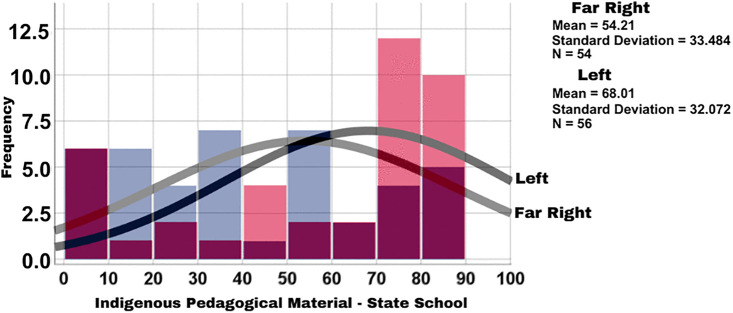
Comparative analysis of far-right and left-wing influence on indigenous educational materials in indigenous schools.

Finally, we compared the behavior of both the far-right and left-leaning governments regarding the incorporation of Indigenous pedagogical resources within schools over the study period, as illustrated in Fig 7.

The data reveal a profound dichotomy in Brazil, contrasting left-leaning and far-right ideologies regarding the inclusion of Indigenous content in educational settings. Incorporating Indigenous knowledge and culture is a vital way to honor and preserve Indigenous heritage, as explicitly mandated by the 1988 Brazilian Constitution (CF-88). The findings of this study consistently demonstrate that far-right ideology has hindered the progress and well-being of Indigenous communities [81,82].

This study was exceptionally comprehensive, examining the impact of political ideologies across all Brazilian states and Indigenous schools from 2009 to 2022. Due to space constraints, not all of these patterns are presented here; however, they can be viewed in the Supplementary Material, in Figures S01 to S043 (S1 File). A thorough analysis of these additional figures may offer even deeper insights into the themes discussed in this research.

## Discussion

These findings can be situated within a broader body of scholarship on Indigenous education and political ideology in Brazil. Previous studies emphasize that Indigenous schooling has long oscillated between emancipatory and assimilationist paradigms, depending on the ideological orientation of those in power [31,37]. Our results empirically substantiate this claim, showing that left-wing administrations tend to safeguard intercultural and bilingual education, while right-wing and far-right governments often reproduce assimilationist tendencies. Similar patterns have been observed in studies highlighting the persistent influence of political agendas in defining the cultural content of Indigenous education [32,52]. By quantitatively demonstrating these ideological effects, our study bridges the gap between qualitative historical analyses and contemporary statistical evidence.

In Brazil, an extensive body of Indigenous intellectual and literary production provides essential perspectives for understanding the cultural stakes of Indigenous educational policy. Authors such as Daniel Munduruku, Davi Kopenawa Yanomami, Ailton Krenak, Auritha Tabajara, Eliane Potiguara, Eva Potiguara, Jerá Poty Mirim, and Jama Wapichana articulate—through literature, philosophy, children’s books, environmental thought, poetry, and oral tradition—the centrality of language, memory, and cosmology in Indigenous identity. Works such as Kopenawa’s The Falling Sky: Words of a Yanomami Shaman (A Queda do Céu: Palavras de um Xamã Yanomami) and Krenak’s Ideas to Postpone the End of the World (Ideias para Adiar o Fim do Mundo) have had profound influence in contemporary debates about colonialism, environmental destruction, and the erosion of Indigenous ways of life. Similarly, Munduruku’s writings for children and young people highlight Indigenous cultural diversity; Tabajara’s cordel literature and the works of the Potiguara authors foreground environmental education, music, and ancestry; while Wapichana and Poty Mirim contribute to Indigenous literary criticism and visual culture. Together, these voices illustrate the cultural richness at risk when schooling fails to incorporate Indigenous epistemologies and underscore how educational systems, when disconnected from Indigenous knowledge, can function as instruments of cultural erosion. Integrating these intellectual contributions into future research would deepen the qualitative understanding of how linguistic and pedagogical suppression affects Indigenous communities across Brazil [83–85].

The effort to assimilate Indigenous peoples into the broader Brazilian society is not an isolated phenomenon; similar initiatives have been implemented in various other nations. However, over time, the detrimental consequences of such policies have become evident, as they have impeded the advancement and well-being of these populations [86–93].

Indigenous groups are among the most marginalized and disadvantaged minorities within developed nations. In countries such as Australia, Canada, New Zealand, and the United States, they continue to grapple with significant disparities in educational attainment among Indigenous populations [91].

The consequences of the systemic disenfranchisement has profound physical and emotional impacts on the youth. These policies have limited their access to higher education, eroded cultural heritage and linguistic diversity, fostered deep alienation from familial and community support networks, and contributed to patterns of substance abuse—ultimately leading to the persistent socioeconomic marginalization of these communities [86–93].

As a result, such public policies have profoundly reshaped the internal dynamics of Indigenous societies and influenced subsequent governance frameworks, fundamentally altering how these communities have been managed and regulated [86–93].

Recognizing the role of Indigenous peoples in shaping our collective future, particularly in advancing sustainability, is decisive. Over the past three decades, there has been an increased awareness of the environmentally destructive consequences of market economy development. This growing consciousness has prompted the search for alternative practices that exert less ecological harm [94,95].

Within this context, Indigenous communities, unlike the industrialized societies responsible for most contemporary climate impacts over the past two centuries, have sustainably inhabited their ancestral lands for millennia and bear minimal responsibility for current and future environmental crises. Some scholars persuasively argue that much of the planet’s environmental and ecological wealth is safeguarded within Indigenous territories, underscoring the vital contributions these communities make to global biodiversity conservation [96].

While Indigenous schools are formally managed by the communities themselves, their ability to sustain bilingual programs and culturally tailored materials depends heavily on external funding, training, and curricular support provided by state and municipal governments. Thus, the absence of Indigenous language instruction or materials cannot be attributed solely to local school decisions but must also be understood in light of broader structural constraints. Limited financial and institutional capacity often intersects with political factors, since the allocation of educational resources and the prioritization of intercultural education are contingent upon the ideological orientation of those in power. In this sense, ideological imposition and structural underfunding are not mutually exclusive explanations; rather, they operate simultaneously, with ideological agendas shaping both the level and the direction of public investment in Indigenous education.

Nonetheless, as several studies have observed, the implementation of intercultural education also depends on structural conditions—such as teacher training, local participation, and financial resources—that vary widely across regions [10,40]. Therefore, while our results confirm a strong ideological component, they should also be interpreted in light of these broader institutional constraints. This intersection between ideology and capacity-building constitutes a promising avenue for future research, particularly through mixed-method designs that combine large-scale data with ethnographic inquiry.

## Conclusion

This study provides a comprehensive, longitudinal assessment of how political ideology shapes Indigenous education in Brazil from 2009 to 2022. Drawing on nationwide School Census microdata, it demonstrates that the ideological orientation of governors exerts a decisive influence on the implementation of culturally responsive education. Left-wing administrations have tended to sustain Indigenous language instruction and culturally grounded materials, whereas right-wing and far-right governments have systematically curtailed them. By integrating statistical evidence with political analysis, the research advances empirical understanding of how ideological polarization translates into tangible educational outcomes.

Theoretically, these findings contribute to the growing body of literature examining the intersection of political ideology and multicultural policy implementation. They substantiate claims that education in Indigenous territories functions both as a field of cultural preservation and as a battleground for ideological contestation [10,22–26,28–34,37,38,40,41,46]. Our evidence extends these insights by demonstrating, through quantitative methods, that the erosion of Indigenous education is not an abstract process of cultural loss but a measurable manifestation of political choices and governance priorities. The results thus position ideology not merely as background context but as an active determinant of how constitutional guarantees of bilingual and intercultural education are realized—or undermined—across Brazil’s federative system.

Methodologically, the study contributes to policy analysis by operationalizing ideological spectra and educational outcomes through binary indicators derived from administrative data. This approach allows for reproducibility and large-scale inference, while also illustrating the challenges inherent in using categorical variables to represent complex cultural phenomena. Future work should integrate mixed methods—linking statistical models with ethnographic fieldwork, fiscal data, and interviews—to capture the mechanisms through which ideology interacts with funding structures, teacher training, and community agency. Such integration would clarify whether policy discontinuities stem primarily from ideological hostility, administrative neglect, or material scarcity.

Some limitations must be acknowledged. The data do not capture qualitative dimensions of school governance, curricular autonomy, or the extent of Indigenous participation in educational decision-making. Moreover, while the use of binary variables enables national coverage, it simplifies the spectrum of intercultural practices, potentially underestimating local variation. Addressing these limitations in future research will deepen our understanding of how state ideology and community resilience jointly shape the trajectory of Indigenous education.

## Supporting information

S1 File Supplementary information and extended analysis. This document contains 49 pages of comprehensive statistical analysis and robustness checks, including supplementary figures S01 to S44 and S48 to S51.
(PDF)
