# Supplementary material for: The impact of political ideologies on cultural erosion: Indigenous education policy in Brazil (2009–2022)
Source: PLoS One. 2026 Jul 23;21(7):e0354262. doi: 10.1371/journal.pone.0354262 (PMC13395449; doi:10.1371/journal.pone.0354262)
Supplement: S1 File — This document contains 49 pages of comprehensive statistical analysis and robustness checks, including supplementary figures S01 to S44 and S48 to S51. (PDF) [file pone.0354262.s001.pdf]

Supplementary Materials for The Impact of Political Ideologies on Cultural Erosion: Indigenous Education Policy and Management in Brazil (2009–2022)

André Calixto Gonçalves;
Rodolfo Valentim
Francisco Aparecido Rodrigues;
Guilherme Antônio de Almeida Lopes Fernandes
Ivan Filipe de Almeida Lopes Fernandes;

Corresponding author: andre.calixto.goncalves@gmail.com

State of Amazonas

Figure S01

Legend (colour key): Far Right; Right; Center Right; Center; Center Left; Left

Indigenous Material - State Schools

	2009	2010	2011	2012	2013	2014	2015	2016	2017	2018	2019	2020	2021	2022	Year
AM	68,18	27,27	39,13	4,55	14,29	4,17	11,63	22,58	7,84	17,95	19,23	15,09	17,86	16,98	%

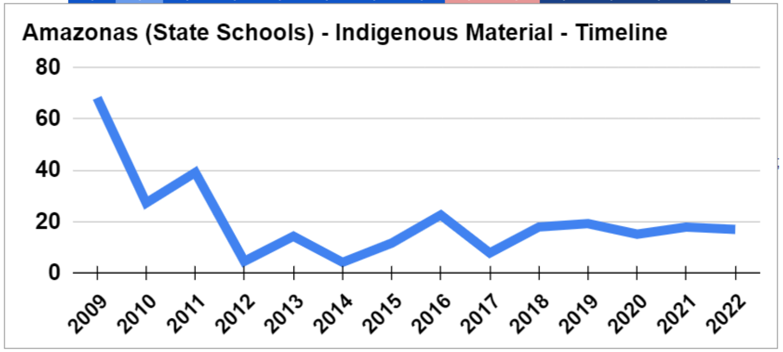

Indigenous Material - Municipal Schools

	2009	2010	2011	2012	2013	2014	2015	2016	2017	2018	2019	2020	2021	2022	Year
AM	24,04	49,28	37,03	55,13	60,09	40,96	50,79	46,36	40,14	48,54	30,34	38,4	44,49	46,29	%

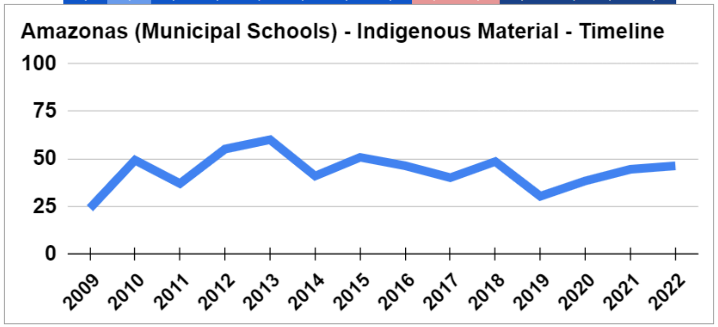

Indigenous Language - State Schools

	2009	2010	2011	2012	2013	2014	2015	2016	2017	2018	2019	2020	2021	2022	Year
AM	36,06	0	0	0	14,29	4,17	2,33	0	3,92	0	0	1,89	3,57	1,89	%

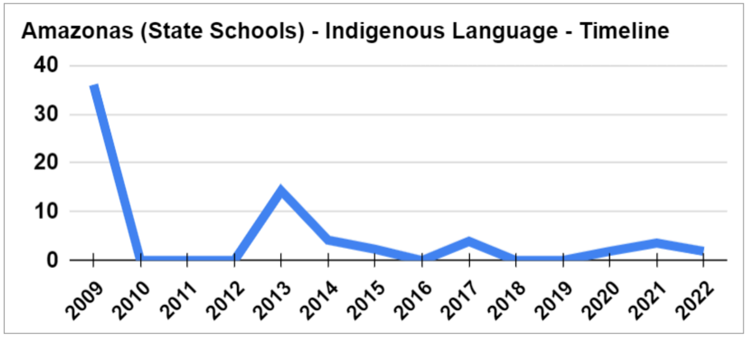

Indigenous Language - Municipal Schools

	2009	2010	2011	2012	2013	2014	2015	2016	2017	2018	2019	2020	2021	2022	Year
AM	6,4	3,61	3,07	10,88	26,64	2,77	5,86	0,63	0	0,1	0,2	0,61	1,56	0,87	%

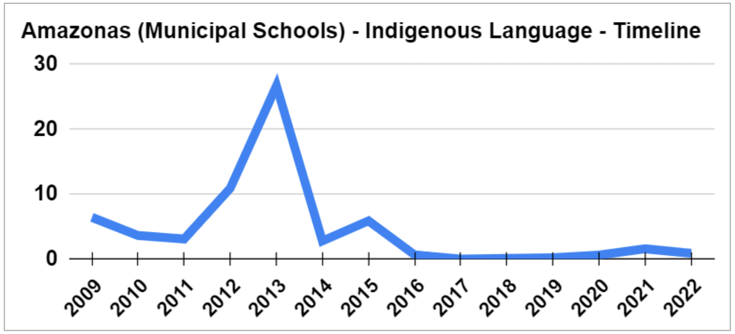

Number of State Schools

	2009	2010	2011	2012	2013	2014	2015	2016	2017	2018	2019	2020	2021	2022	Year
AM	22	22	23	22	21	24	43	31	51	39	52	53	56	53	n

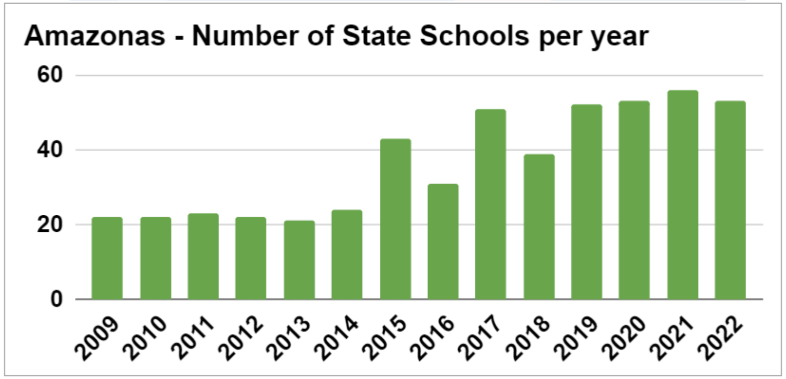

Number of Municipal Schools

	2009	2010	2011	2012	2013	2014	2015	2016	2017	2018	2019	2020	2021	2022	Year
AM	703	832	848	809	912	940	888	949	979	991	992	987	1025	1039	n

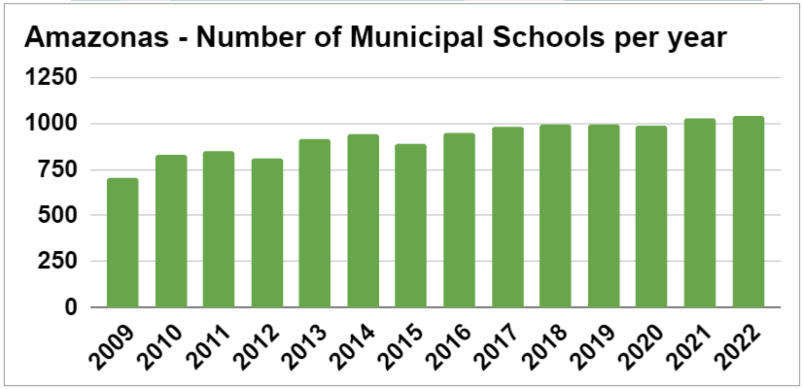

State of Acre

Figure S02

- Far Right
- Right
- Center Right
- Center
- Center Left
- Left

Indigenous Material - State Schools

	2009	2010	2011	2012	2013	2014	2015	2016	2017	2018	2019	2020	2021	2022	Year
AC	82,46	79,49	80,34	85,25	72,8	81,89	98,39	78,05	71,21	81,02	22,22	26,95	30,41	29,73	%

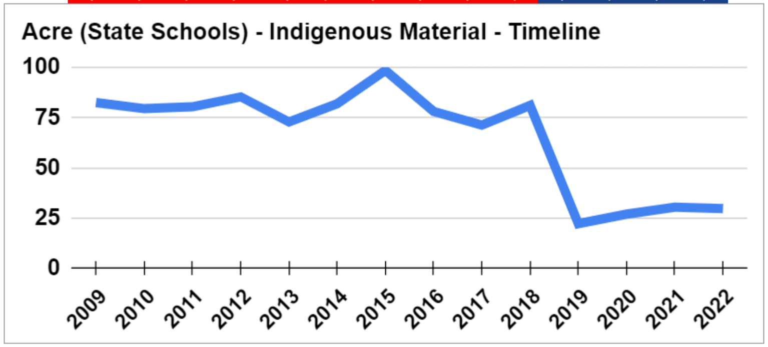

Indigenous Material - Municipal Schools

	2009	2010	2011	2012	2013	2014	2015	2016	2017	2018	2019	2020	2021	2022	Year
AC	82,98	95	50	91,18	57,75	56,94	12,33	13,92	55,7	6,17	0	13,58	11,11	11,11	%

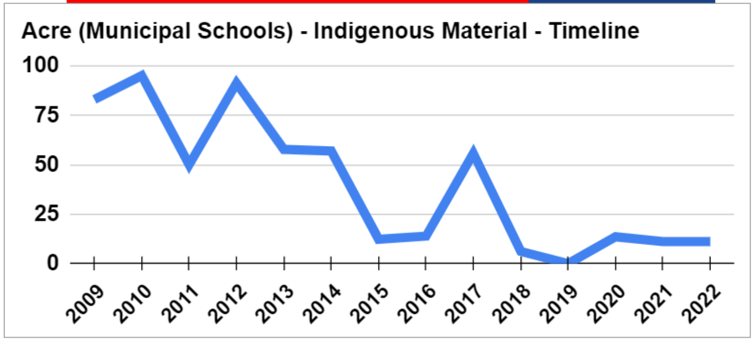

Indigenous Language - State Schools

	2009	2010	2011	2012	2013	2014	2015	2016	2017	2018	2019	2020	2021	2022	Year
AC	28,07	21,37	5,98	25,41	31,2	25,2	14,52	3,25	3,79	0	0,69	1,42	1,35	1,35	%

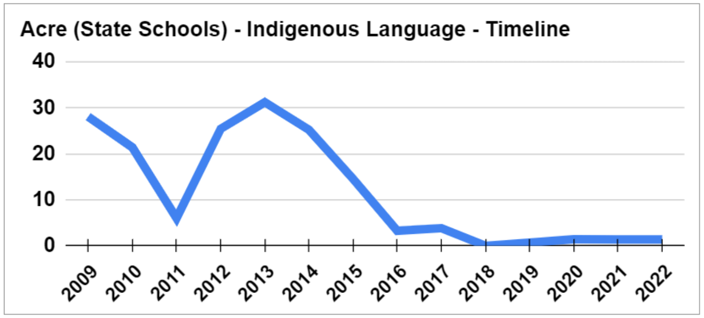

Indigenous Language - Municipal Schools

Lingua Indigena - Escolas Municipais

	2009	2010	2011	2012	2013	2014	2015	2016	2017	2018	2019	2020	2021	2022	Year
AC	2,13	48,33	3,23	5,88	4,23	1,39	46,58	44,3	31,65	8,64	6,1	4,94	4,94	4,94	%

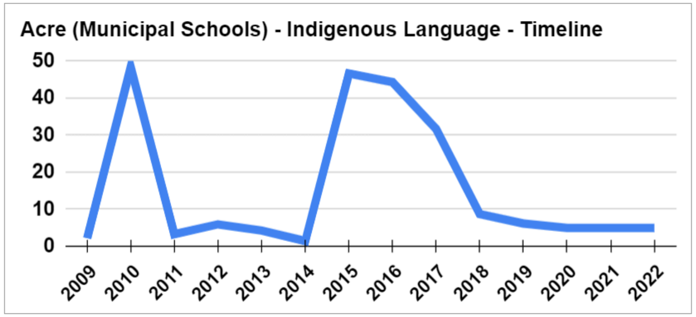

Number of State Schools

	2009	2010	2011	2012	2013	2014	2015	2016	2017	2018	2019	2020	2021	2022	Year
AC	114	117	117	122	125	127	124	123	132	137	144	141	148	148	n

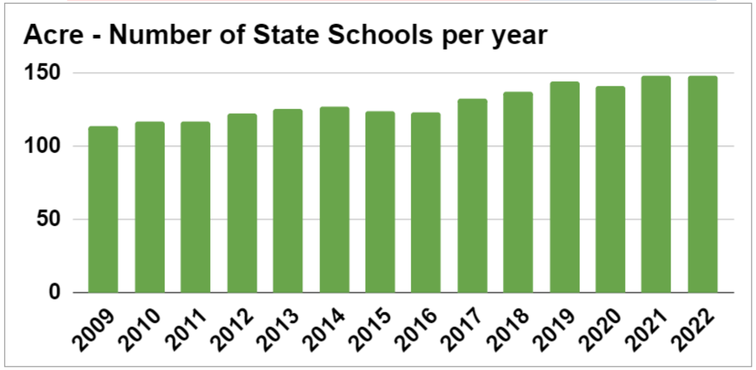

Number of Municipal Schools

	2009	2010	2011	2012	2013	2014	2015	2016	2017	2018	2019	2020	2021	2022	Year
AC	12	12	12	12	12	12	12	12	12	12	12	12	12	12	n

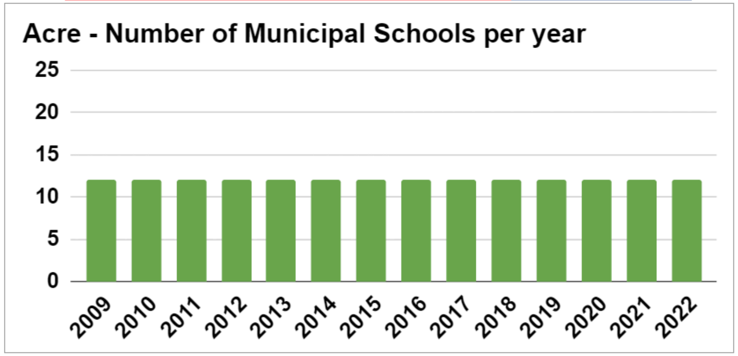

State of Roraima

Figure S03

	Far Right
	Right
	Center Right
	Center
	Center Left
	Left

Indigenous Material - State Schools

	2009	2010	2011	2012	2013	2014	2015	2016	2017	2018	2019	2020	2021	2022	Year
RR	4,35	43,84	3,96	18,95	20,24	3,15	6,14	5,88	14,67	16,54	4,33	4,82	4,46	4,05	%

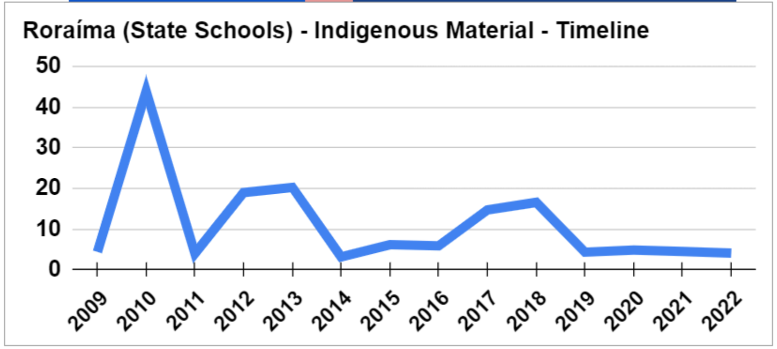

Indigenous Material - Municipal Schools

	2009	2010	2011	2012	2013	2014	2015	2016	2017	2018	2019	2020	2021	2022	Year
RR	10,2	17,65	5	23,44	21,25	13,73	18,79	26,53	0,78	19,08	2,13	6,67	4,64	5,39	%

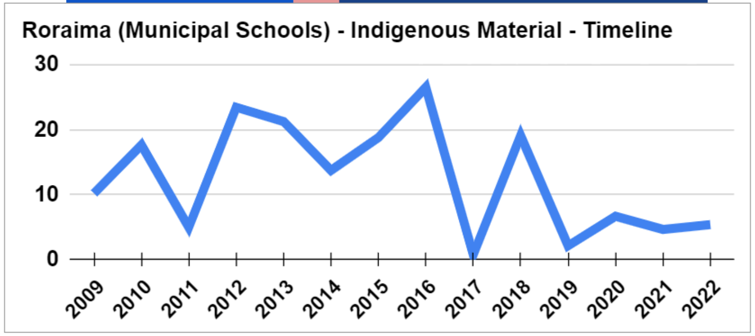

Indigenous Language - State Schools

	2009	2010	2011	2012	2013	2014	2015	2016	2017	2018	2019	2020	2021	2022	Year
RR	2,17	4,11	7,93	2,82	17,86	15,35	1,75	1,96	0,77	0,38	0,39	0,4	0,89	0,4	%

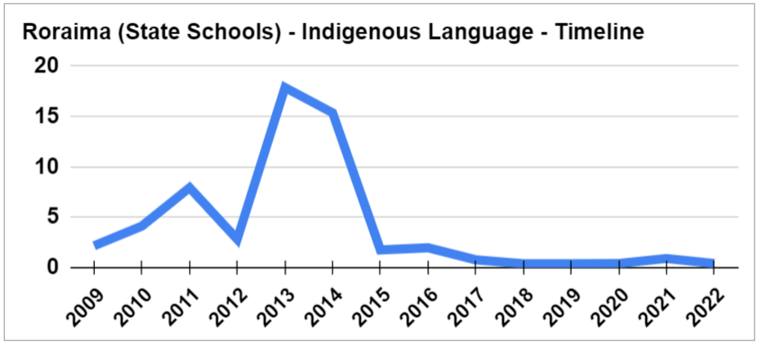

Indigenous Language - Municipal Schools

	2009	2010	2011	2012	2013	2014	2015	2016	2017	2018	2019	2020	2021	2022	Year
RR	0	0	3,33	12,5	21,25	13,73	3,66	0	0	0	0	0	0	0	%

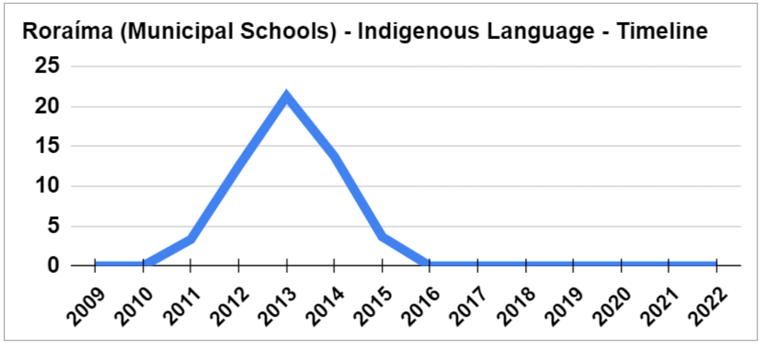

Number of State Schools

	2009	2010	2011	2012	2013	2014	2015	2016	2017	2018	2019	2020	2021	2022	Year
RR	184	219	227	248	252	254	228	255	259	260	254	249	224	247	n

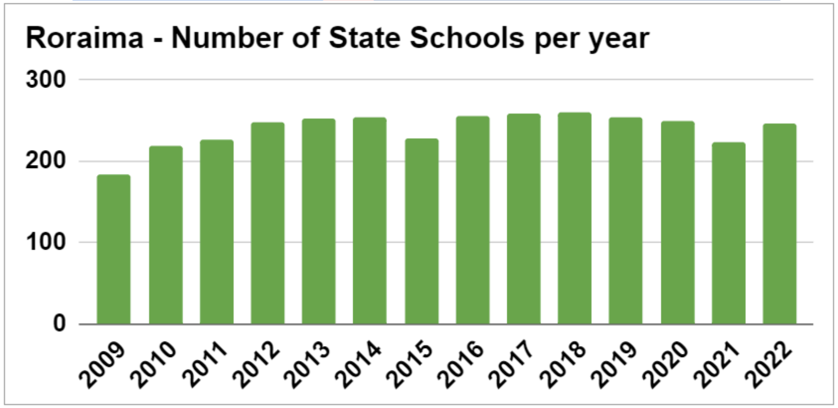

Number of Municipal Schools

	2009	2010	2011	2012	2013	2014	2015	2016	2017	2018	2019	2020	2021	2022	Year
RR	14	14	14	14	14	14	14	14	14	14	14	14	14	14	n

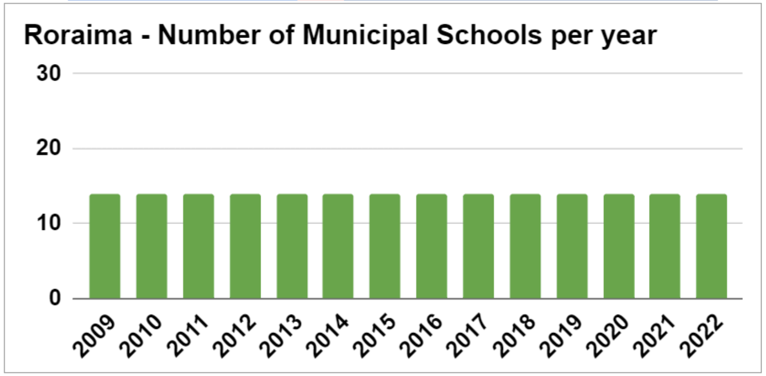

State of Rondonia

Figure S04

Legend
Far Right
Right
Center Right
Center
Center Left
Left

Indigenous Material - State Schools

	2009	2010	2011	2012	2013	2014	2015	2016	2017	2018	2019	2020	2021	2022	Year
RO	95,83	86,67	96,25	54,43	59,57	56,7	63	59	94,12	55,77	25,47	33,66	32	34,65	%

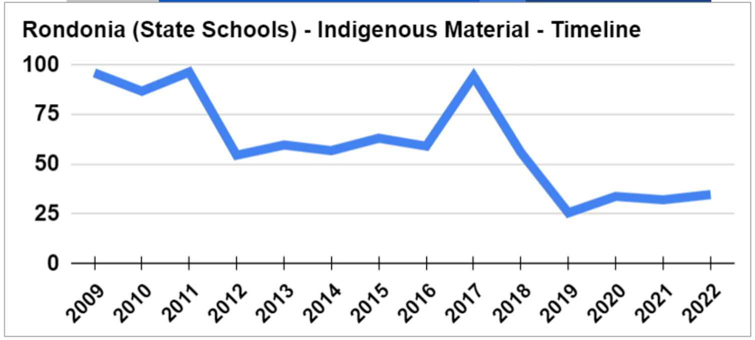

Indigenous Material - Municipal Schools

	2009	2010	2011	2012	2013	2014	2015	2016	2017	2018	2019	2020	2021	2022	Year
RO	40	40	66,67	100	100	0	50	0	0						%

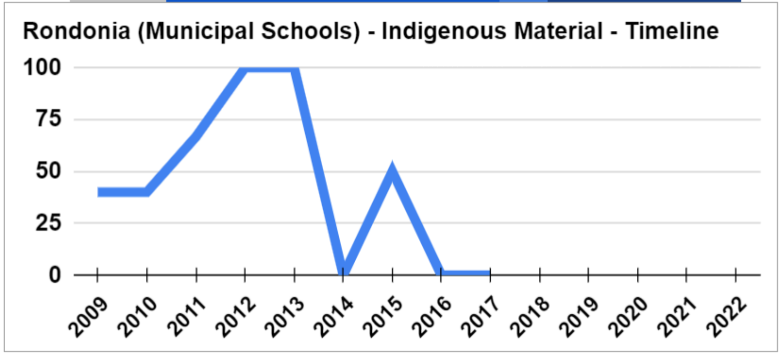

Indigenous Language - State Schools

	2009	2010	2011	2012	2013	2014	2015	2016	2017	2018	2019	2020	2021	2022	Year
RO	0	1,33	1,25	12,66	11,7	14,43	5	2	0	1,92	2,83	1,98	2	1,98	%

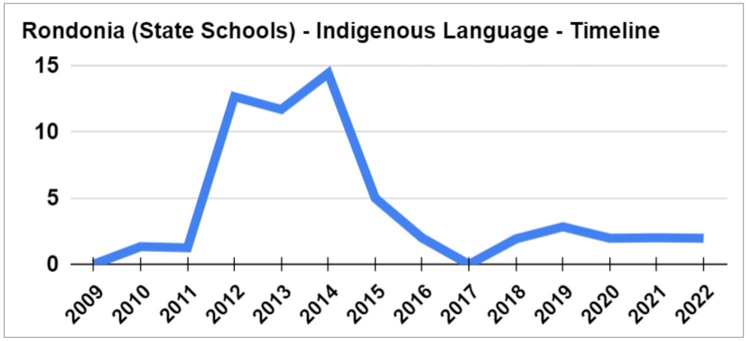

Indigenous Language - Municipal Schools

	2009	2010	2011	2012	2013	2014	2015	2016	2017	2018	2019	2020	2021	2022	Year
RO	0	0	0	0	0	0	0	0	0						%

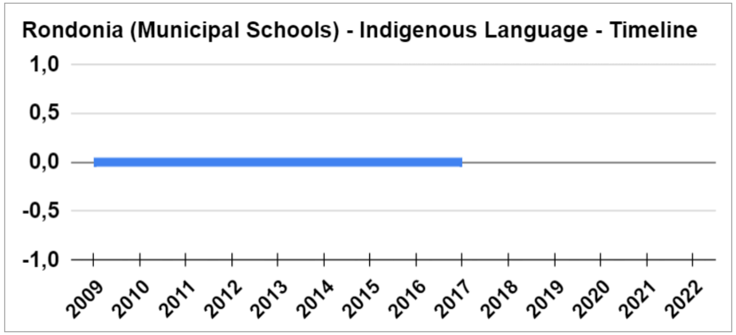

Number of State Schools

	2009	2010	2011	2012	2013	2014	2015	2016	2017	2018	2019	2020	2021	2022	Year
RO	72	75	80	79	94	97	100	100	102	104	106	101	100	101	n

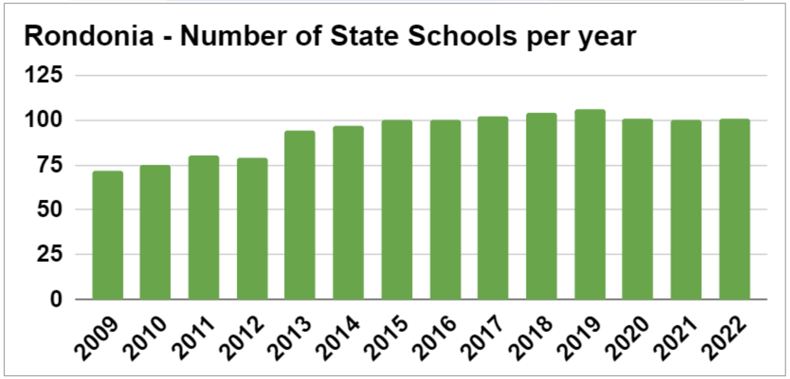

Number of Municipal Schools

	2009	2010	2011	2012	2013	2014	2015	2016	2017	2018	2019	2020	2021	2022	Year
RO	5	5	3	3	3	2	2	2	2						n

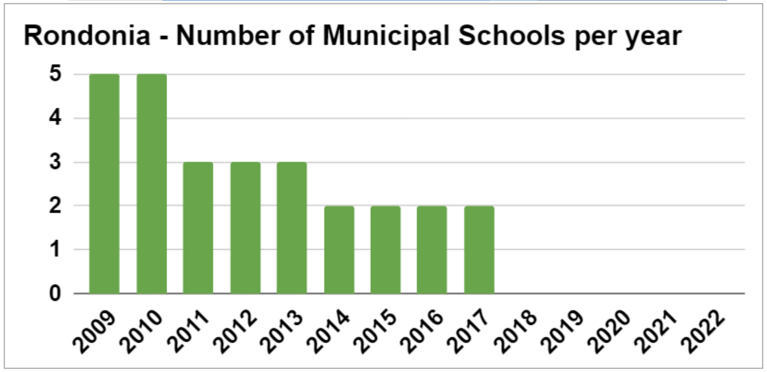

State of Pará

Figure S05

Indigenous Material - State Schools

	2009	2010	2011	2012	2013	2014	2015	2016	2017	2018	2019	2020	2021	2022	Year
PA	40	20	14,29	0	11,11	0	62,5	50	100	30,77	22,22	50	0	0	%

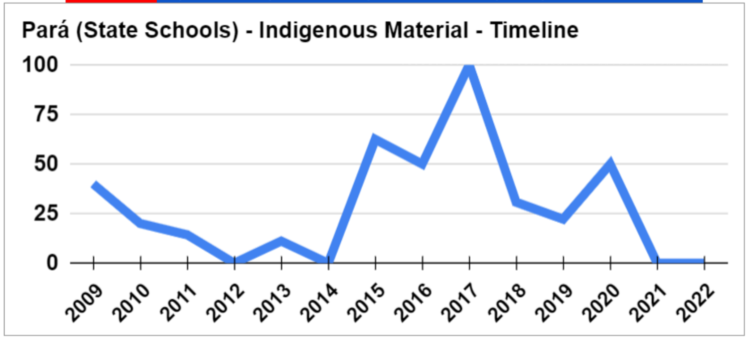

Indigenous Material - Municipal Schools

	2009	2010	2011	2012	2013	2014	2015	2016	2017	2018	2019	2020	2021	2022	Year
PA	48,8	37,6	43,66	54,61	57,5	50	59,39	48,85	53,4	52,61	29,82	33,33	36,29	44,07	%

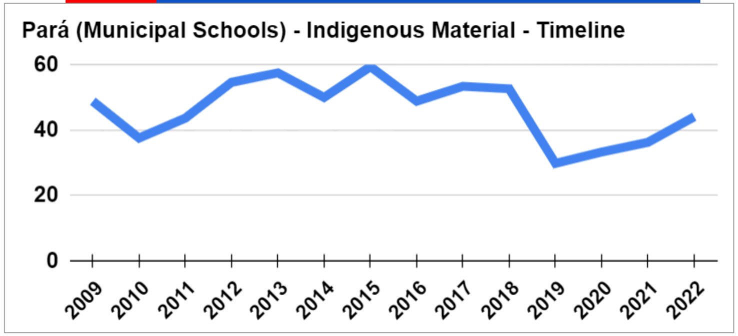

Indigenous Language - State Schools

	2009	2010	2011	2012	2013	2014	2015	2016	2017	2018	2019	2020	2021	2022	Year
PA	20	20	0	0	0	0	0	0	0	7,69	0	20	31,82	0	%

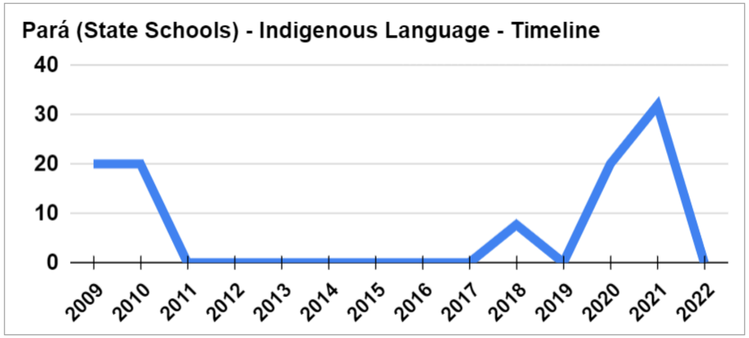

Indigenous Language - Municipal Schools

	2009	2010	2011	2012	2013	2014	2015	2016	2017	2018	2019	2020	2021	2022	Year
PA	7,2	4,8	7,04	7,89	5,63	6,63	5,45	4,02	4,71	4,27	4,13	5,13	4,44	4,44	%

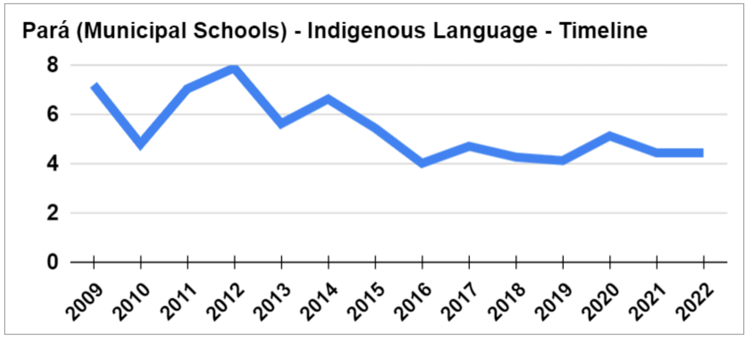

Number of State Schools

	2009	2010	2011	2012	2013	2014	2015	2016	2017	2018	2019	2020	2021	2022	Year
PA	5	5	7	8	9	7	8	4	9	13	18	10	22	20	n

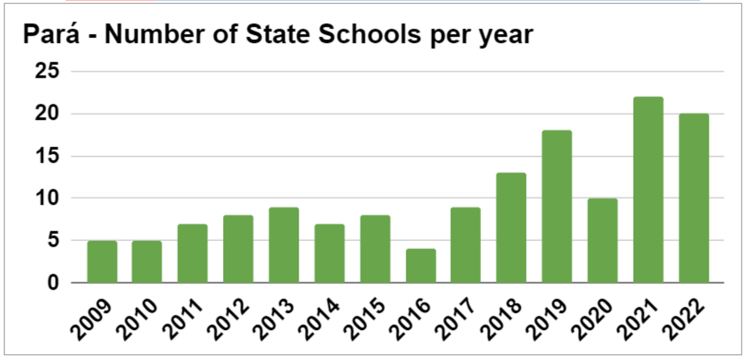

Number of Municipal Schools

	2009	2010	2011	2012	2013	2014	2015	2016	2017	2018	2019	2020	2021	2022	Year
PA	125	125	142	152	160	166	165	174	191	211	218	234	248	270	n

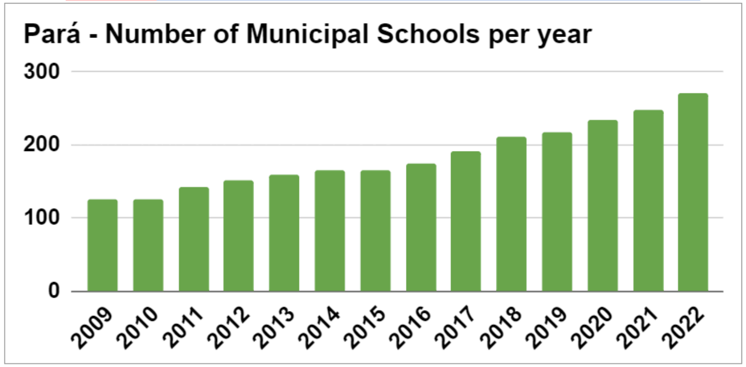

State of Amapá

Figure S06

Far Right
Right
Center Right
Center
Center Left
Left

Indigenous Material - State Schools

	2009	2010	2011	2012	2013	2014	2015	2016	2017	2018	2019	2020	2021	2022	Year
AP	94,64	98,21	89,09	88,89	87,27	85,19	87,23	88,89	87,04	90,74	74,07	79,63	81,13	77,78	%

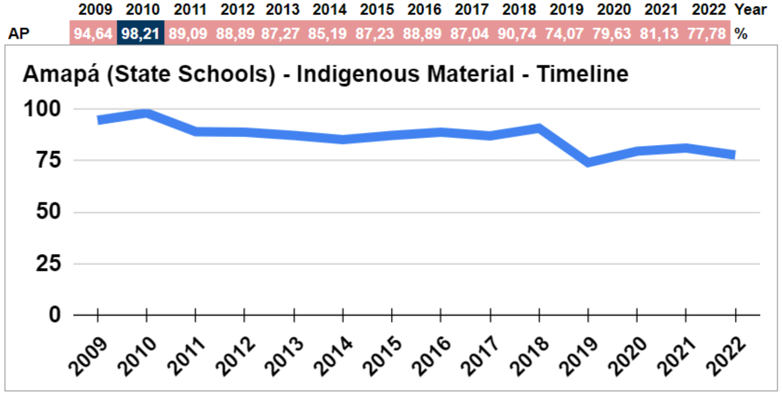

Indigenous Material - Municipal Schools

	2009	2010	2011	2012	2013	2014	2015	2016	2017	2018	2019	2020	2021	2022	Year
AP	100	100	66,67	100	90	10	100	0	87,5	100	12,5	12,5	0	0	%

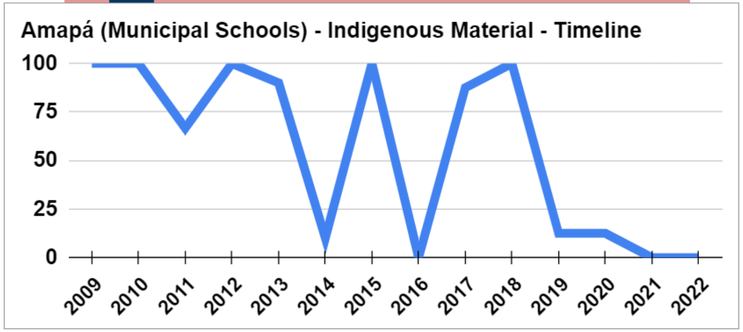

Indigenous Language - State Schools

	2009	2010	2011	2012	2013	2014	2015	2016	2017	2018	2019	2020	2021	2022	Year
AP	20	20	0	0	0	0	0	0	0	7,69	0	20	31,82	0	%

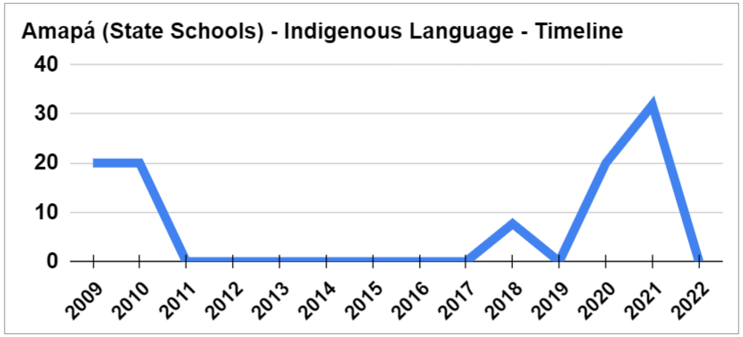

Indigenous Language - Municipal Schools

	2009	2010	2011	2012	2013	2014	2015	2016	2017	2018	2019	2020	2021	2022	Year
AP	0	0	0	40	0	0	57,14	20	0	0	0	0	0	0	%

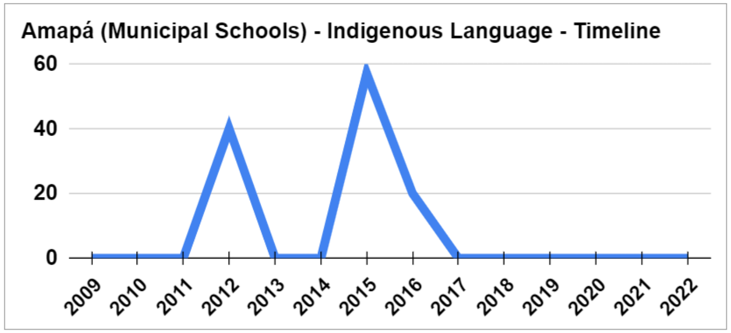

Number of State Schools

	2009	2010	2011	2012	2013	2014	2015	2016	2017	2018	2019	2020	2021	2022	Year
AP	56	56	55	54	55	54	47	54	54	54	54	54	53	54	n

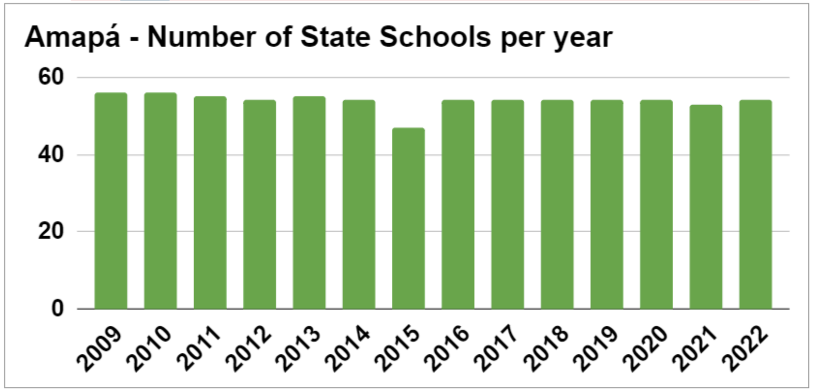

Number of Municipal Schools

	2009	2010	2011	2012	2013	2014	2015	2016	2017	2018	2019	2020	2021	2022	Year
AP	1	2	3	5	10	10	7	5	8	8	8	8	8	8	n

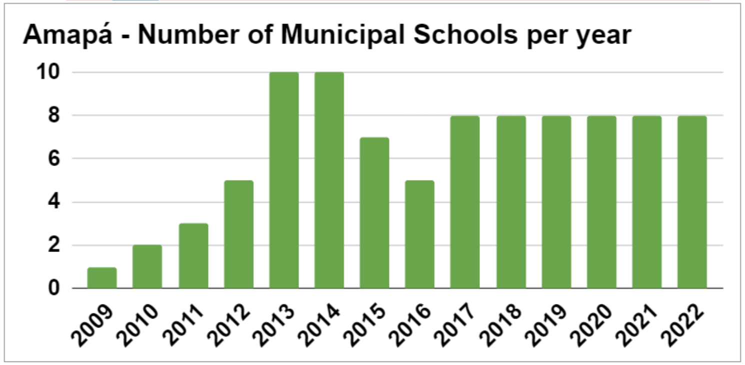

State of Tocantins

Figure S07

- Far Right
- Right
- Center Right
- Center
- Center Left
- Left

Indigenous Material - State Schools

	2009	2010	2011	2012	2013	2014	2015	2016	2017	2018	2019	2020	2021	2022	Year
TO	56,52	70,53	89,47	91,36	95	90,24	78,31	72,62	90	92,47	47,31	55,32	59,57	54,08	%

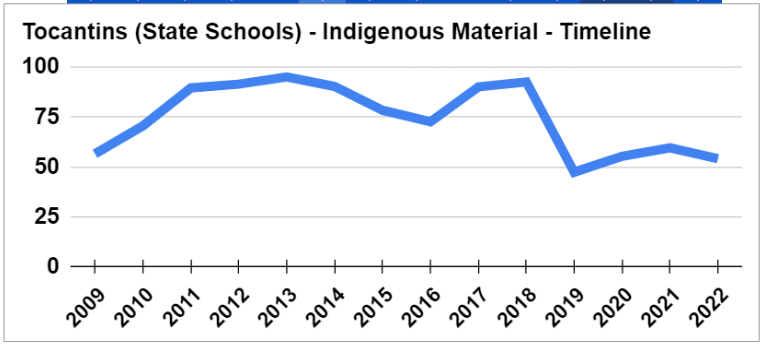

Indigenous Material - Municipal Schools

	2009	2010	2011	2012	2013	2014	2015	2016	2017	2018	2019	2020	2021	2022	Year
TO						100		100	66,67	100	33,33	33,33	0	66,67	%

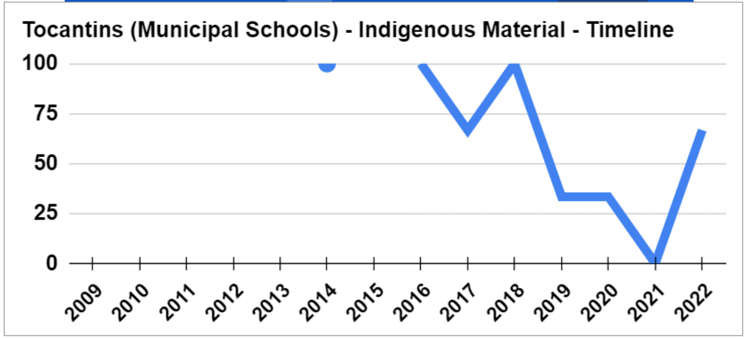

Indigenous Language - State Schools

	2009	2010	2011	2012	2013	2014	2015	2016	2017	2018	2019	2020	2021	2022	Year
TO	5,43	7,37	3,16	12,35	2,5	2,44	2,41	0	0	1,08	3,23	3,19	3,19	2,04	%

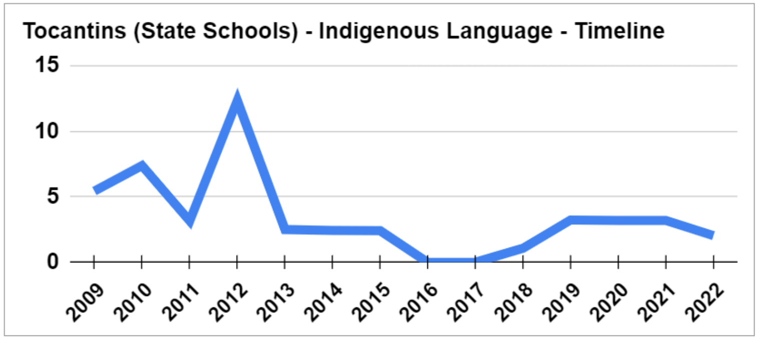

Indigenous Language - Municipal Schools

	2009	2010	2011	2012	2013	2014	2015	2016	2017	2018	2019	2020	2021	2022	Year
TO						50		0	0	0	0	0	0	0	%

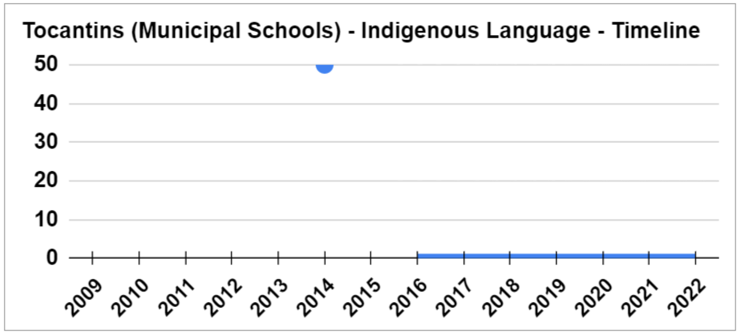

Number of State Schools

	2009	2010	2011	2012	2013	2014	2015	2016	2017	2018	2019	2020	2021	2022	Year
TO	92	95	95	81	80	82	83	84	90	93	93	94	94	98	n

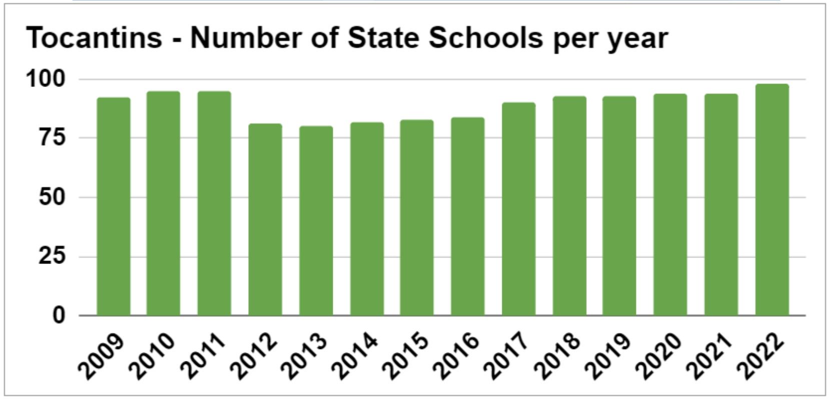

Number of Municipal Schools

	2009	2010	2011	2012	2013	2014	2015	2016	2017	2018	2019	2020	2021	2022	Year
TO						2		2	3	3	3	3	1	3	n

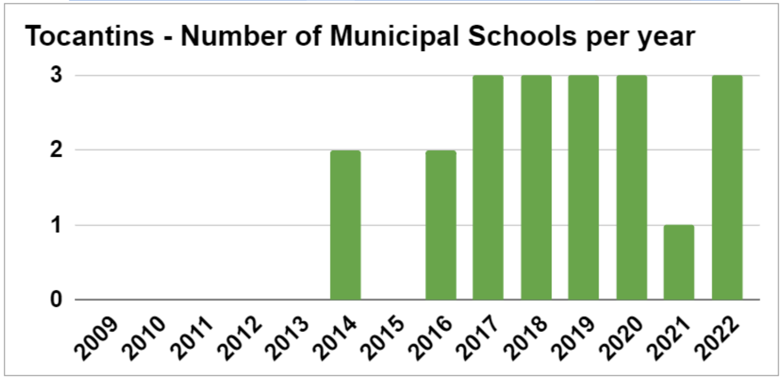

North Region

Figure S08

Histogram - Indigenous Material (Municipal)

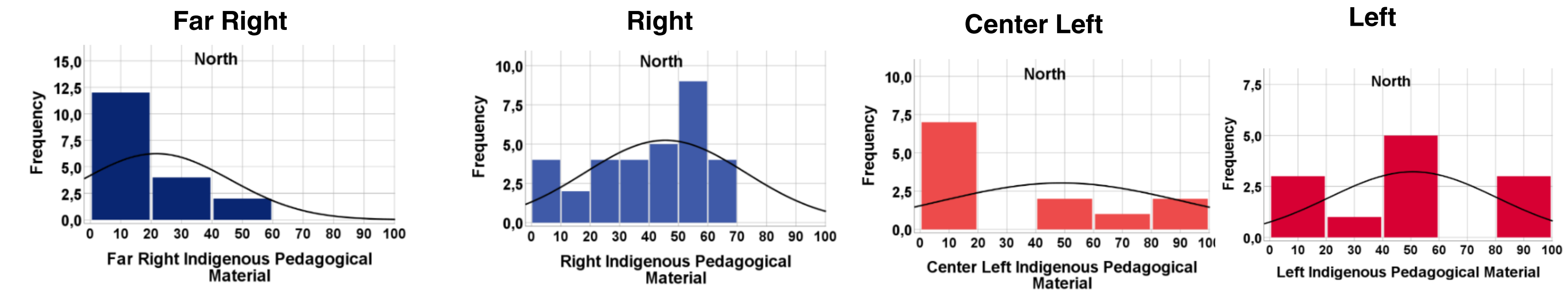

Histogram - Indigenous Material (State)

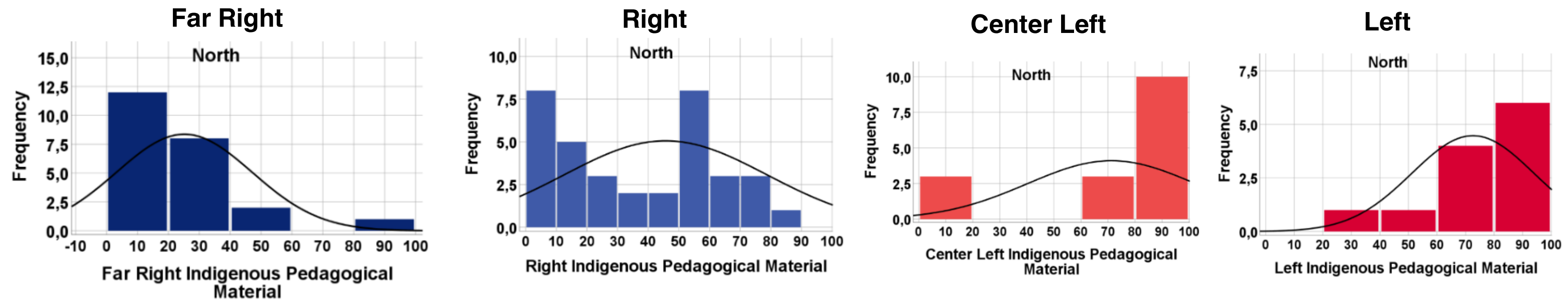

Histogram - Indigenous Language (Municipal)

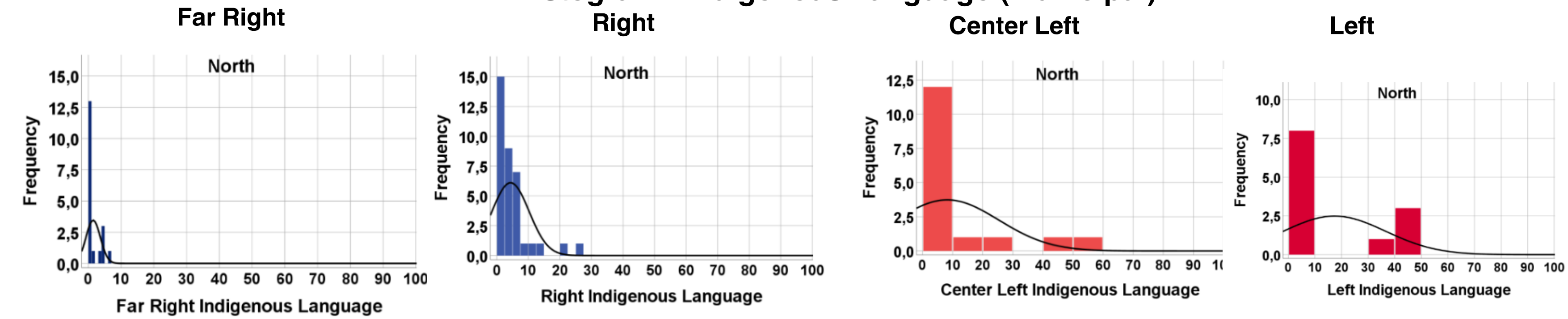

Histogram - Indigenous Language (State)

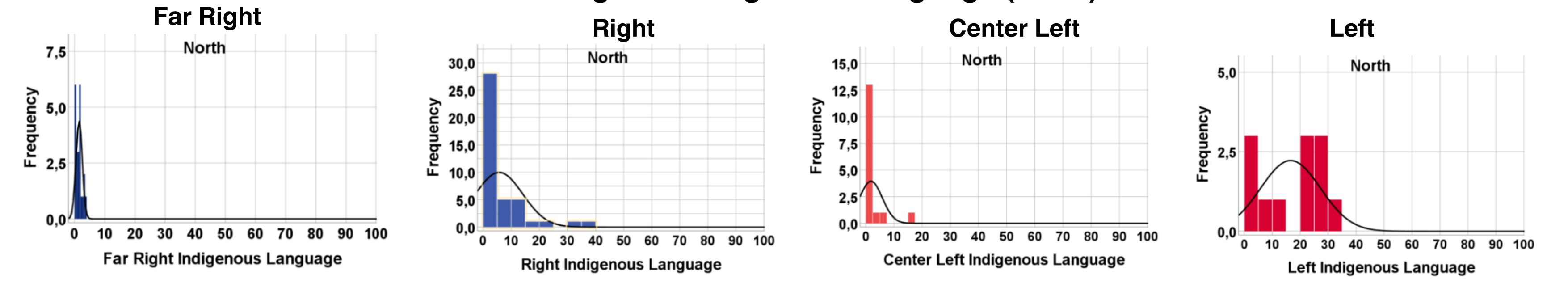

State of Goias

Figure S09

Far Right
Right
Center Right
Center
Center Left
Left

Indigenous Material - State Schools

	2009	2010	2011	2012	2013	2014	2015	2016	2017	2018	2019	2020	2021	2022	Year
GO	50	100	100	50	100	100	100	100	100	66,67	33,33	33,33	33,33	100	%

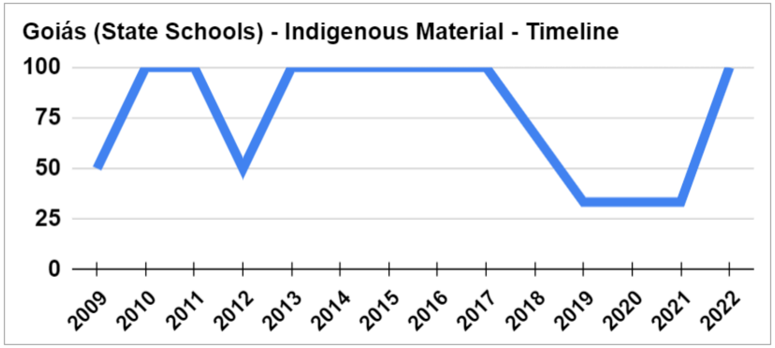

Indigenous Language - State Schools

	2009	2010	2011	2012	2013	2014	2015	2016	2017	2018	2019	2020	2021	2022	Year
GO	50	0	0	0	50	50	50	50	50	33,33	33,33	66,67	33,33	33,33	%

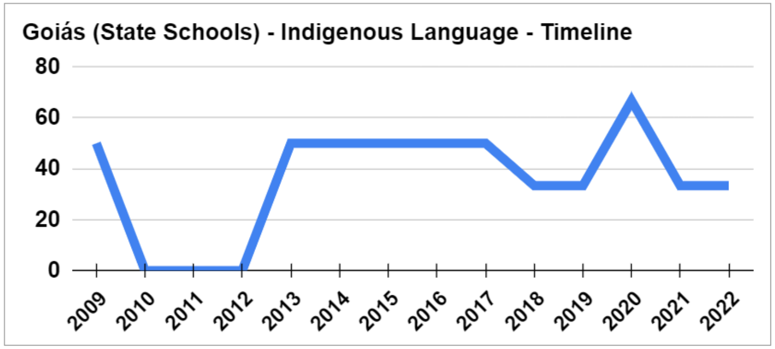

Number of State Schools

	2009	2010	2011	2012	2013	2014	2015	2016	2017	2018	2019	2020	2021	2022	Year
GO	2	2	2	2	2	2	2	2	2	3	3	3	3	3	n

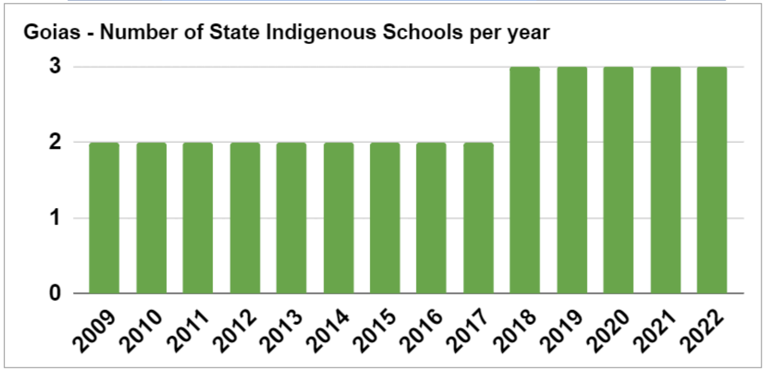

State of Mato Grosso

Figure S10

- Far Right
- Right
- Center Right
- Center
- Center Left
- Left

Indigenous Material - State Schools

	2009	2010	2011	2012	2013	2014	2015	2016	2017	2018	2019	2020	2021	2022	Year
MT	76,36	18,18	31,03	35,48	40	53,23	63,08	59,42	60,87	71,01	57,14	65,71	71,01	75,71	%

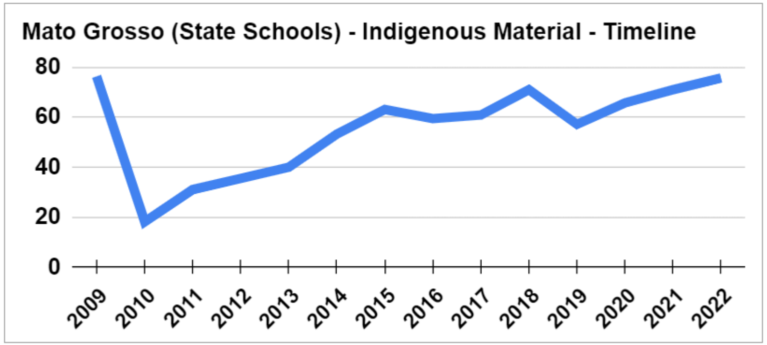

Indigenous Material - Municipal Schools

	2009	2010	2011	2012	2013	2014	2015	2016	2017	2018	2019	2020	2021	2022	Year
MT	76,52	77,44	72,58	60,15	62,88	58,91	55,04	40,15	57,58	65,08	63,33	64,41	63,03	69,7	%

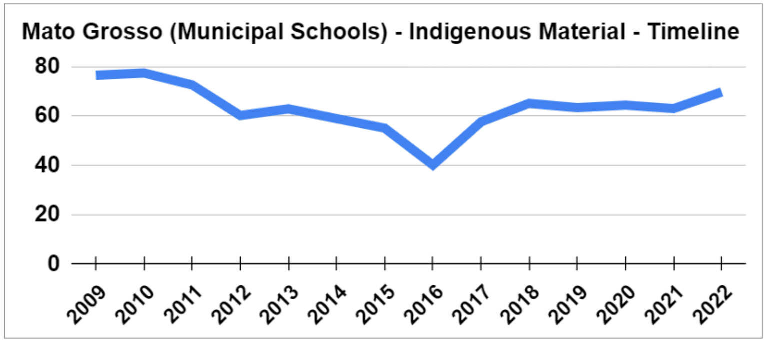

Indigenous Language - State Schools

	2009	2010	2011	2012	2013	2014	2015	2016	2017	2018	2019	2020	2021	2022	Year
MT	7,27	4,55	3,45	1,61	1,54	3,23	3,08	1,45	1,45	4,35	14,29	8,57	2,9	4,29	%

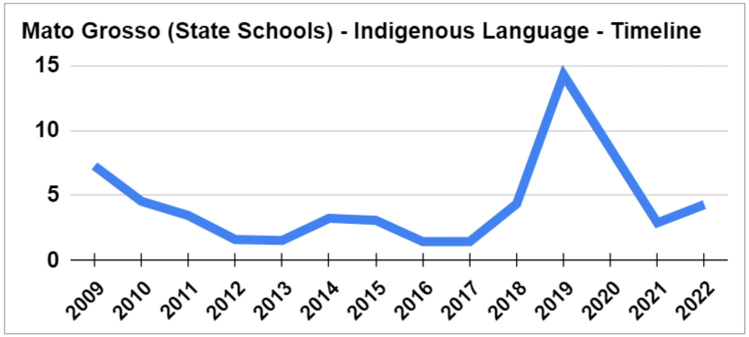

Indigenous Language - Municipal Schools

	2009	2010	2011	2012	2013	2014	2015	2016	2017	2018	2019	2020	2021	2022	Year
MT	7,58	9,02	16,13	6,77	9,09	7,75	5,43	6,82	6,82	8,73	7,5	5,93	10,08	6,06	%

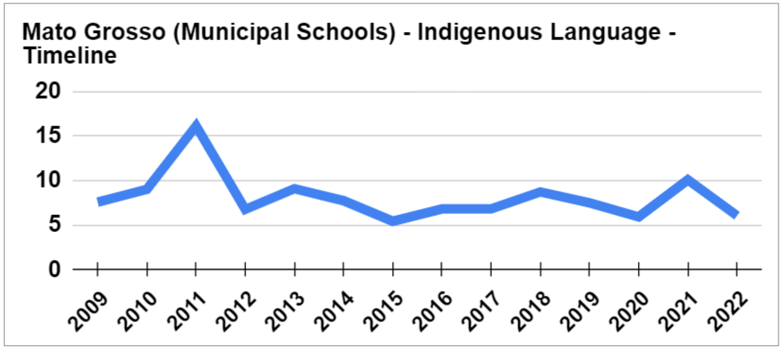

Number of State Schools

	2009	2010	2011	2012	2013	2014	2015	2016	2017	2018	2019	2020	2021	2022	Year
MT	55	44	58	62	65	62	65	69	69	69	70	70	69	70	n

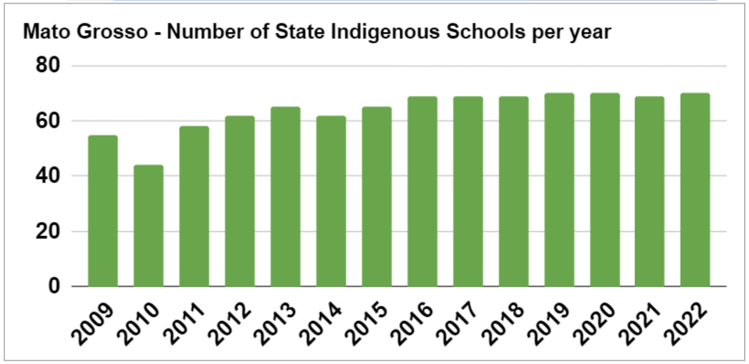

Number of Municipal Schools

	2009	2010	2011	2012	2013	2014	2015	2016	2017	2018	2019	2020	2021	2022	Year
MT	132	133	124	133	132	129	129	132	132	126	120	118	119	132	n

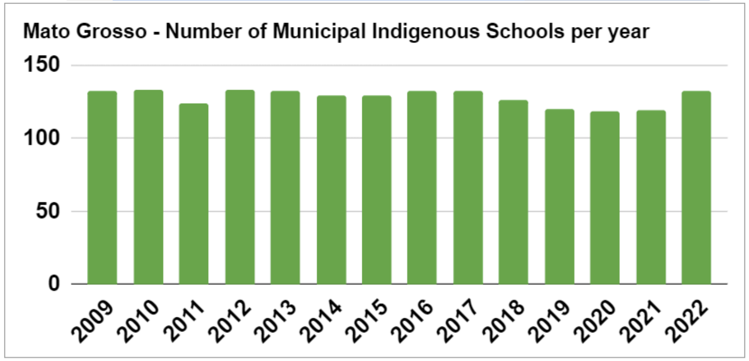

State of Mato Grosso do Sul

Figure S11

Legend
Far Right
Right
Center Right
Center
Center Left
Left

Indigenous Material - State Schools

	2009	2010	2011	2012	2013	2014	2015	2016	2017	2018	2019	2020	2021	2022	Year
MS	50	53,85	42,86	42,86	76,92	53,85	57,14	40	53,33	46,67	33,33	40	29,41	35,29	%

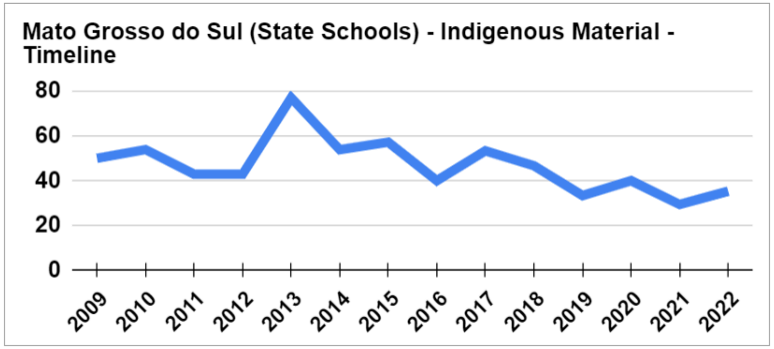

Indigenous Material - Municipal Schools

	2009	2010	2011	2012	2013	2014	2015	2016	2017	2018	2019	2020	2021	2022	Year
MS	58,97	53,85	62,5	68,29	72,97	65,79	71,79	53,66	67,44	64,29	72,09	72,73	76,09	78,72	%

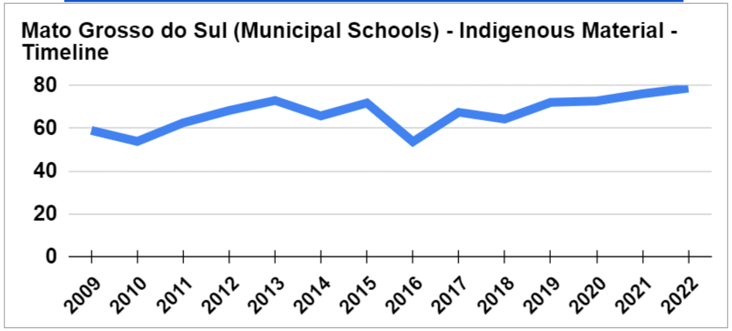

Indigenous Language - State Schools

	2009	2010	2011	2012	2013	2014	2015	2016	2017	2018	2019	2020	2021	2022	Year
MS	0	7,69	0	0	7,69	23,08	14,29	13,33	6,67	6,67	6,67	6,67	0	0	%

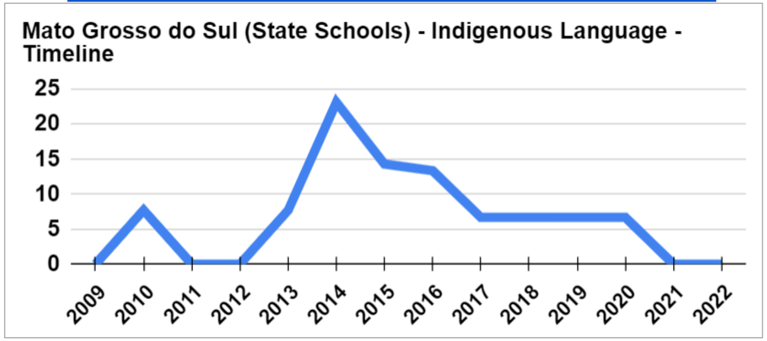

Indigenous Language - Municipal Schools

	2009	2010	2011	2012	2013	2014	2015	2016	2017	2018	2019	2020	2021	2022	Year
MS	10,26	12,82	0	14,63	13,51	0	2,56	2,44	2,33	4,76	2,33	2,27	2,17	2,13	%

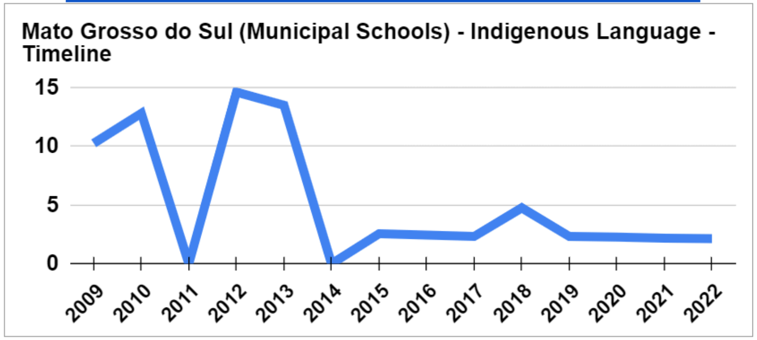

Number of State Schools

	2009	2010	2011	2012	2013	2014	2015	2016	2017	2018	2019	2020	2021	2022	Year
MS	12	13	14	14	13	13	14	15	15	15	15	15	17	17	n

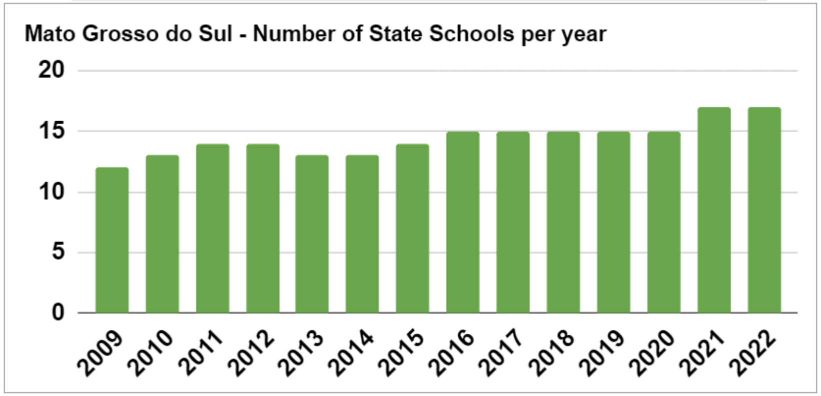

Number of Municipal Schools

	2009	2010	2011	2012	2013	2014	2015	2016	2017	2018	2019	2020	2021	2022	Year
MS	39	39	40	41	37	38	39	41	43	42	43	44	46	47	n

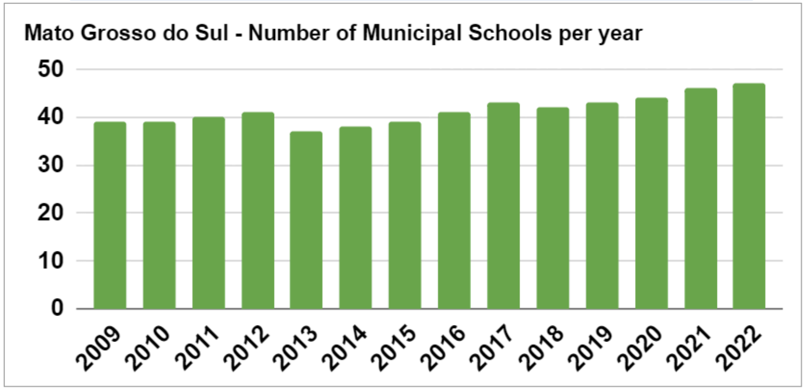

Mid West Region

Figure S12

Histogram - Indigenous Material (Municipal)

Far Right

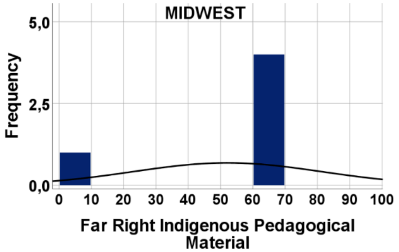

Right

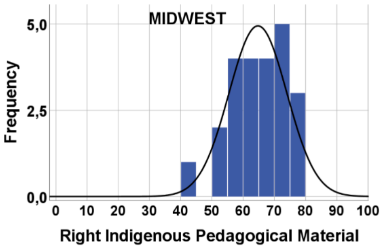

Histogram - Indigenous Material (State)

Far Right

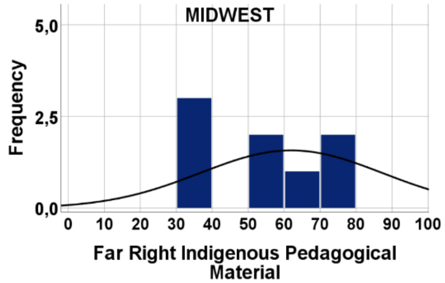

Right

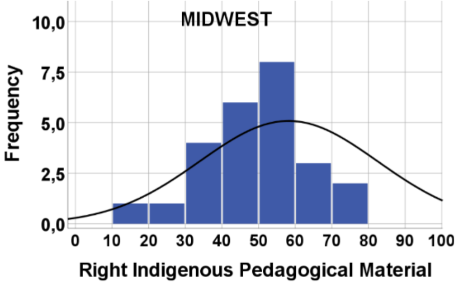

Histogram - Indigenous Language (Municipal)

Far Right

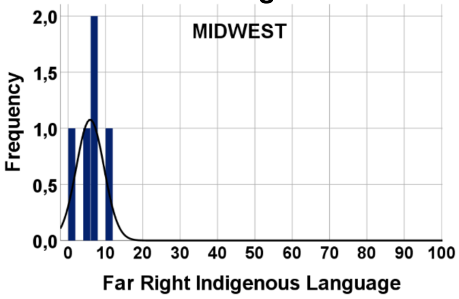

Right

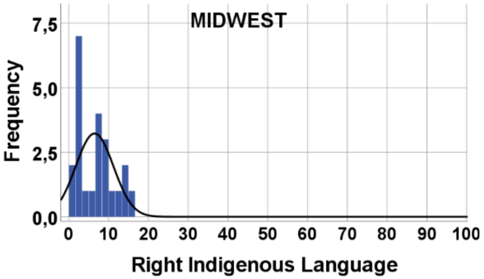

Histogram - Indigenous Language (State)

Far Right

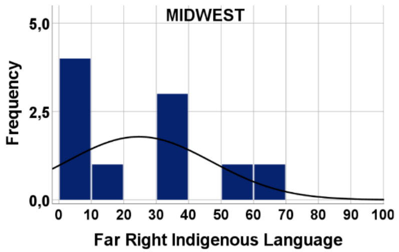

Right

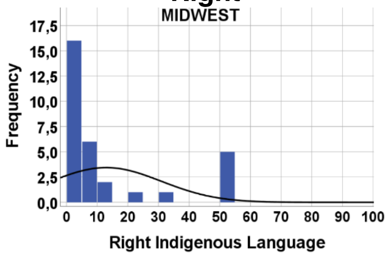

State of Paraná

Figure S13

- Far Right
- Right
- Center Right
- Center
- Center Left
- Left

Indigenous Material - State Schools

	2009	2010	2011	2012	2013	2014	2015	2016	2017	2018	2019	2020	2021	2022	Year
PR	72,73	73,53	75	80	91,67	94,59	97,14	94,59	92,11	94,74	81,58	87,18	86,11	89,47	%

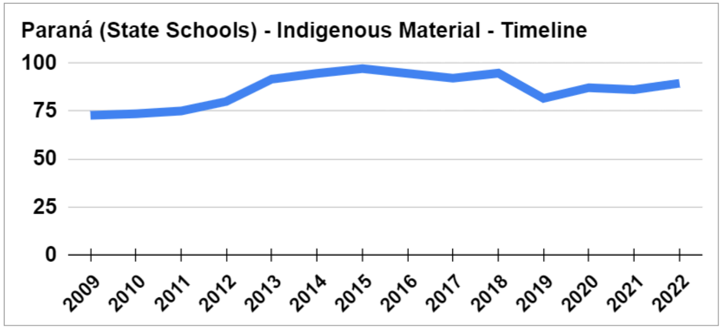

Indigenous Material - Municipal Schools

	2009	2010	2011	2012	2013	2014	2015	2016	2017	2018	2019	2020	2021	2022	Year
PR										0					%

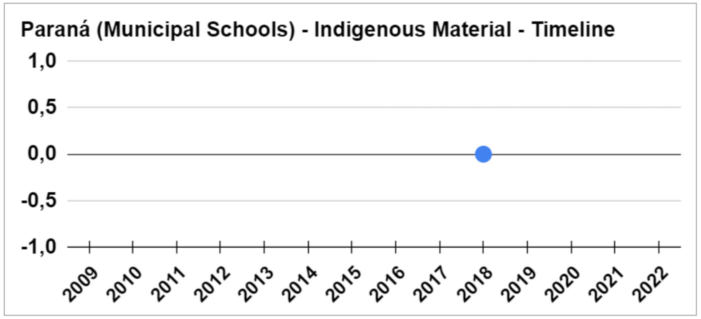

Indigenous Language - State Schools

	2009	2010	2011	2012	2013	2014	2015	2016	2017	2018	2019	2020	2021	2022	Year
PR	15,15	52,94	80,56	100	97,22	94,59	97,14	94,59	97,37	89,47	89,47	94,87	94,44	89,47	%

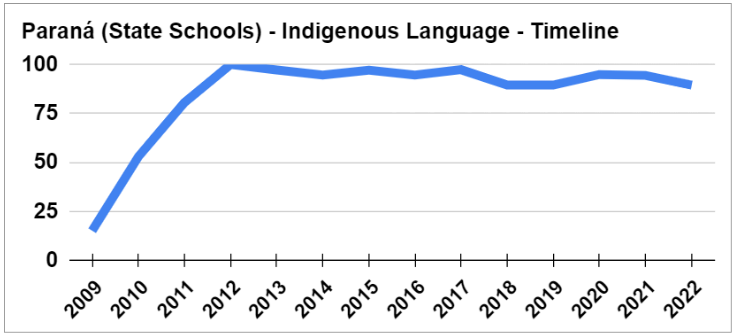

Indigenous Language - Municipal Schools

	2009	2010	2011	2012	2013	2014	2015	2016	2017	2018	2019	2020	2021	2022	Year
PR										100					%

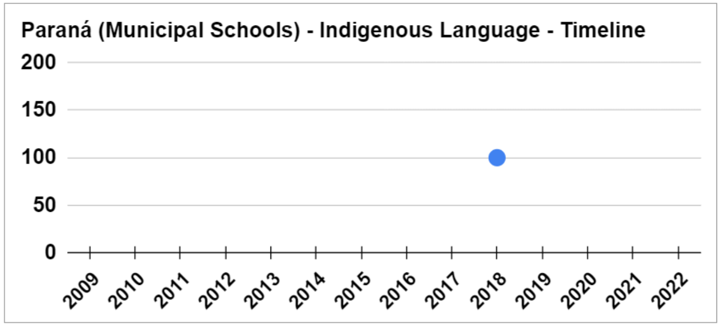

Number of State Schools

	2009	2010	2011	2012	2013	2014	2015	2016	2017	2018	2019	2020	2021	2022	Year
PR	33	34	36	35	36	37	35	37	38	38	38	39	36	38	n

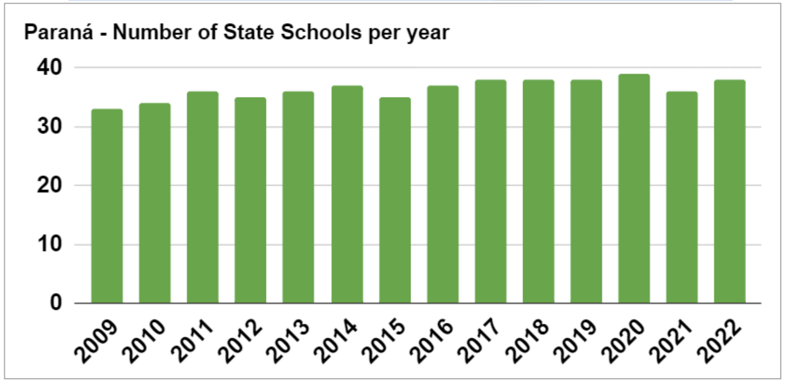

Number of Municipal Schools

	2009	2010	2011	2012	2013	2014	2015	2016	2017	2018	2019	2020	2021	2022	Year
PR										1					n

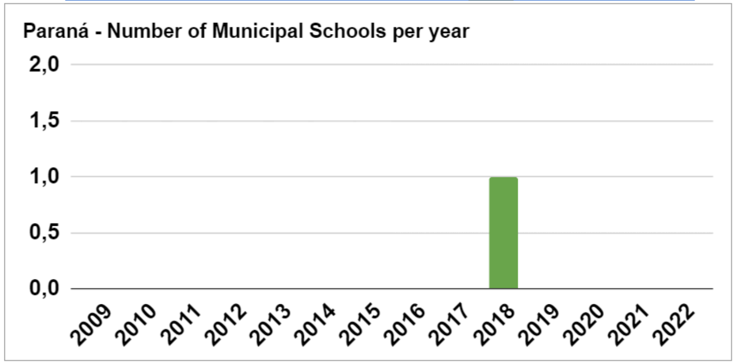

State of Santa Catarina

Figure S14

Colour	Category
	Far Right
	Right
	Center Right
	Center
	Center Left
	Left

Indigenous Material - State Schools

	2009	2010	2011	2012	2013	2014	2015	2016	2017	2018	2019	2020	2021	2022	Year
SC	90,91	78,79	82,35	87,5	96,77	83,33	93,33	93,75	87,1	83,33	76,67	70	68,75	87,5	%

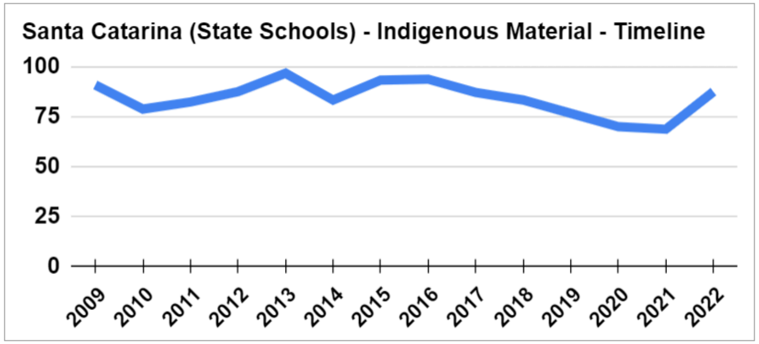

Indigenous Material - Municipal Schools

	2009	2010	2011	2012	2013	2014	2015	2016	2017	2018	2019	2020	2021	2022	Year
SC	60	33,33	66,67	66,67	100	100	80	42,86	71,43	28,57	28,57	28,57	14,29	28,57	%

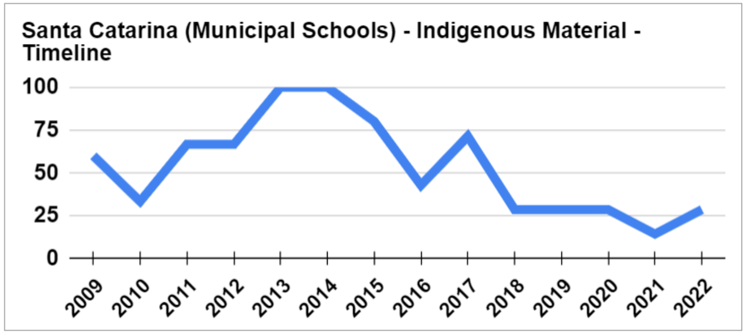

Indigenous Language - State Schools

	2009	2010	2011	2012	2013	2014	2015	2016	2017	2018	2019	2020	2021	2022	Year
SC	15,15	3,03	11,76	3,13	3,23	0	3,33	3,13	6,45	10	3,33	3,33	6,25	6,25	%

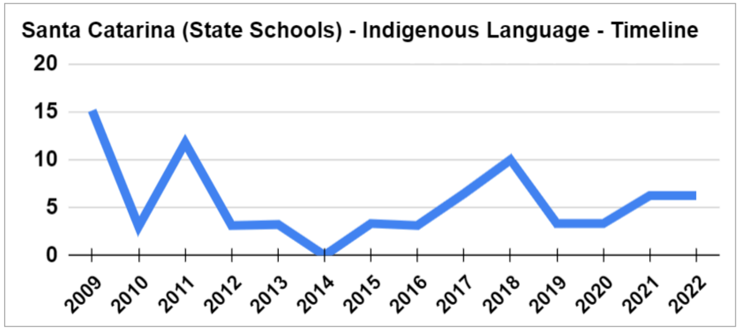

Indigenous Language - Municipal Schools

	2009	2010	2011	2012	2013	2014	2015	2016	2017	2018	2019	2020	2021	2022	Year
SC	0	16,67	16,67	50	0	0	0	0	0	0	0	0	14,29	14,29	%

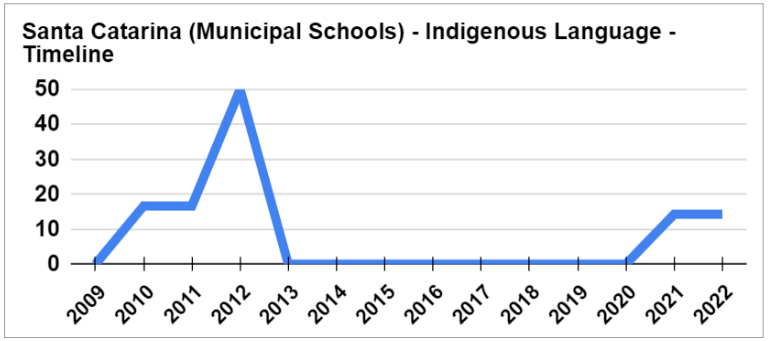

Number of State Schools

	2009	2010	2011	2012	2013	2014	2015	2016	2017	2018	2019	2020	2021	2022	Year
SC	33	33	34	32	31	30	30	32	31	30	30	30	32	32	n

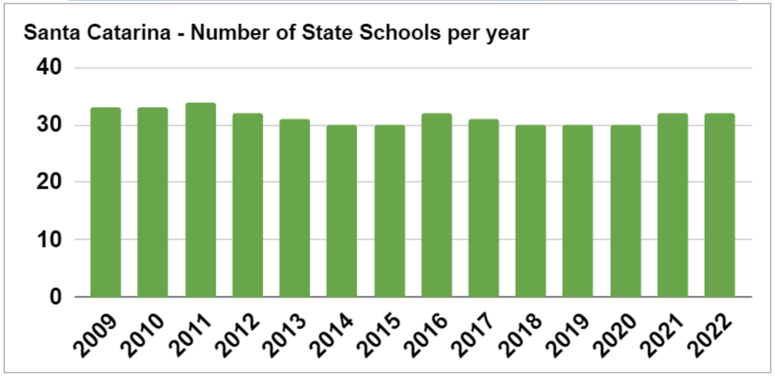

Number of Municipal Schools

	2009	2010	2011	2012	2013	2014	2015	2016	2017	2018	2019	2020	2021	2022	Year
SC	5	6	6	6	6	6	5	7	7	7	7	7	7	7	n

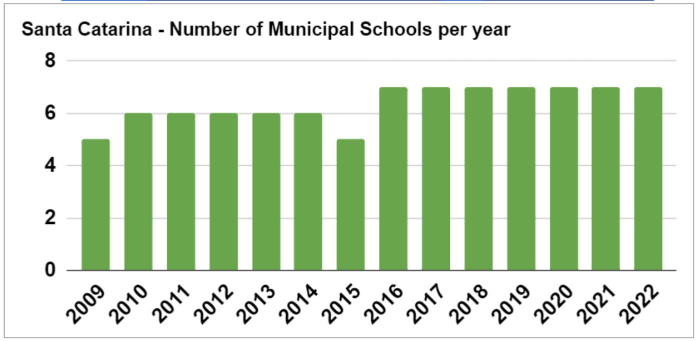

State of Rio Grande do Sul

Figure S15

Legend
Far Right
Right
Center Right
Center
Center Left
Left

Indigenous Material - State Schools

	2009	2010	2011	2012	2013	2014	2015	2016	2017	2018	2019	2020	2021	2022	Year
RS	87,76	95,74	95,16	95,24	86,49	93,9	92,68	86,75	88,1	38,67	21,18	22,08	32,91	32,91	%

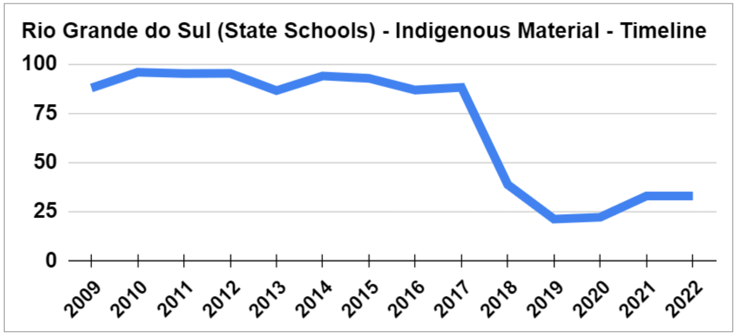

Indigenous Material - Municipal Schools

	2009	2010	2011	2012	2013	2014	2015	2016	2017	2018	2019	2020	2021	2022	Year
RS	50	80	60	50	17	33,33	33,33	16,67	50	50	16,67	42,86	57,14	57,14	%

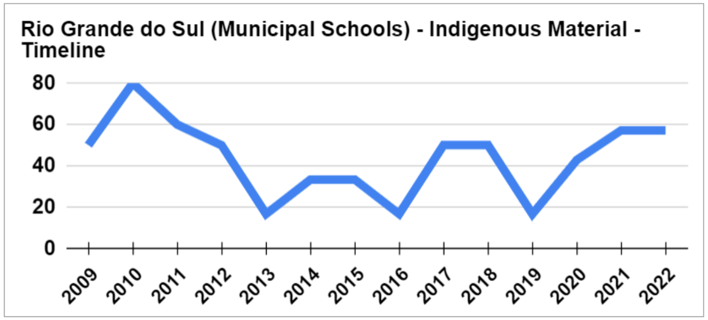

Indigenous Language - State Schools

	2009	2010	2011	2012	2013	2014	2015	2016	2017	2018	2019	2020	2021	2022	Year
RS	12,24	14,89	17,74	17,46	2,70	2,44	21,95	10,84	8,33	14,67	9,41	7,79	6,33	7,59	%

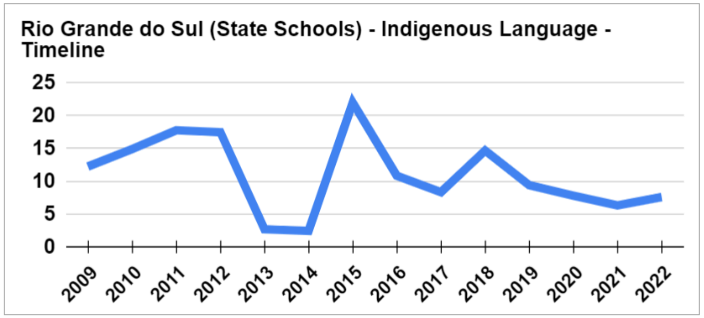

Indigenous Language - Municipal Schools

	2009	2010	2011	2012	2013	2014	2015	2016	2017	2018	2019	2020	2021	2022	Year
RS	25,00	20,00	20,00	0,00	33,33	16,67	16,67	16,67	16,67	16,67	16,67	14,29	14,29	14,29	%

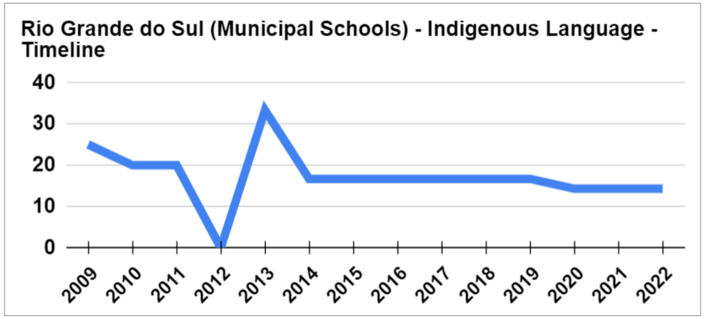

Number of State Schools

	2009	2010	2011	2012	2013	2014	2015	2016	2017	2018	2019	2020	2021	2022	Year
RS	49	47	62	63	74	82	82	83	84	75	85	77	79	79	n

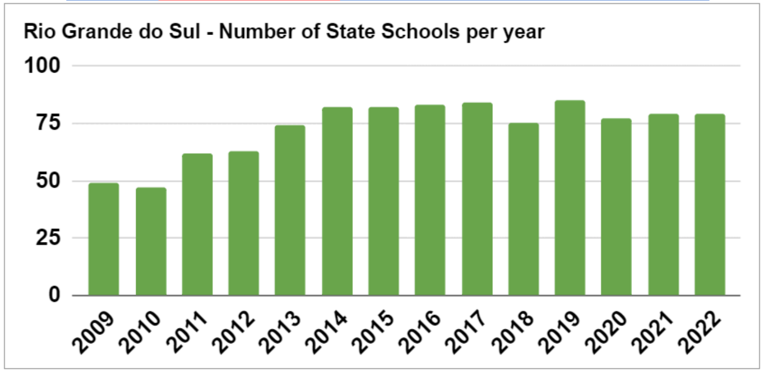

Number of Municipal Schools

	2009	2010	2011	2012	2013	2014	2015	2016	2017	2018	2019	2020	2021	2022	Year
RS	4	5	5	6	6	6	6	6	6	6	6	7	7	7	n

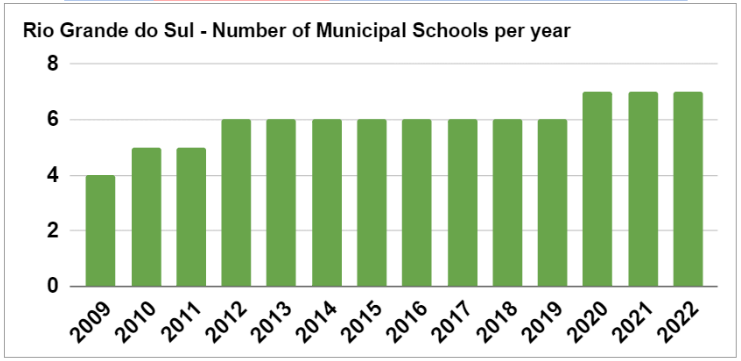

South Region

Figure S16

Histogram - Indigenous Material (Municipal)

Far Right

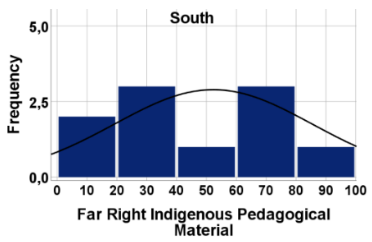

Right

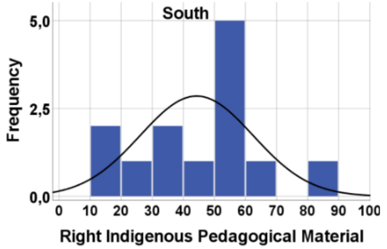

Left

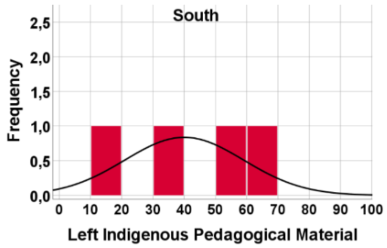

Histogram - Indigenous Material (State)

Far Right

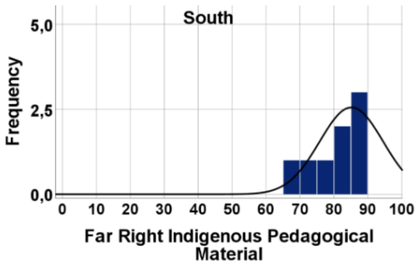

Right

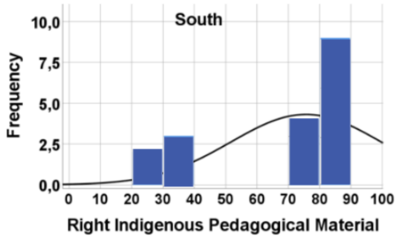

Left

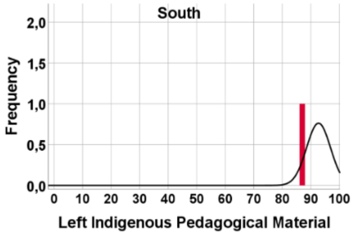

Histogram - Indigenous Language (Municipal)

Far Right

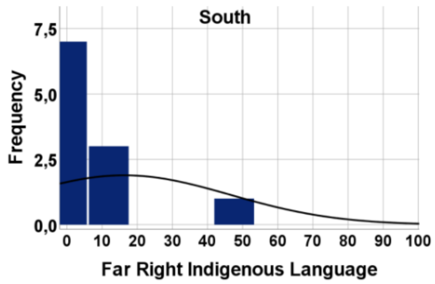

Right

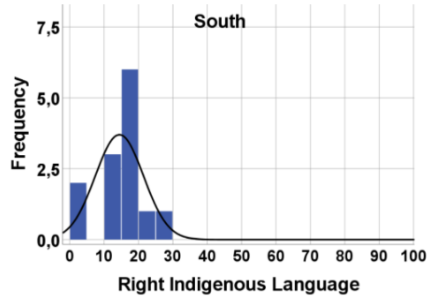

Left

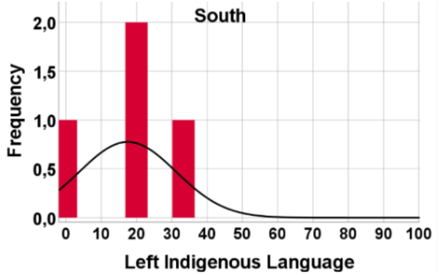

Histogram - Indigenous Language (State)

Far Right

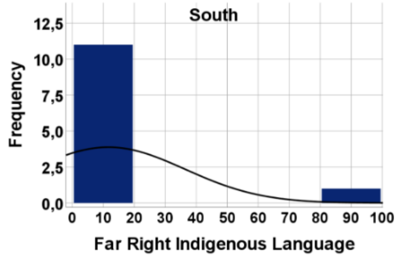

Right

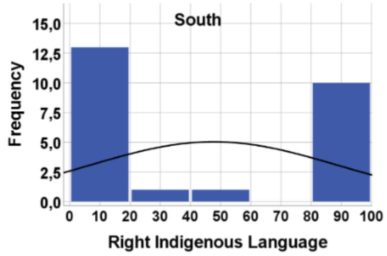

Left

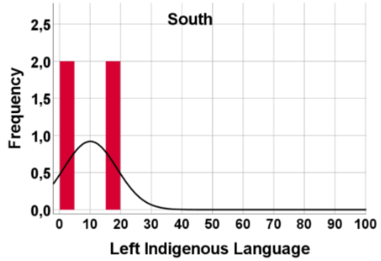

State of Minas Gerais

Figure S17

	Far Right
	Right
	Center Right
	Center
	Center Left
	Left

Indigenous Material - State Schools

	2009	2010	2011	2012	2013	2014	2015	2016	2017	2018	2019	2020	2021	2022	Year
MG	80	93,33	86,67	93,33	100	100	100	100	100	95	47,37	52,63	52,63	55	%

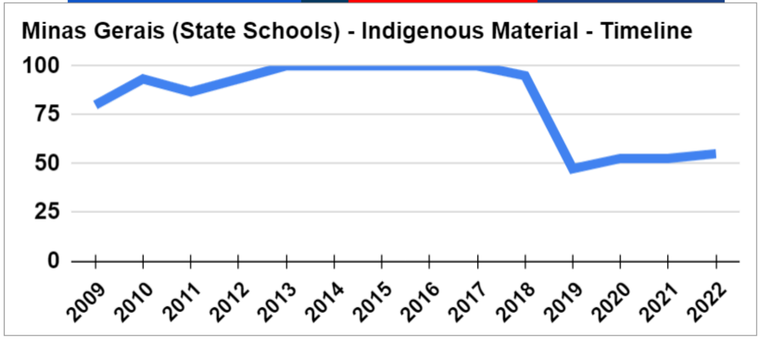

Indigenous Language - State Schools

	2009	2010	2011	2012	2013	2014	2015	2016	2017	2018	2019	2020	2021	2022	Year
MG	0	20	20	20	26,67	25	23,53	23,53	23,53	20	15,79	21,05	21,05	30	%

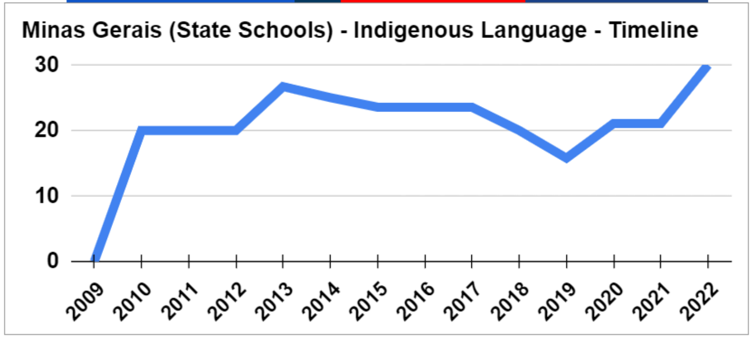

Number of State Schools

	2009	2010	2011	2012	2013	2014	2015	2016	2017	2018	2019	2020	2021	2022	Year
MG	15	15	15	15	15	16	17	17	17	20	19	19	19	20	n

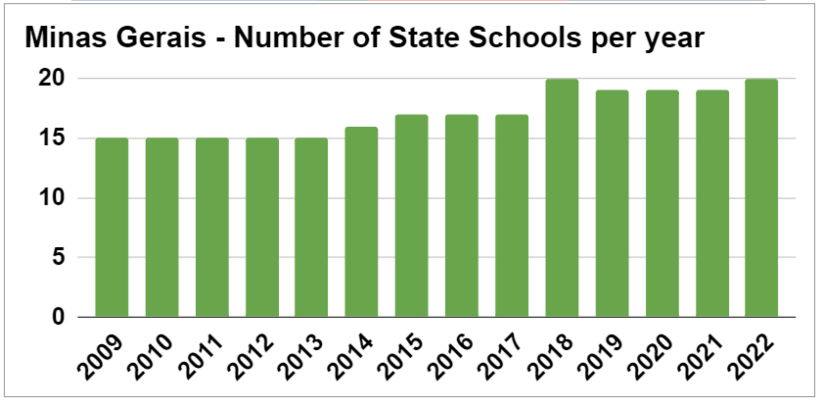

State of São Paulo

Figure S18

- Far Right
- Right
- Center Right
- Center
- Center Left
- Left

Indigenous Material - State Schools

	2009	2010	2011	2012	2013	2014	2015	2016	2017	2018	2019	2020	2021	2022	Year
SP	100	96,77	61,29	8,82	11,76	0	95,83	100	100	100	25	51,28	40,74	52,63	%

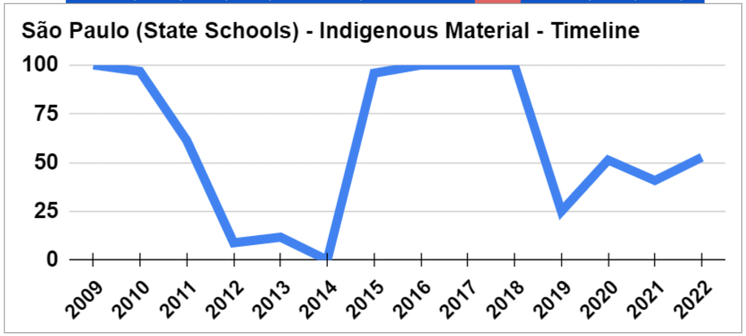

Indigenous Material - Municipal Schools

	2009	2010	2011	2012	2013	2014	2015	2016	2017	2018	2019	2020	2021	2022	Year
SP	100	100	75	75	75	75	100	75	75	100	50	25	50	50	%

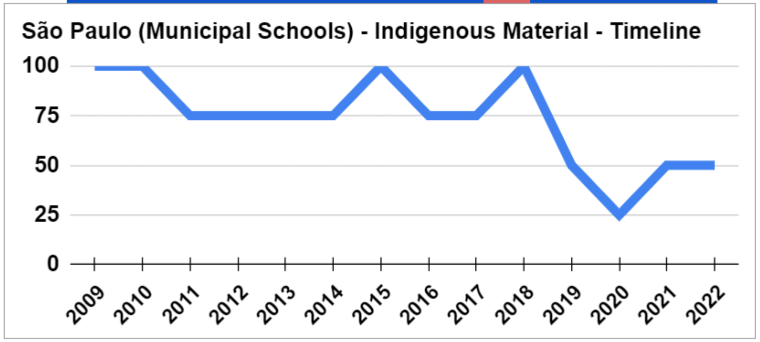

Indigenous Language - State Schools

	2009	2010	2011	2012	2013	2014	2015	2016	2017	2018	2019	2020	2021	2022	Year
SP	0	3,23	0	88,24	55,88	79,49	83,33	76,92	0	30	15	12,82	14,81	13,16	%

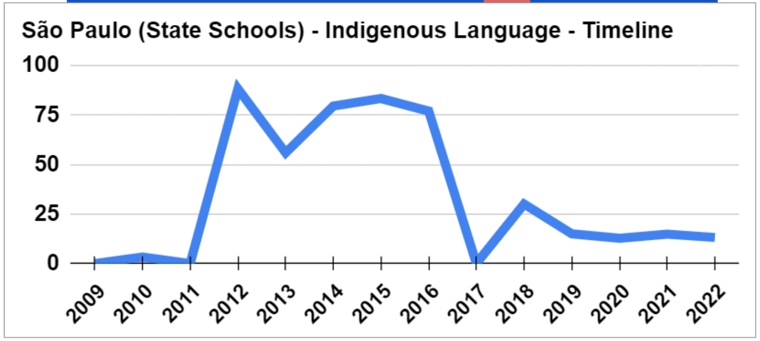

Indigenous Language - Municipal Schools

	2009	2010	2011	2012	2013	2014	2015	2016	2017	2018	2019	2020	2021	2022	Year
SP	75	75	75	75	75	75	75	75	75	100	75	100	75	100	%

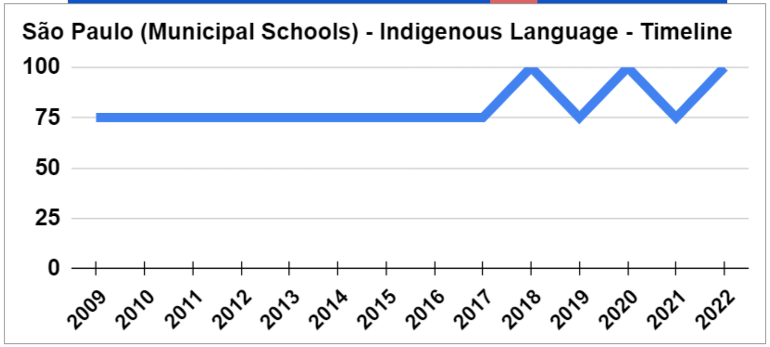

Number of State Schools

	2009	2010	2011	2012	2013	2014	2015	2016	2017	2018	2019	2020	2021	2022	Year
SP	30	31	31	34	34	39	24	13	8	10	40	39	27	38	n

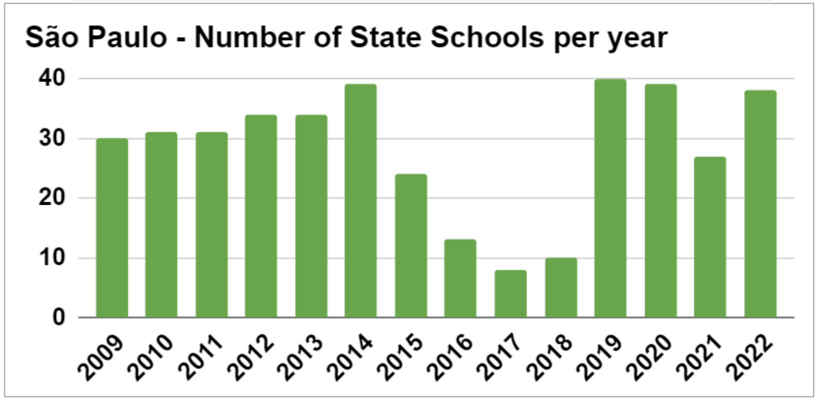

Number of Municipal Schools

	2009	2010	2011	2012	2013	2014	2015	2016	2017	2018	2019	2020	2021	2022	Year
SP	4	4	4	4	4	4	4	4	4	4	4	4	4	4	n

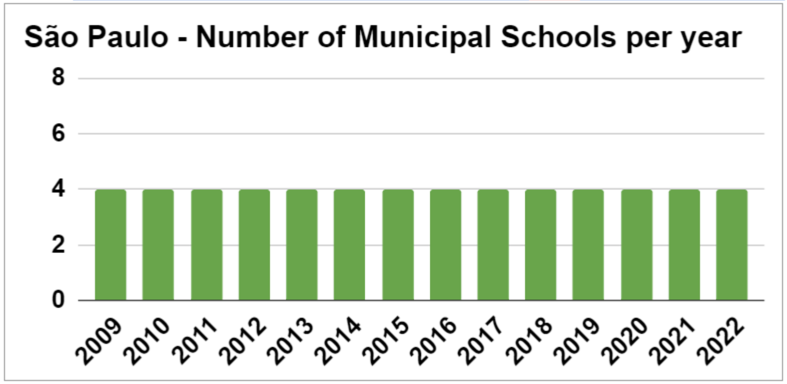

State of Rio de Janeiro

Figure S19

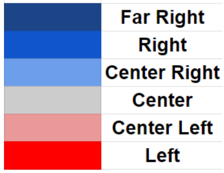

Indigenous Material - State Schools

	2009	2010	2011	2012	2013	2014	2015	2016	2017	2018	2019	2020	2021	2022	Year
RJ	100	100	100	100	100	100		0	0		100	100	100	100	%

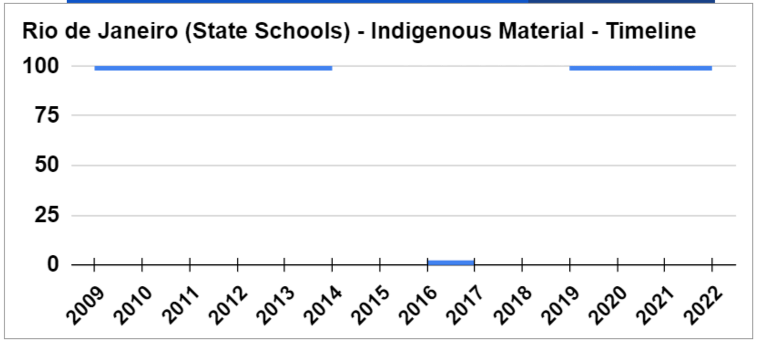

Indigenous Material - Municipal Schools

	2009	2010	2011	2012	2013	2014	2015	2016	2017	2018	2019	2020	2021	2022	Year
RJ								100	100	50	0				%

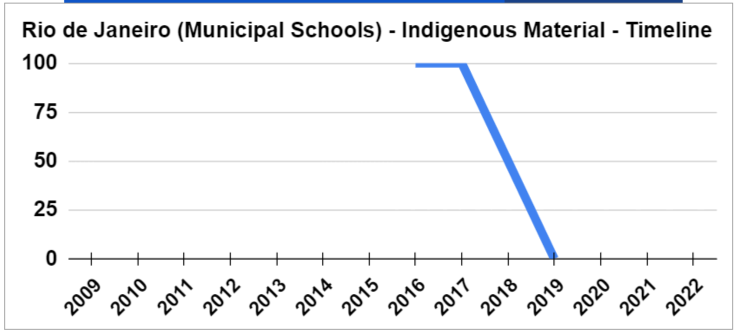

Indigenous Language - State Schools

	2009	2010	2011	2012	2013	2014	2015	2016	2017	2018	2019	2020	2021	2022	Year
RJ	0	0	0	0	0	100		0	0		0	0	0	0	%

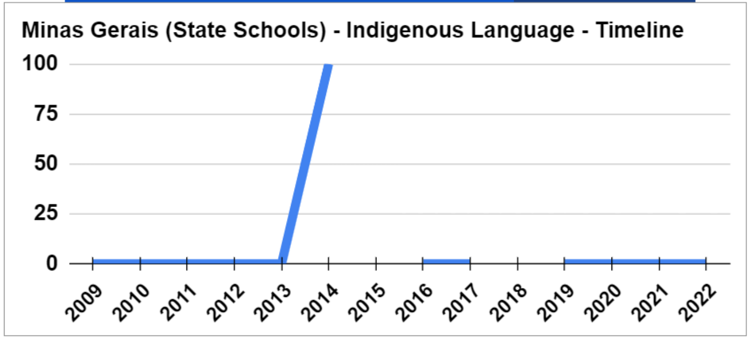

Indigenous Language - Municipal Schools

	2009	2010	2011	2012	2013	2014	2015	2016	2017	2018	2019	2020	2021	2022	Year
RJ								0	0	0	0				%

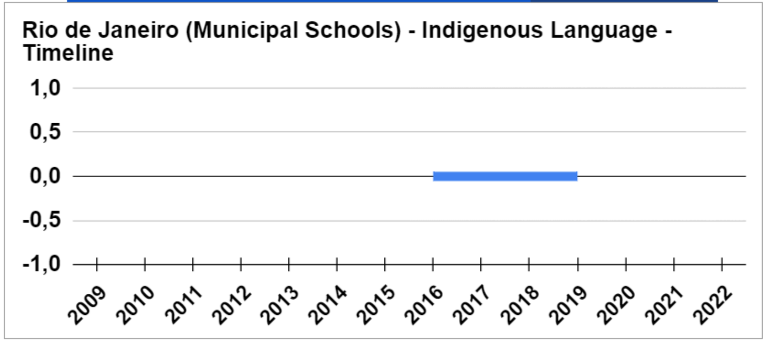

Number of State Schools

	2009	2010	2011	2012	2013	2014	2015	2016	2017	2018	2019	2020	2021	2022	Year
RJ	3	3	3	3	1	1		1	1		1	1	1	1	n

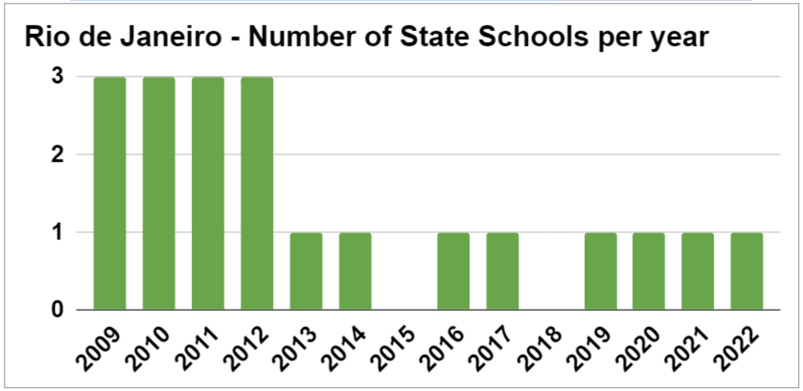

Number of Municipal Schools

	2009	2010	2011	2012	2013	2014	2015	2016	2017	2018	2019	2020	2021	2022	Year
RJ								2	2	2	1				n

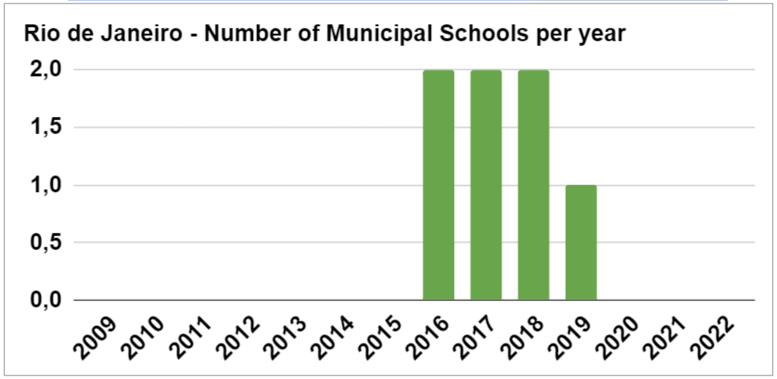

State of Espirito Santo

Figure S20

Far Right
Right
Center Right
Center
Center Left
Left

Indigenous Material - State Schools

	2009	2010	2011	2012	2013	2014	2015	2016	2017	2018	2019	2020	2021	2022	Year
ES													0	0	%

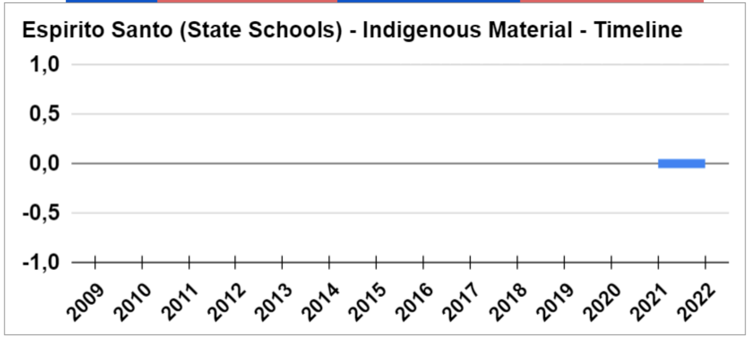

Indigenous Material - Municipal Schools

	2009	2010	2011	2012	2013	2014	2015	2016	2017	2018	2019	2020	2021	2022	Year
ES	14,29	42,86	28,57	85,71	85,71	57,14	14,29	14,29	83,33	83,33	100	100	83,33	83,33	%

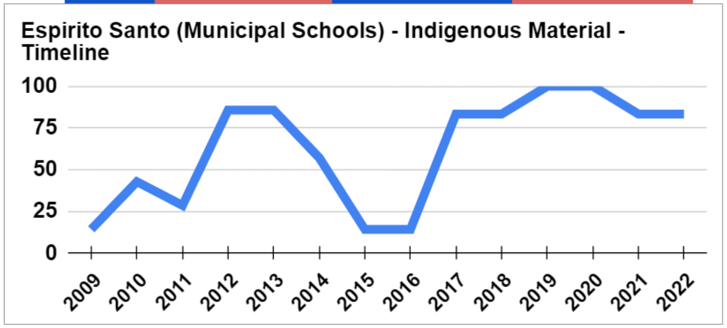

Indigenous Language - State Schools

	2009	2010	2011	2012	2013	2014	2015	2016	2017	2018	2019	2020	2021	2022	Year
ES													100	0	%

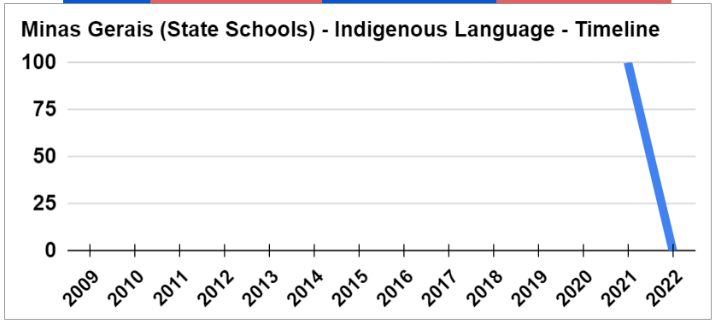

Indigenous Language - Municipal Schools

	2009	2010	2011	2012	2013	2014	2015	2016	2017	2018	2019	2020	2021	2022	Year
ES	0	28,57	14,29	0	0	0	0	0	0	0	0	0	0	0	%

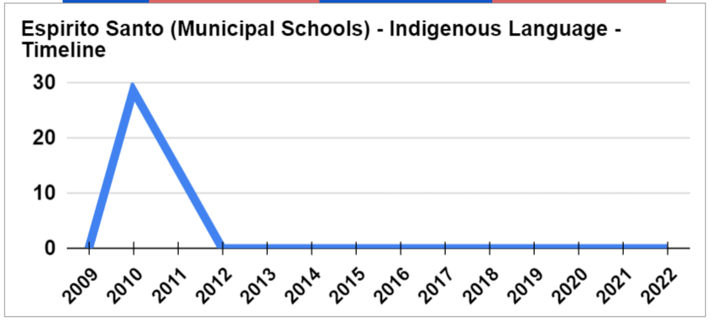

Number of State Schools

	2009	2010	2011	2012	2013	2014	2015	2016	2017	2018	2019	2020	2021	2022	Year
ES													1	1	n

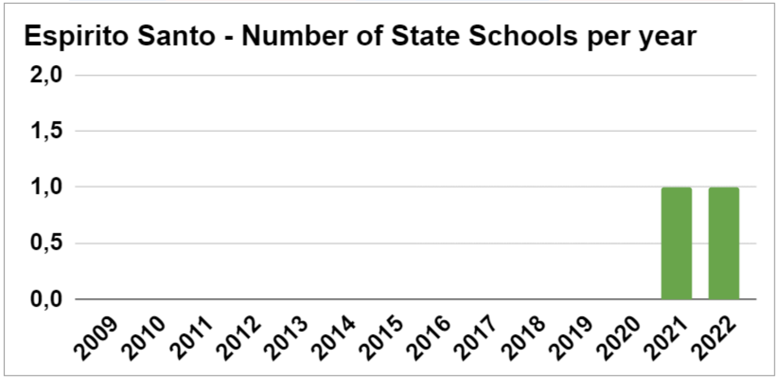

Number of Municipal Schools

	2009	2010	2011	2012	2013	2014	2015	2016	2017	2018	2019	2020	2021	2022	Year
ES	32	32	32	32	32	32	32	32	32	32	32	32	32	32	n

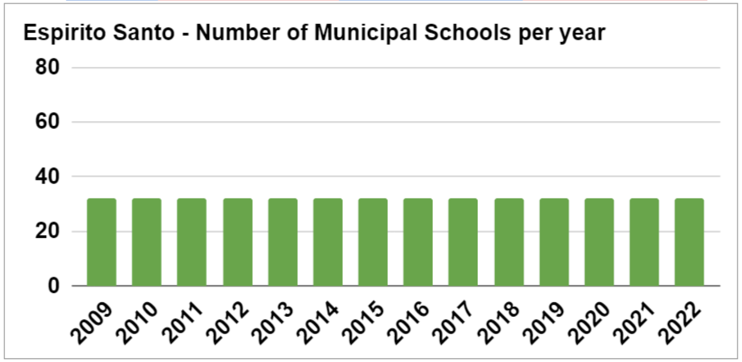

Southeast Region

Histogram - Indigenous Material (Municipal)

Right

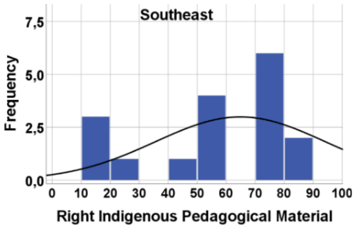

Center Left

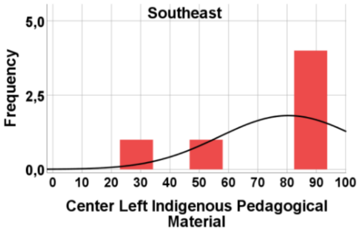

Histogram - Indigenous Material (State)

Far Right

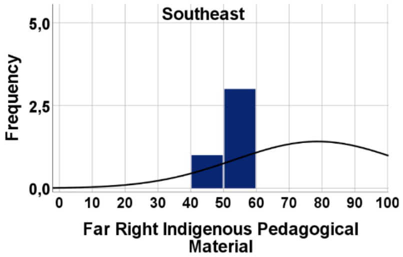

Right

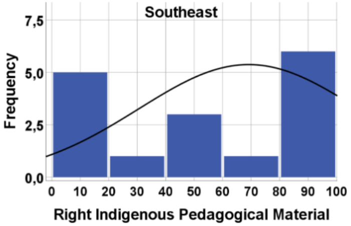

Histogram - Indigenous Language (Municipal)

Right

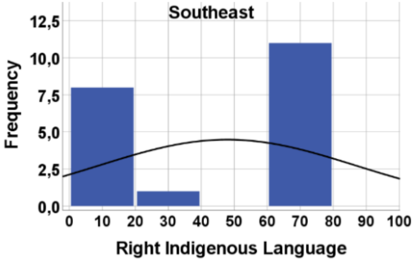

Center Left

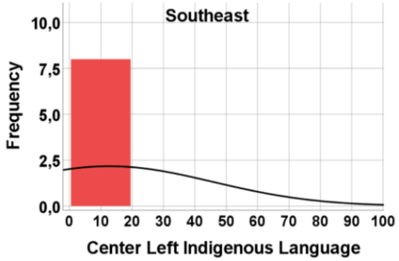

Histogram - Indigenous Language (State)

Far Right

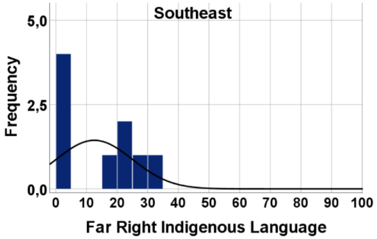

Right

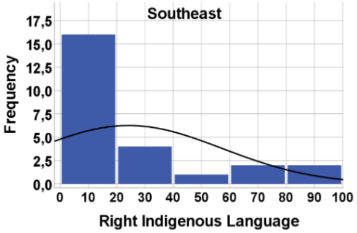

State of Ceará

Figure S22

Legend
Far Right
Right
Center Right
Center
Center Left
Left

Indigenous Material - State Schools

	2009	2010	2011	2012	2013	2014	2015	2016	2017	2018	2019	2020	2021	2022	Year
CE	84,85	57,14	57,14	61,76	54,55	60,61	55,88	42,86	41,67	59,46	74,36	74,36	76,92	76,92	%

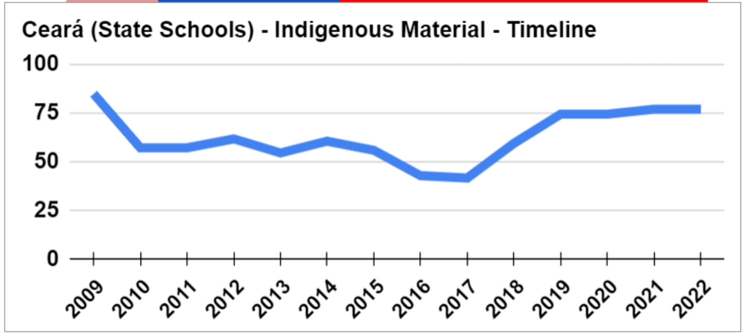

Indigenous Material - Municipal Schools

	2009	2010	2011	2012	2013	2014	2015	2016	2017	2018	2019	2020	2021	2022	Year
CE	50	25	25	50	25	50	100	60	40	0	60	40	40	40	%

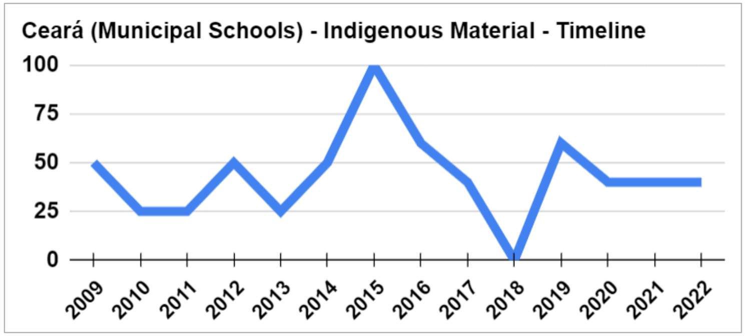

Indigenous Language - State Schools

	2009	2010	2011	2012	2013	2014	2015	2016	2017	2018	2019	2020	2021	2022	Year
CE	0	0	0	0	0	0	0	0	0	0	0	0	0	0	%

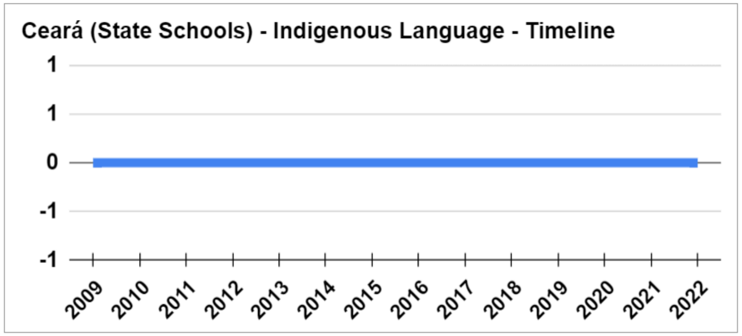

Indigenous Language - Municipal Schools

	2009	2010	2011	2012	2013	2014	2015	2016	2017	2018	2019	2020	2021	2022	Year
CE	0	0	0	0	0	0	0	0	0	0	0	0	0	0	%

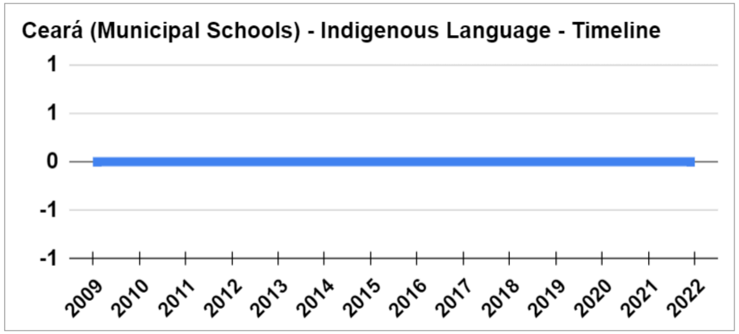

Number of State Schools

	2009	2010	2011	2012	2013	2014	2015	2016	2017	2018	2019	2020	2021	2022	Year
CE	33	35	35	34	33	33	34	35	36	37	39	39	39	39	n

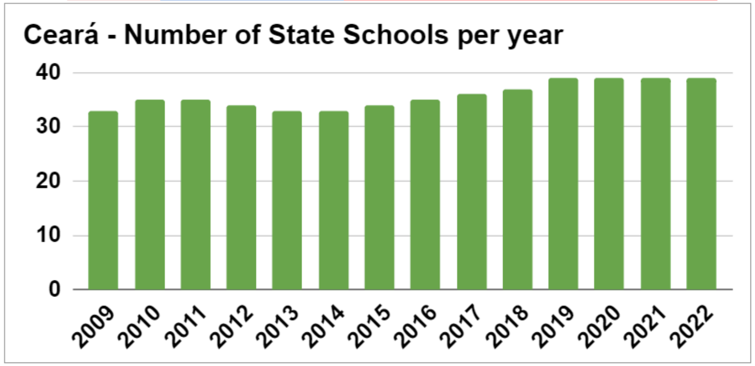

Number of Municipal Schools

	2009	2010	2011	2012	2013	2014	2015	2016	2017	2018	2019	2020	2021	2022	Year
CE	4	4	4	4	4	4	4	5	5	5	5	5	5	5	n

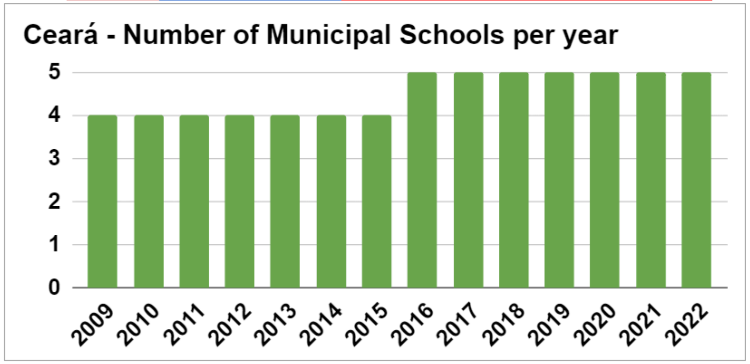

State of Maranhão

Figure S23

Legend
Far Right
Right
Center Right
Center
Center Left
Left

Indigenous Material - State Schools

	2009	2010	2011	2012	2013	2014	2015	2016	2017	2018	2019	2020	2021	2022	Year
MA	31,47	34,53	54,42	69,96	63,7	65,54	62,9	95,02	88,38	93,38	21,98	41,57	41,35	41,83	%

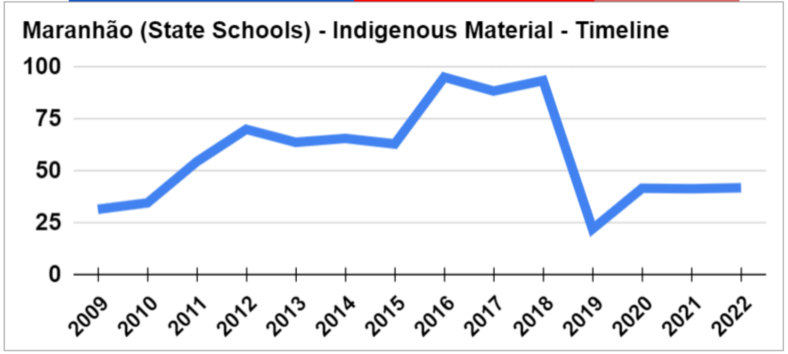

Indigenous Material - Municipal Schools

	2009	2010	2011	2012	2013	2014	2015	2016	2017	2018	2019	2020	2021	2022	Year
MA	8	20	20	25,71	36,36	32	10	33,33	37,5	12,7	9,52	33,33	32,88	35,21	%

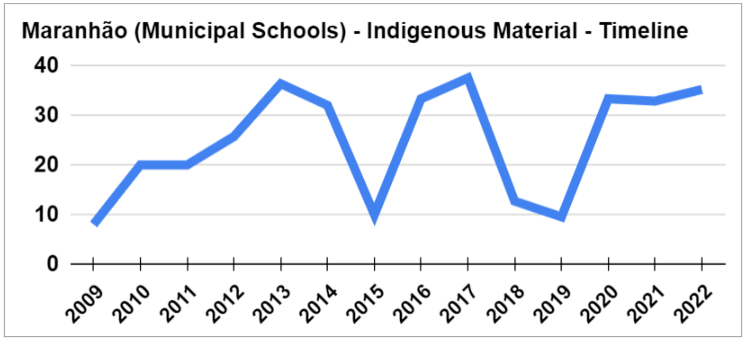

Indigenous Language - State Schools

	2009	2010	2011	2012	2013	2014	2015	2016	2017	2018	2019	2020	2021	2022	Year
MA	4,78	0,36	0,71	10,27	2,85	2,85	1,06	3,2	2,46	2,21	2,2	2,25	1,5	1,52	%

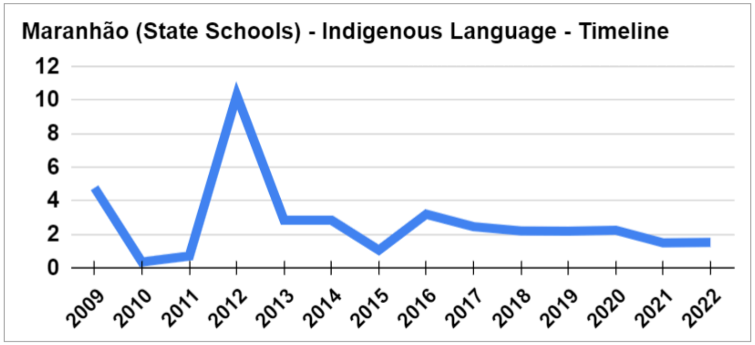

Indigenous Language - Municipal Schools

	2009	2010	2011	2012	2013	2014	2015	2016	2017	2018	2019	2020	2021	2022	Year
MA	0	0	0	8,57	0	0	0	0	0	7,94	7,94	8,7	9,59	9,86	%

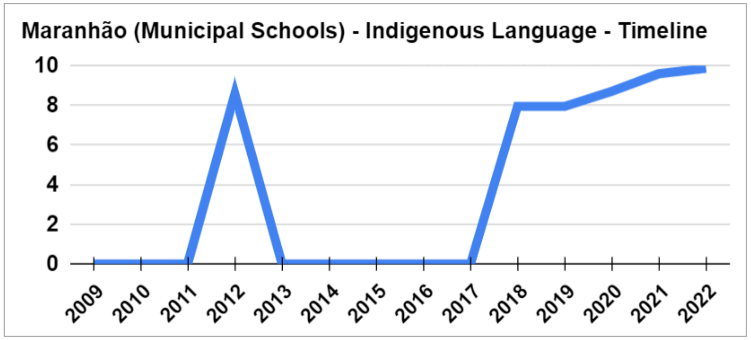

Number of State Schools

	2009	2010	2011	2012	2013	2014	2015	2016	2017	2018	2019	2020	2021	2022	Year
MA	251	278	283	263	281	267	283	281	284	272	273	267	266	263	n

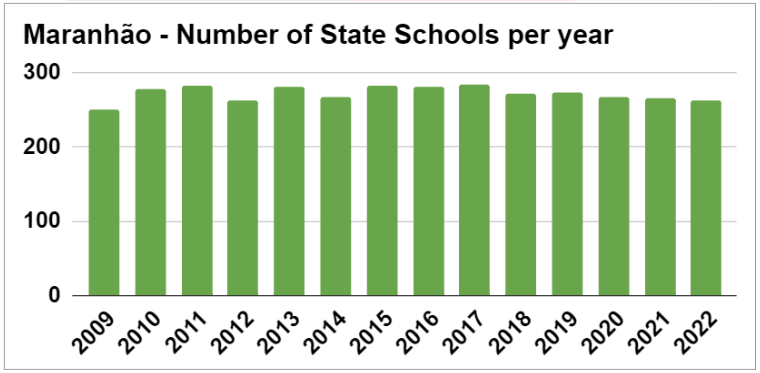

Number of Municipal Schools

	2009	2010	2011	2012	2013	2014	2015	2016	2017	2018	2019	2020	2021	2022	Year
MA	25	5	20	35	11	25	20	18	16	63	63	69	73	71	n

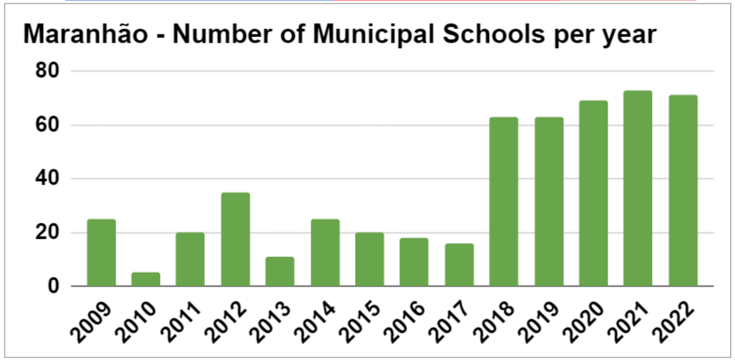

State of Bahia

Figure S24

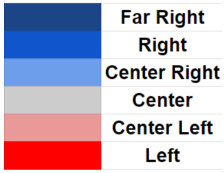

Indigenous Material - State Schools

	2009	2010	2011	2012	2013	2014	2015	2016	2017	2018	2019	2020	2021	2022	Year
BA	87,5	100	100	66,67	76,92	88,89	71,43	70,59	80	72,73	40	5	11,76	36,36	%

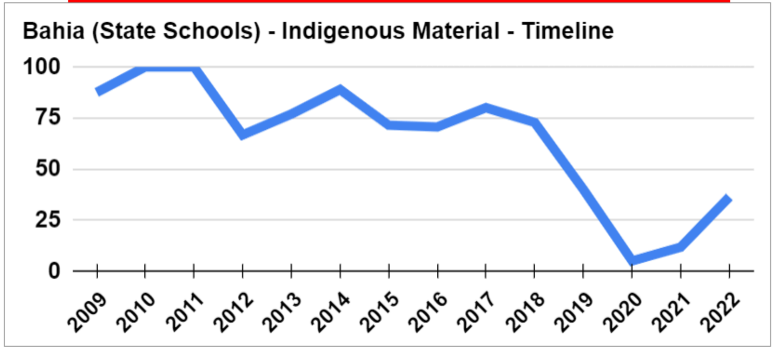

Indigenous Material - Municipal Schools

	2009	2010	2011	2012	2013	2014	2015	2016	2017	2018	2019	2020	2021	2022	Year
BA	66,67	68,89	64,1	85,71	66,67	72,41	96,43	75,76	62,16	67,57	30,56	41,67	33,33	36,11	%

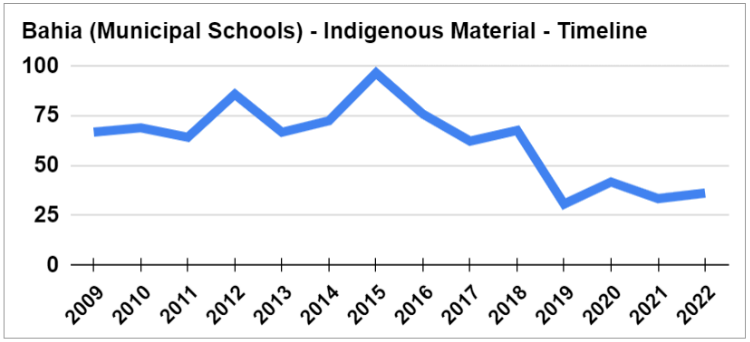

Indigenous Language - State Schools

	2009	2010	2011	2012	2013	2014	2015	2016	2017	2018	2019	2020	2021	2022	Year
BA	0	66,67	14,29	8,33	7,69	44,44	28,57	35,29	35	22,73	15	35	23,53	4,55	%

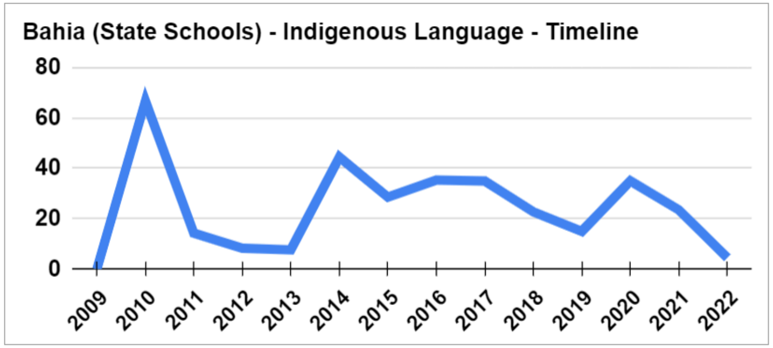

Indigenous Language - Municipal Schools

	2009	2010	2011	2012	2013	2014	2015	2016	2017	2018	2019	2020	2021	2022	Year
BA	0	0	2,56	3,57	3,33	0	3,57	3,03	0	0	2,78	2,78	0	0	%

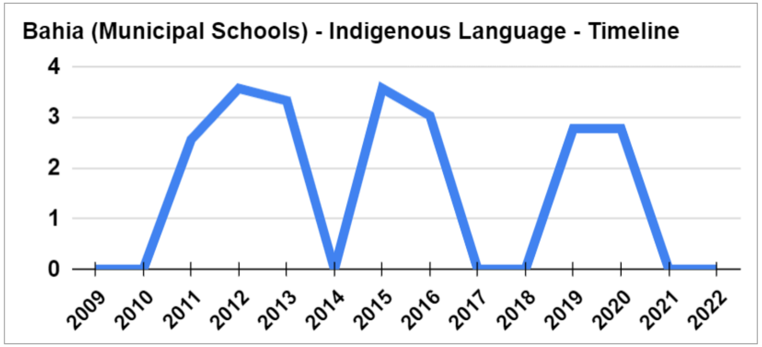

Number of State Schools

	2009	2010	2011	2012	2013	2014	2015	2016	2017	2018	2019	2020	2021	2022	Year
BA	8	3	7	12	13	9	14	17	20	22	20	20	17	22	n

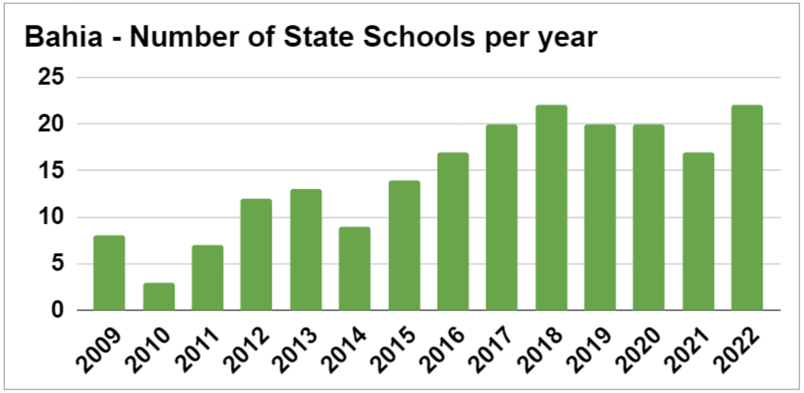

Number of Municipal Schools

	2009	2010	2011	2012	2013	2014	2015	2016	2017	2018	2019	2020	2021	2022	Year
BA	39	45	39	28	30	29	28	33	37	37	36	36	36	36	n

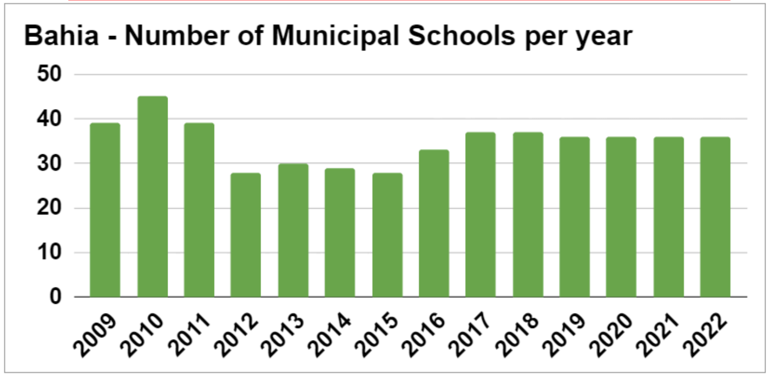

State of Sergipe

Figure S25

Legend
Far Right
Right
Center Right
Center
Center Left
Left

Indigenous Material - State Schools

	2009	2010	2011	2012	2013	2014	2015	2016	2017	2018	2019	2020	2021	2022	Year
SE	100	100	100	0	100	100	0	100	0	0	0	0	0	0	%

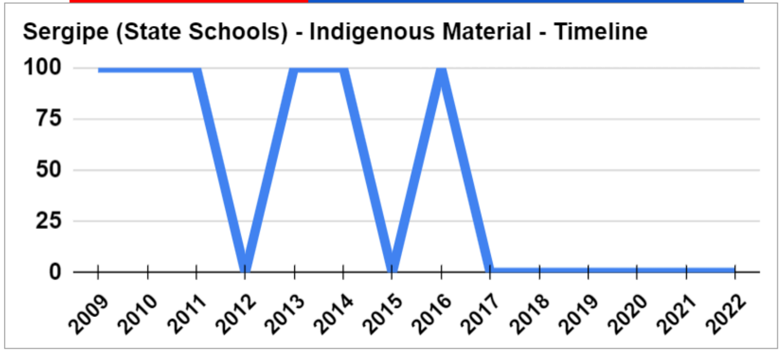

Indigenous Language - State Schools

	2009	2010	2011	2012	2013	2014	2015	2016	2017	2018	2019	2020	2021	2022	Year
SE	0	0	0	0	0	0	0	0	0	0	0	0	0	0	%

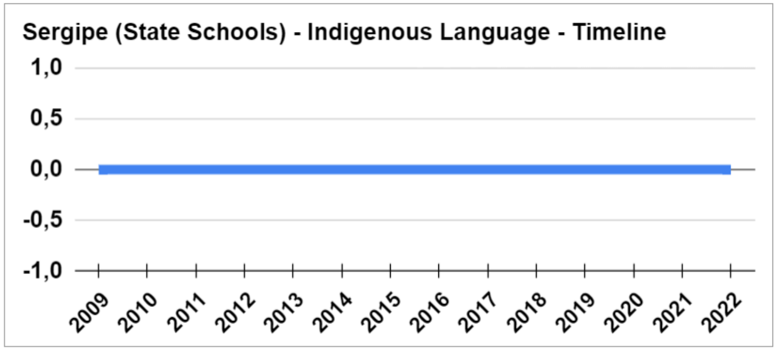

Number of State Schools

	2009	2010	2011	2012	2013	2014	2015	2016	2017	2018	2019	2020	2021	2022	Year
SE	1	1	1	1	1	1	1	1	1	1	1	1	1	1	%

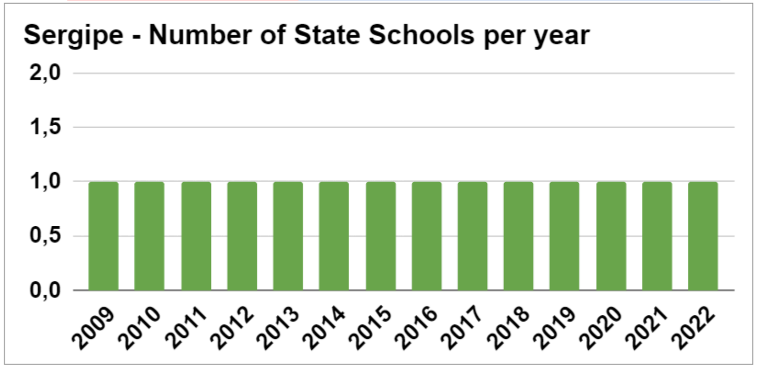

State of Alagoas

Figure S26

Legend
Far Right
Right
Center Right
Center
Center Left
Left

Indigenous Material - State Schools

	2009	2010	2011	2012	2013	2014	2015	2016	2017	2018	2019	2020	2021	2022	Year
AL	35,71	35,71	54,55	64,29	60	62,5	66,67	82,35	47,06	56,25	47,06	42,86	47,06	64,71	%

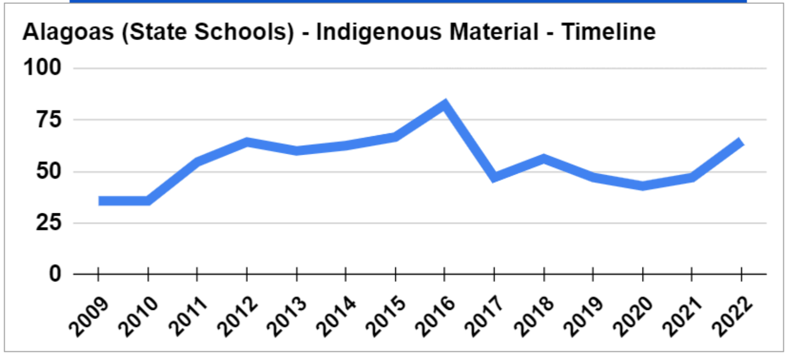

Indigenous Material - Municipal Schools

	2009	2010	2011	2012	2013	2014	2015	2016	2017	2018	2019	2020	2021	2022	Year
AL					0	0				100	0	0	0		%

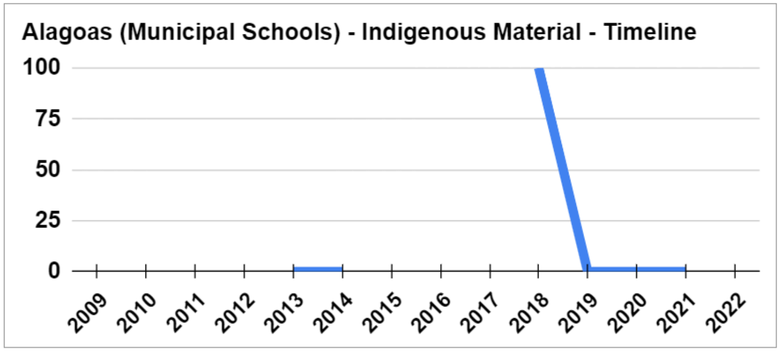

Indigenous Language - State Schools

	2009	2010	2011	2012	2013	2014	2015	2016	2017	2018	2019	2020	2021	2022	Year
AL	0	0	0	7,14	20	6,25	8,33	5,88	5,88	0	0	0	0	0	%

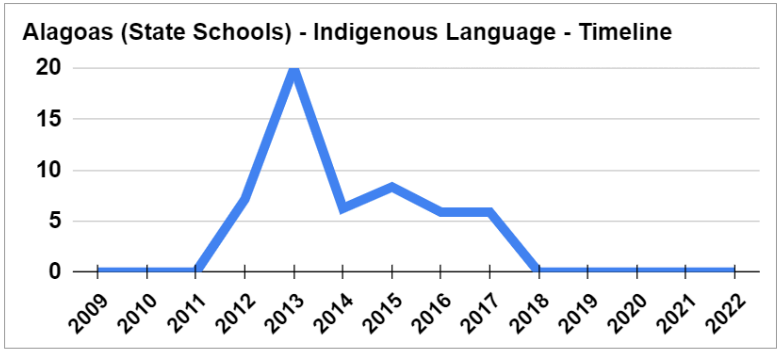

Indigenous Language - Municipal Schools

	2009	2010	2011	2012	2013	2014	2015	2016	2017	2018	2019	2020	2021	2022	Year
AL					0	0				0	0	0	0		%

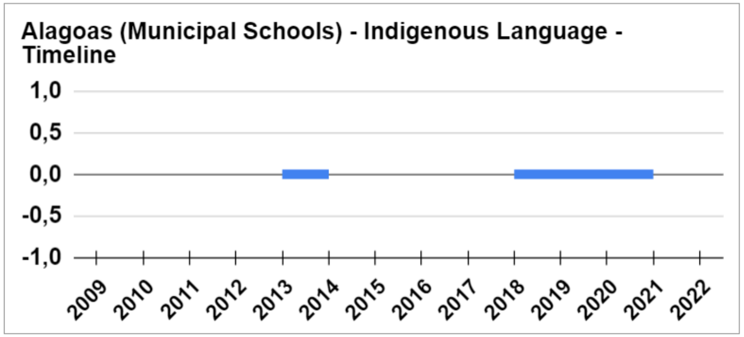

Number of State Schools

	2009	2010	2011	2012	2013	2014	2015	2016	2017	2018	2019	2020	2021	2022	Year
AL	14	14	11	14	15	16	12	17	17	16	17	14	17	17	n

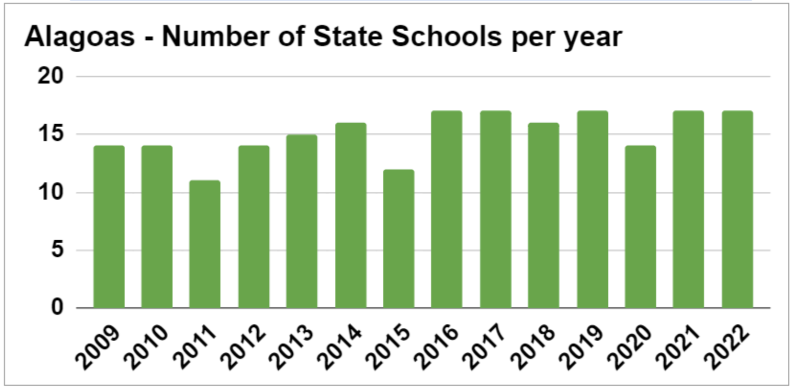

Number of Municipal Schools

	2009	2010	2011	2012	2013	2014	2015	2016	2017	2018	2019	2020	2021	2022	Year
AL					1	1				1	1	1	1		n

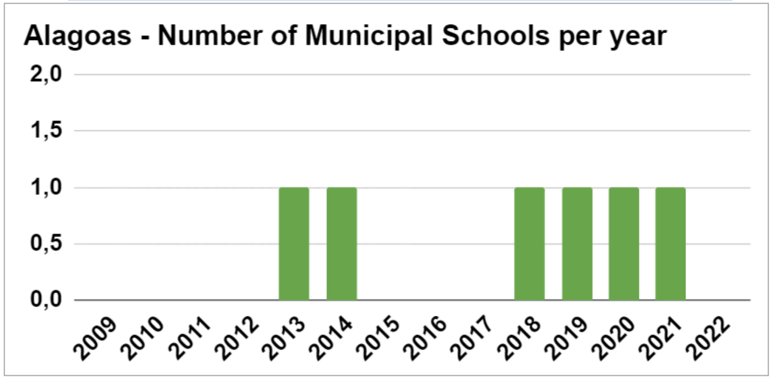

State of Pernambuco

Figure S27

Legend
Far Right
Right
Center Right
Center
Center Left
Left

Indigenous Material - State Schools

	2009	2010	2011	2012	2013	2014	2015	2016	2017	2018	2019	2020	2021	2022	Year
PE	77,59	65,04	70,34	71,93	84,8	54,62	61,83	69,01	70,83	64,83	33,56	45,95	43,92	42,58	%

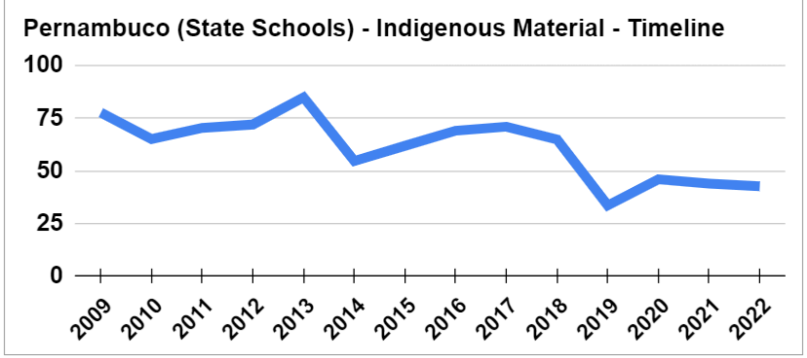

Indigenous Material - Municipal Schools

	2009	2010	2011	2012	2013	2014	2015	2016	2017	2018	2019	2020	2021	2022	Year
PE	50	0				0					0	0	0	0	%

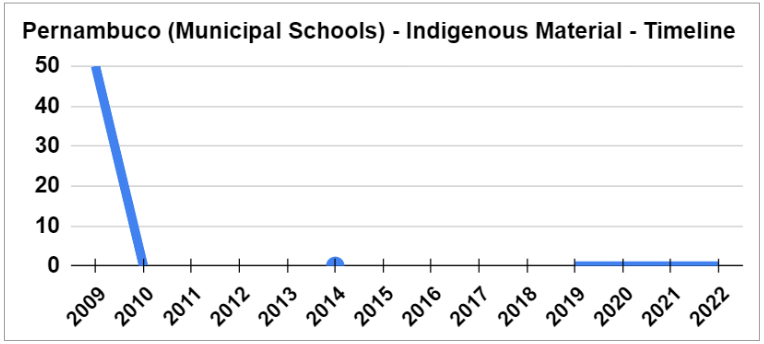

Indigenous Language - State Schools

	2009	2010	2011	2012	2013	2014	2015	2016	2017	2018	2019	2020	2021	2022	Year
PE	0	0	0	0	0	0	0,76	0,7	0,69	0,69	0,68	0	0	0,65	%

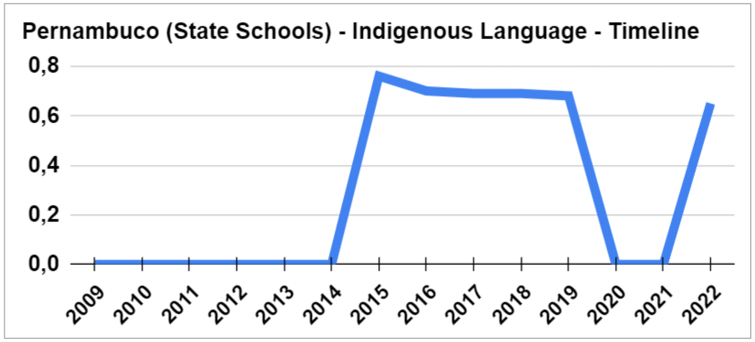

Indigenous Language - Municipal Schools

	2009	2010	2011	2012	2013	2014	2015	2016	2017	2018	2019	2020	2021	2022	Year
PE	0	0				0					0	0	0	0	%

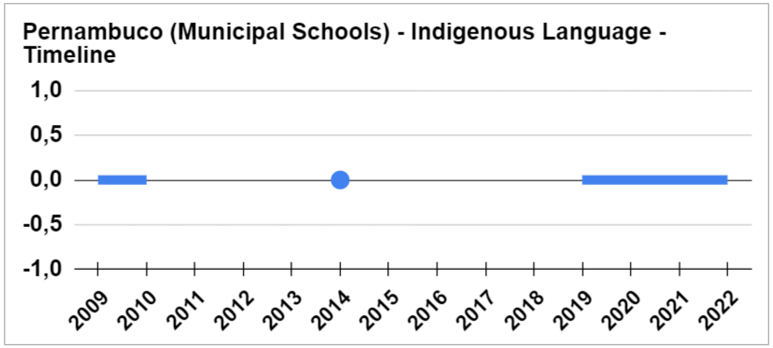

Number of State Schools

	2009	2010	2011	2012	2013	2014	2015	2016	2017	2018	2019	2020	2021	2022	Year
PE	116	123	118	114	125	130	131	142	144	145	146	148	148	155	n

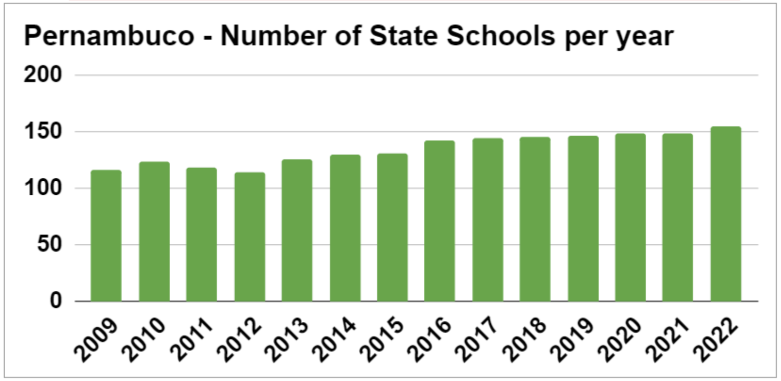

Number of Municipal Schools

	2009	2010	2011	2012	2013	2014	2015	2016	2017	2018	2019	2020	2021	2022	Year
PE	4	1				1					3	1	1	3	n

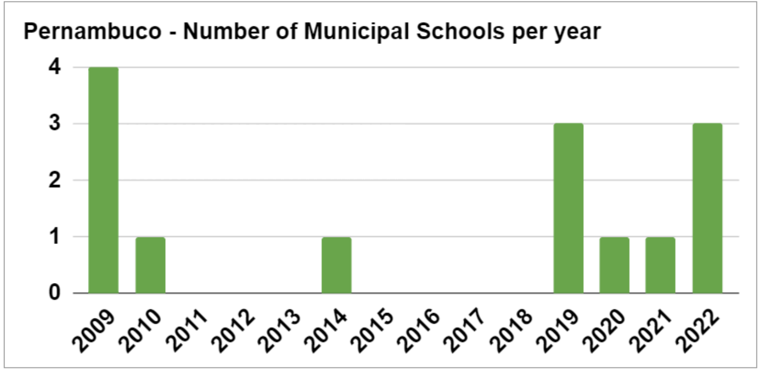

State of Paraíba

Figure S28

Far Right
Right
Center Right
Center
Center Left
Left

Indigenous Material - State Schools

	2009	2010	2011	2012	2013	2014	2015	2016	2017	2018	2019	2020	2021	2022	Year
PB	85,71	100	100	66,67	100	80	55,56	50	90	60	90,91	27,27	10	10	%

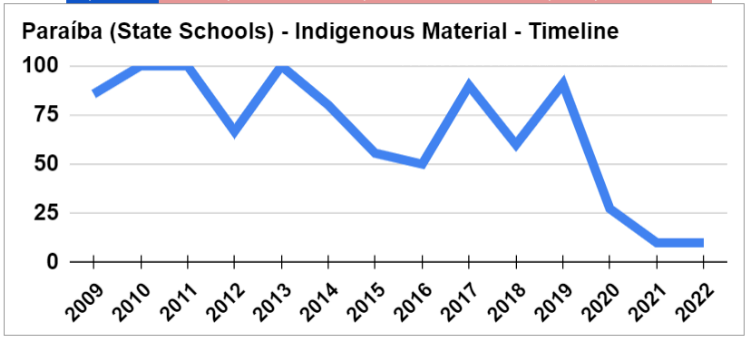

Indigenous Material - Municipal Schools

	2009	2010	2011	2012	2013	2014	2015	2016	2017	2018	2019	2020	2021	2022	Year
PB	71,43	85	94,12	57,14	81,82	60	68,75	60	10	5	13,04	25	25	45	%

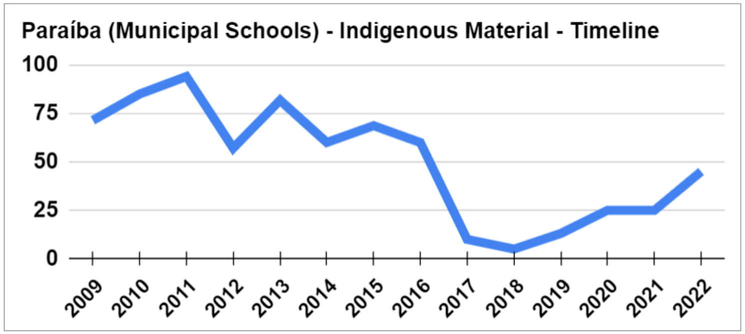

Indigenous Language - State Schools

	2009	2010	2011	2012	2013	2014	2015	2016	2017	2018	2019	2020	2021	2022	Year
PB	0	0	12,5	11,11	0	0	0	0	0	0	9,09	9,09	0	0	%

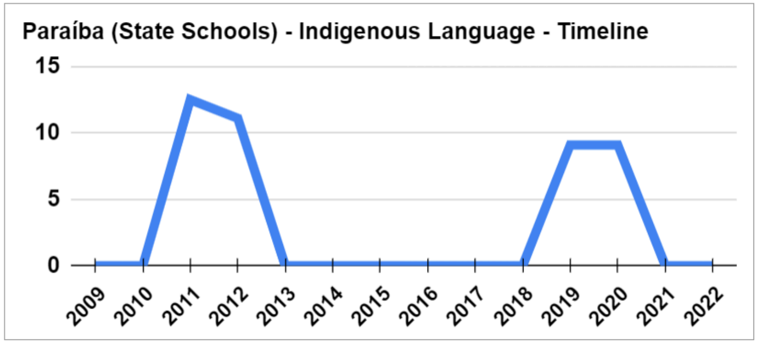

Indigenous Language - Municipal Schools

	2009	2010	2011	2012	2013	2014	2015	2016	2017	2018	2019	2020	2021	2022	Year
PB	0	0	0	0	36,36	30	62,5	10	5	0	0	0	0	20	%

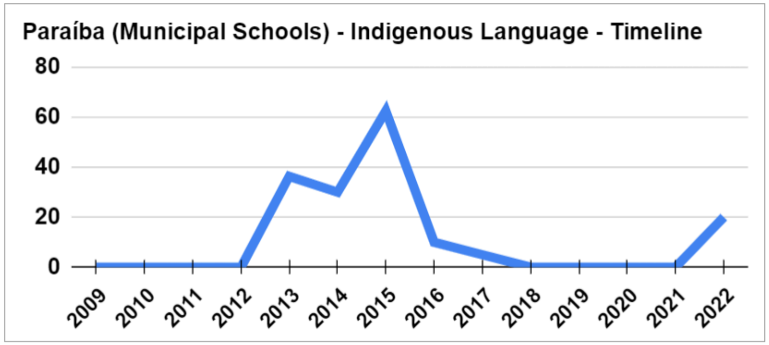

Number of State Schools

	2009	2010	2011	2012	2013	2014	2015	2016	2017	2018	2019	2020	2021	2022	Year
PB	7	9	8	9	9	10	9	10	10	10	11	11	10	10	n

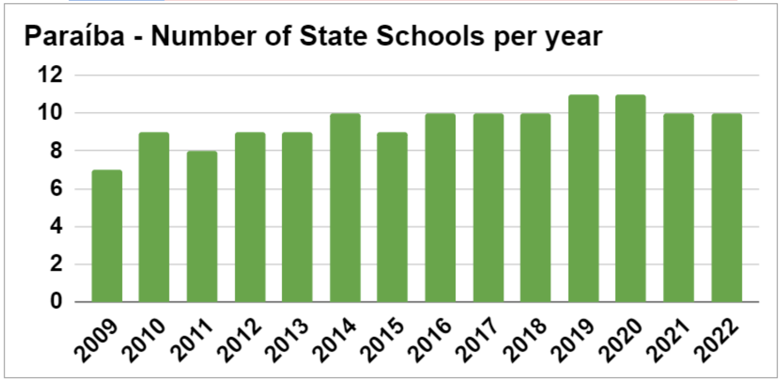

Number of Municipal Schools

	2009	2010	2011	2012	2013	2014	2015	2016	2017	2018	2019	2020	2021	2022	Year
PB	21	20	17	21	22	20	16	20	20	20	23	20	20	20	n

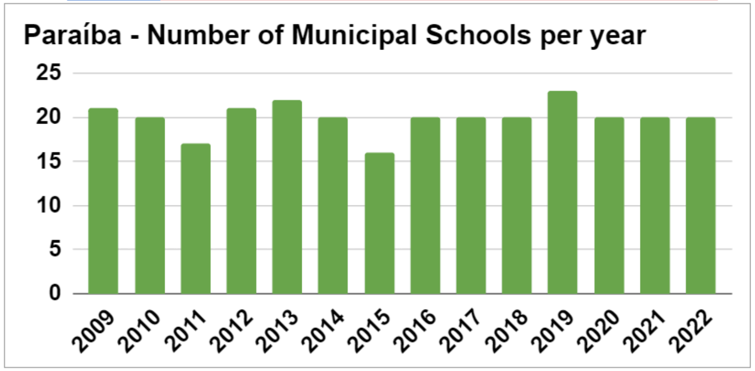

State of Rio Grande do Norte

Figure S29

Colour key
Far Right
Right
Center Right
Center
Center Left
Left

Indigenous Material - State Schools

	2009	2010	2011	2012	2013	2014	2015	2016	2017	2018	2019	2020	2021	2022	Year
RN												0	0	0	%

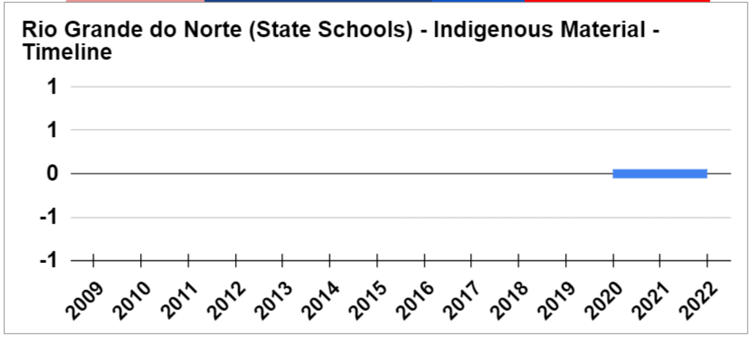

Indigenous Material - Municipal Schools

	2009	2010	2011	2012	2013	2014	2015	2016	2017	2018	2019	2020	2021	2022	Year
RN		0		0	100	100			0	25	22,22	22,22	0	0	%

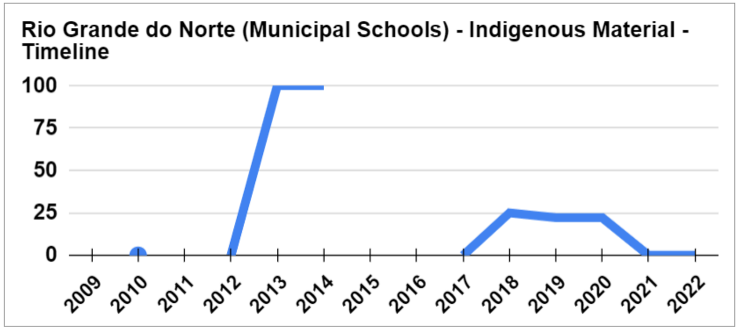

Indigenous Language - State Schools

	2009	2010	2011	2012	2013	2014	2015	2016	2017	2018	2019	2020	2021	2022	Year
RN												0	0	0	%

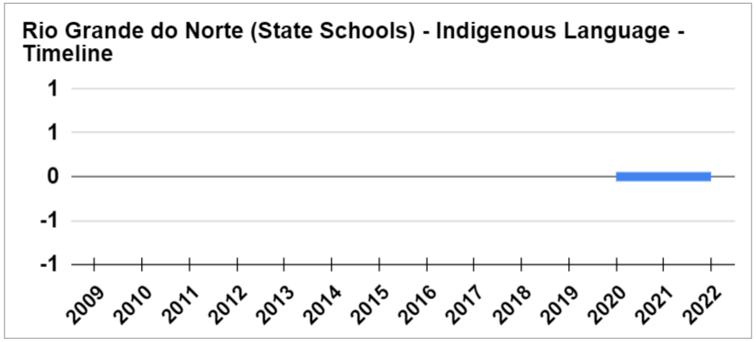

Indigenous Language - Municipal Schools

	2009	2010	2011	2012	2013	2014	2015	2016	2017	2018	2019	2020	2021	2022	Year
RN		100		100	0	0			0	0	0,00	0,00	0	0	%

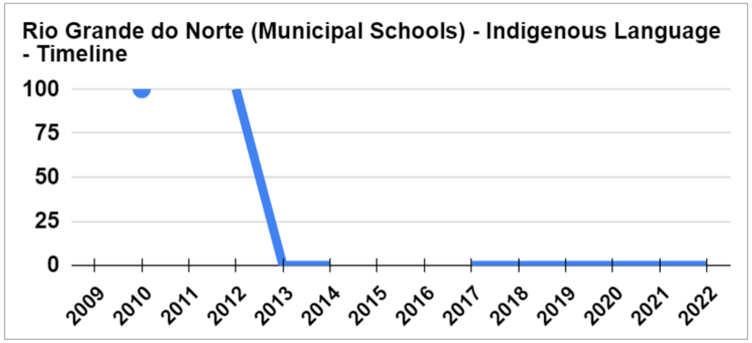

Number of State Schools

	2009	2010	2011	2012	2013	2014	2015	2016	2017	2018	2019	2020	2021	2022	Year
RN												1	1	1	n

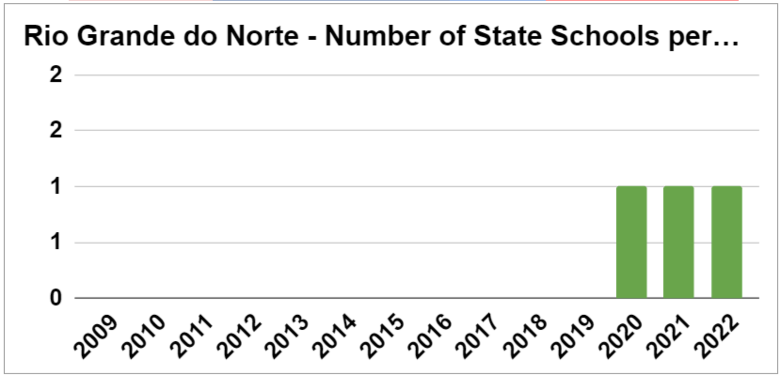

Number of Municipal Schools

	2009	2010	2011	2012	2013	2014	2015	2016	2017	2018	2019	2020	2021	2022	Year
RN		1		1	1	1			6	8	9,00	9,00	9	9	n

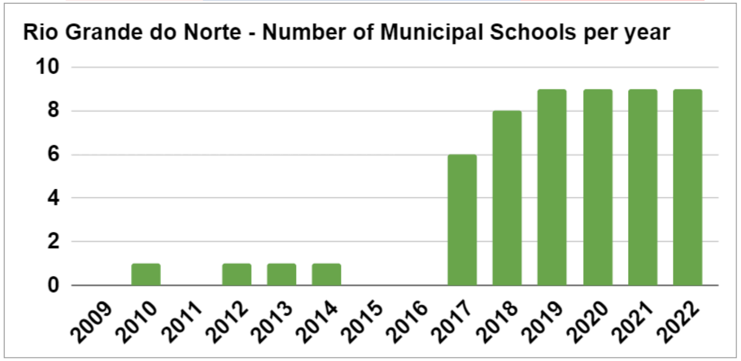

North East Region

Figure S30

Histogram - Indigenous Material (State)

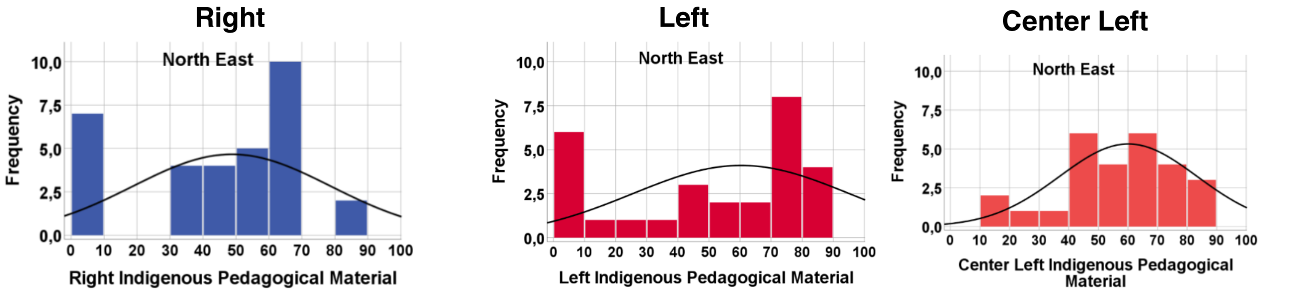

Histogram - Indigenous Material (Municipal)

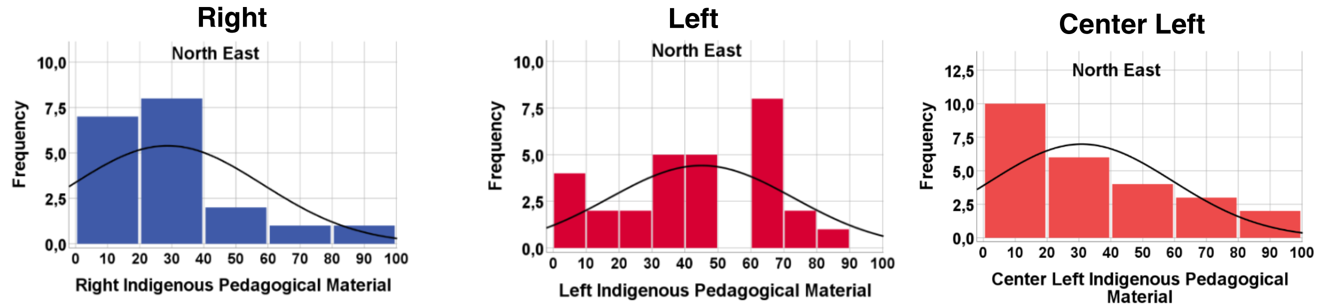

Histogram - Indigenous Language (State)

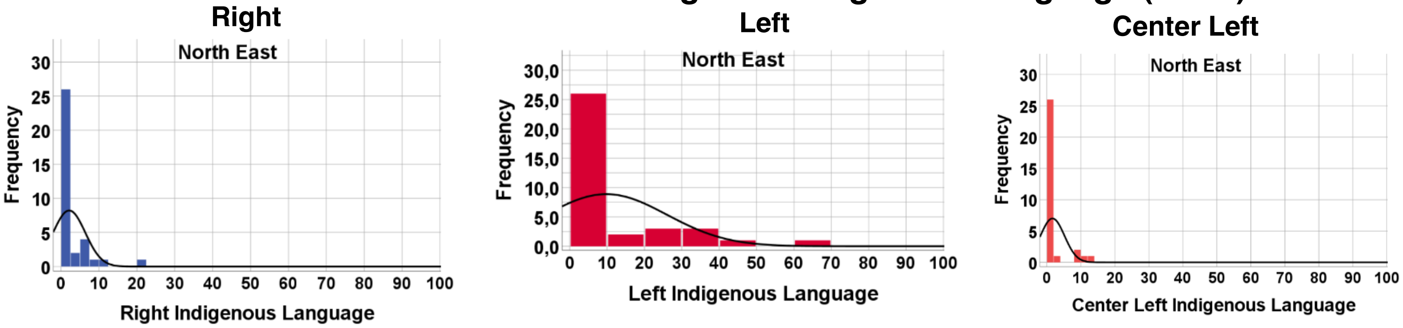

Histogram - Indigenous Language (Municipal)

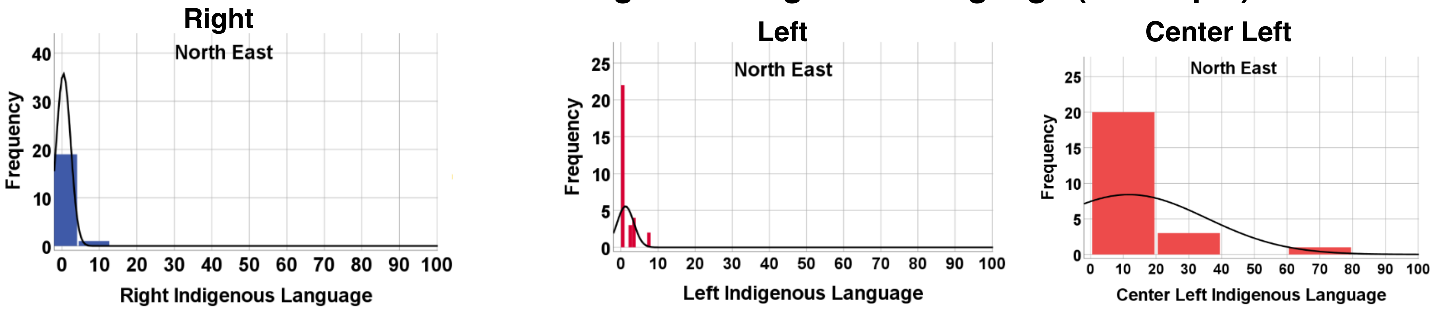

Brazil

Histogram - Indigenous Material (Municipal)

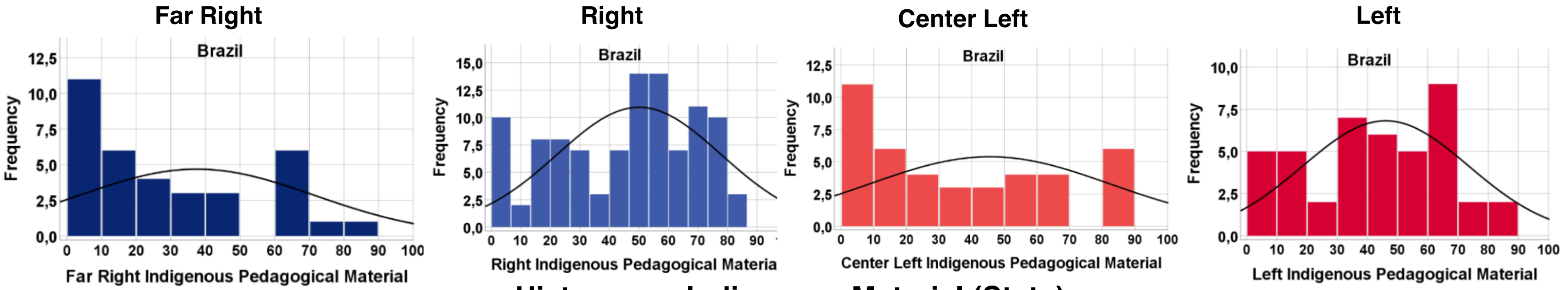

Histogram - Indigenous Material (State)

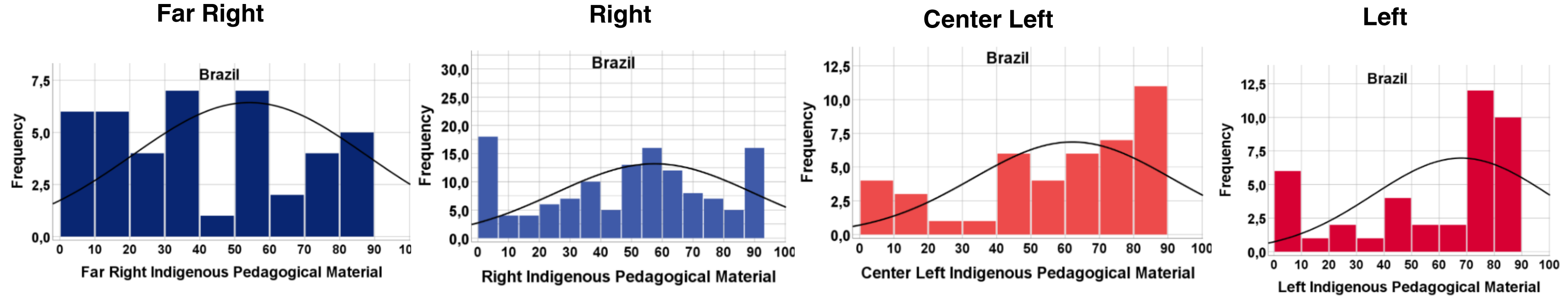

Histogram - Indigenous Language (Municipal)

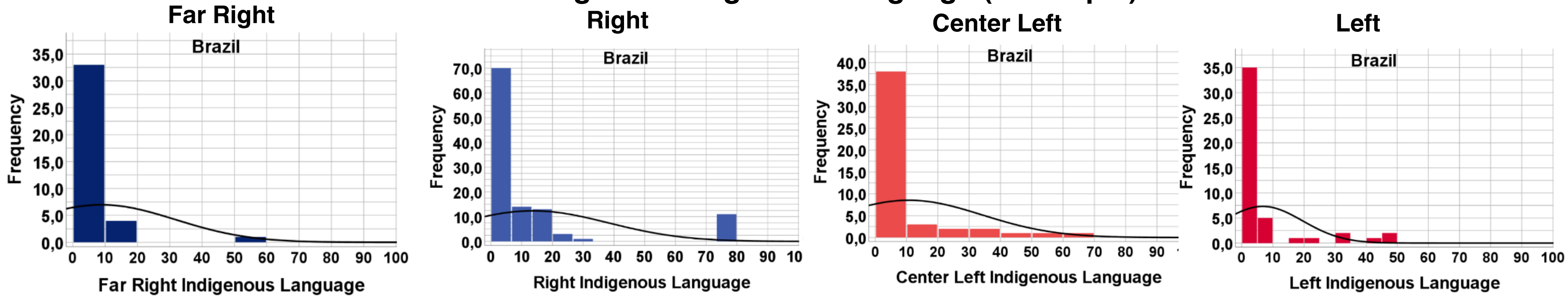

Histogram - Indigenous Language (State)

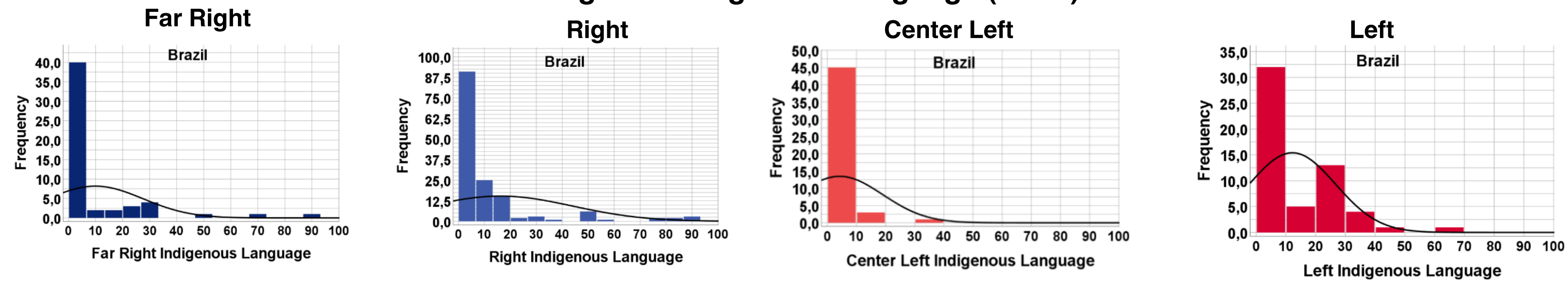

Influence of Far right Hard Core

Figure S32

Far Right Hard Core Governors x All others Governors

Indigenous Material - State School

Year	2019	2020	2021	2022	
Rest of the Governors (%)	38,8	48,6	47,8	49,3	%
Far Right Hard Core Governors (%)	19,2	22,5	24,5	24,8	%

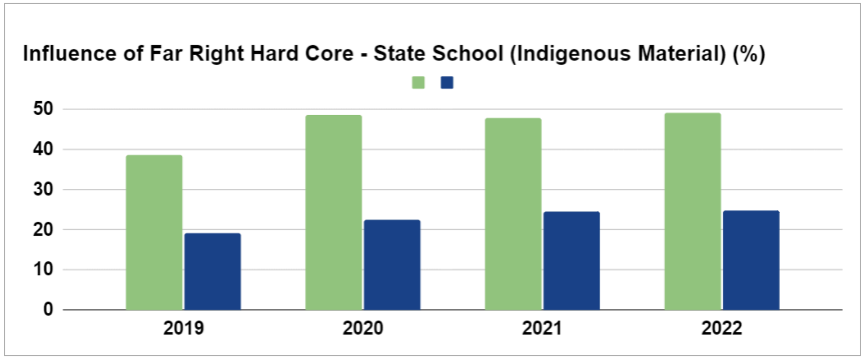

Indigenous Material - Municipal School

Year	2019	2020	2021	2022	
All Other Governors	30,7	37,7	38,1	44,4	%
Far Right Hard Core Governors (%)	29,4	37	39,8	41,6	%

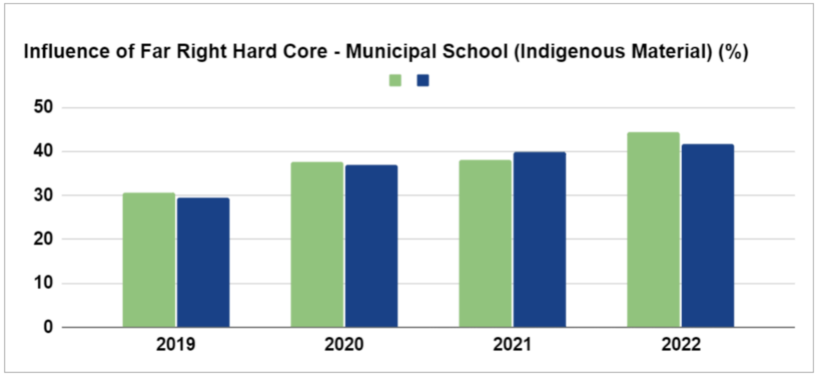

Indigenous Language - State School

Year	2019	2020	2021	2022	
All Other Governors	7,4	8,3	7,7	6,7	%
Far Right Hard Core Governors (%)	2,5	2,3	1,8	1,6	%

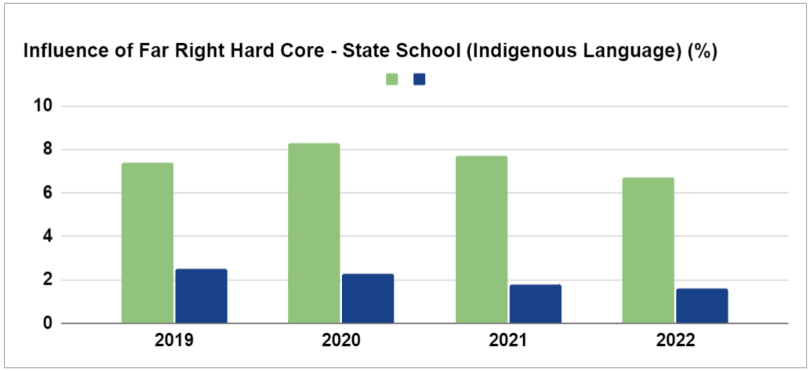

Indigenous Language - Municipal School

Year	2019	2020	2021	2022	
All Other Governors	4,6	5,5	5,1	6	%
Far Right Hard Core Governors (%)	1,2	1,3	2,3	1,5	%

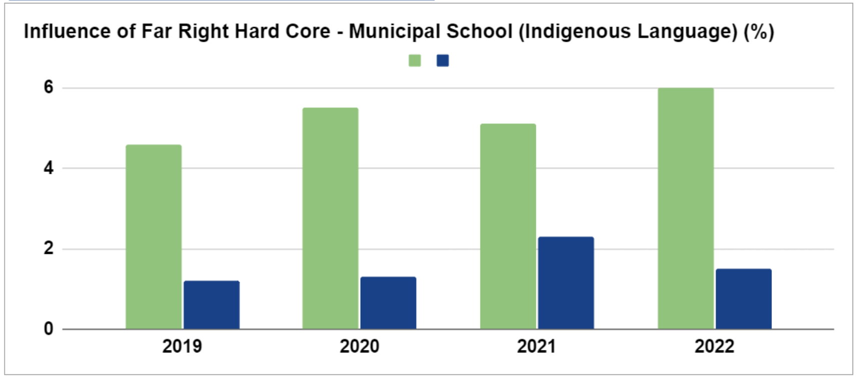

Political Spectrum Behavior Overtime

Figure S33

Indigenous Language (State)

Center Left

Center Left														
Year	2009	2010	2011	2012	2013	2014	2015	2016	2017	2018	2019	2020	2021	2022
%	0	0	1,10	1,10	0	8,7	2,1	0,5	1,2	1,6	0,9	1,5	1	1

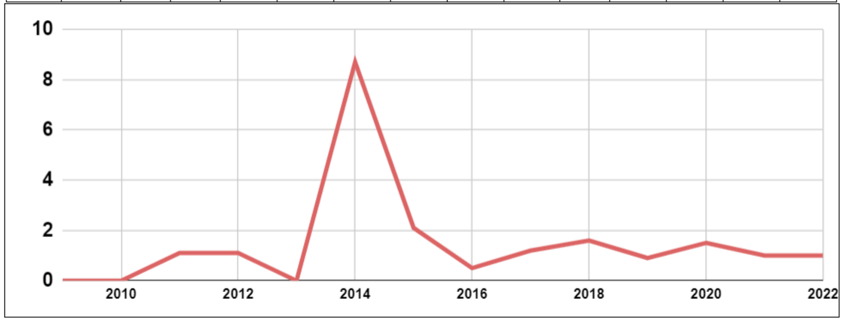

Right

Right														
Year	2009	2010	2011	2012	2013	2014	2015	2016	2017	2018	2019	2020	2021	2022
%	6	6	6,3	13,6	14	15,9	18,3	13,3	11	6,7	16,9	26,2	25,1	15,3

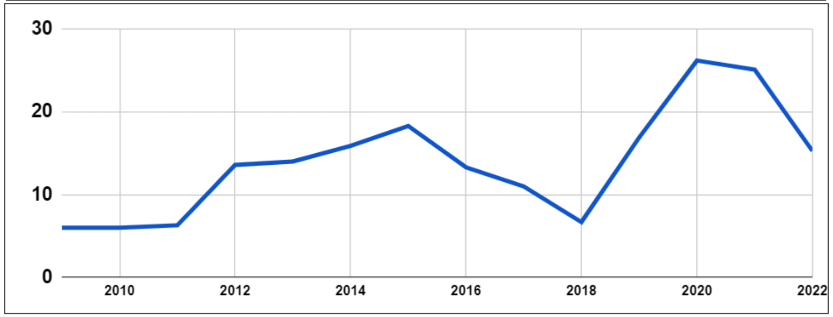

Left

Left														
Year	2009	2010	2011	2012	2013	2014	2015	2016	2017	2018	2019	2020	2021	2022
%	25,8	22,2	10,2	21,7	19,7	17,4	6,1	4,9	4,7	3,1	2,7	11,7	7	1,6

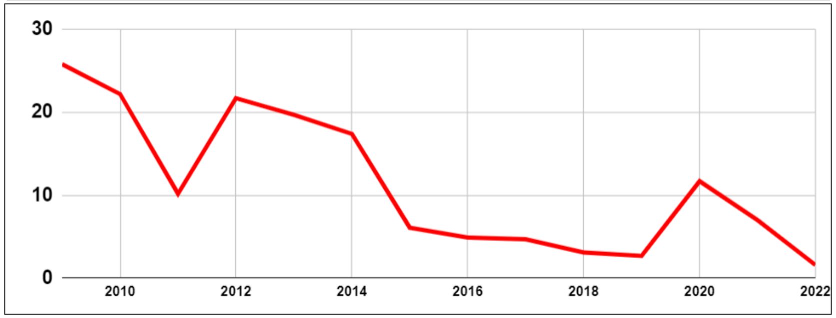

Far Right

Far Right														
Year	2009	2010	2011	2012	2013	2014	2015	2016	2017	2018	2019	2020	2021	2022
%	50	0	11,8	3,1	3,2	8,7	1,9	2,1	1,4	11,7	2,9	2,9	2,7	2,7

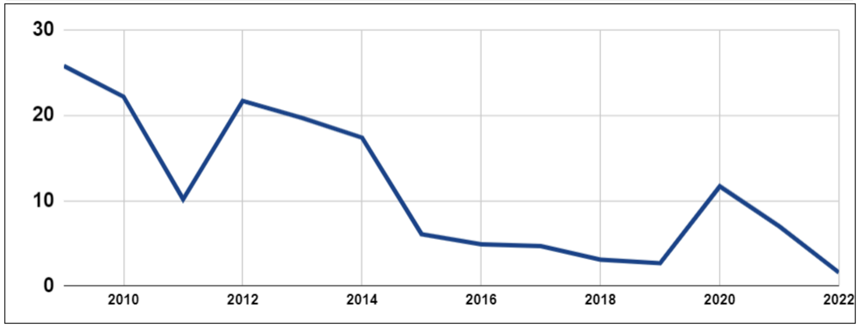

Center Right			
Year	2010	2014	2018
%	0,0%	2,4%	1,9%

Center		
Year	2009	2010
%	3,1	1,3

Political Spectrum Behavior Overtime

Figure S34

Indigenous Language (Municipal)

Center Left

	Center Left													
Year	2009	2010	2011	2012	2013	2014	2015	2016	2017	2018	2019	2020	2021	2022
%	0	16,7	3,70	6,10	20,5	14,3	60,9	12	0,1	0,5	0	5,8	6,5	10,2

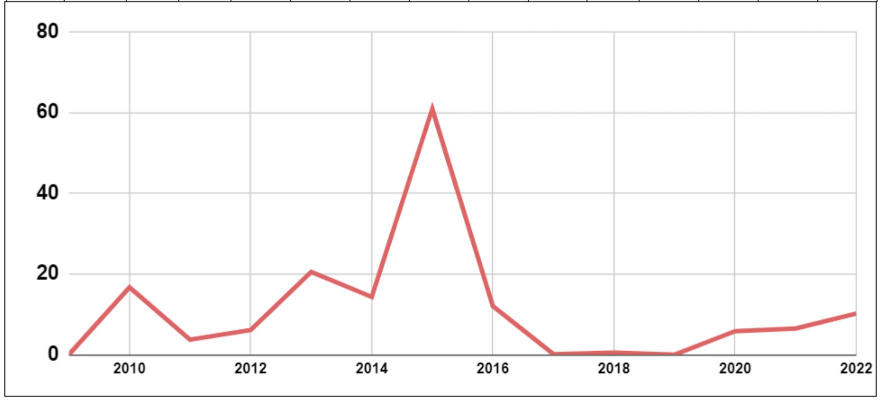

Right

	Right													
Year	2009	2010	2011	2012	2013	2014	2015	2016	2017	2018	2019	2020	2021	2022
%	6,2	9,5	4,9	10,4	21,5	3,8	5,9	2	5,8	5,6	5,1	6,2	5,2	5,4

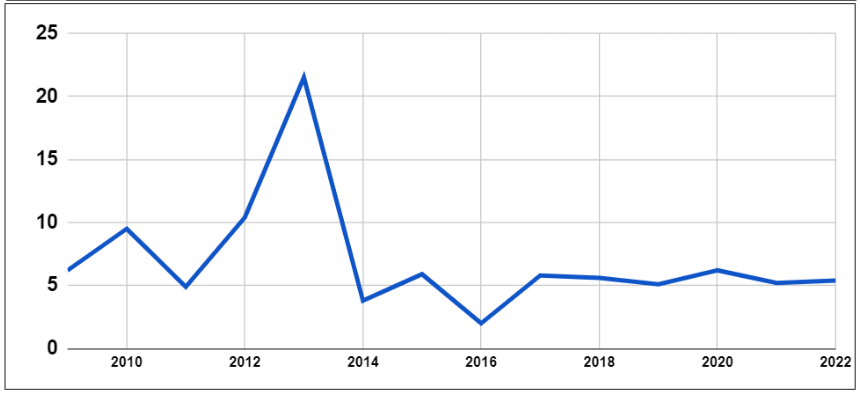

Left

	Left													
Year	2009	2010	2011	2012	2013	2014	2015	2016	2017	2018	2019	2020	2021	2022
%	4,7	15,2	3,8	4,9	5,6	1,9	28	26,7	18,2	6,5	5,3	2	0	0

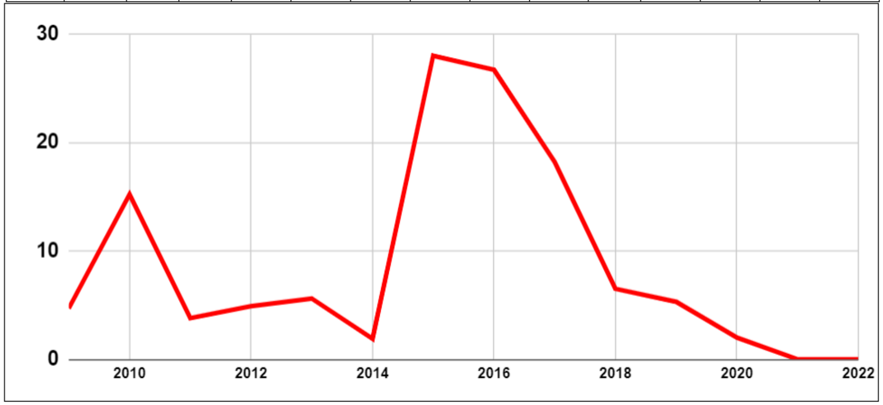

Far Right

	Far Right													
Year	2009	2010	2011	2012	2013	2014	2015	2016	2017	2018	2019	2020	2021	2022
%		0	16,7	57,1	0	0	3,4	0	0	0,8	1,2	1,3	2,4	1,5

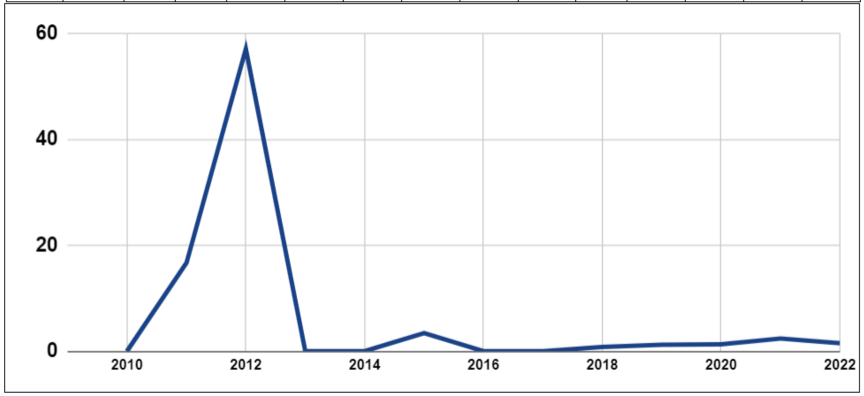

Center Right		
Year	2010	2014
%	3,6	50

Center		
Year	2009	2010
%	7,3	0

Political Spectrum Behavior Overtime

Figure S35

Indigenous Material (Municipal)

Center Left

	Center Left													
Year	2009	2010	2011	2012	2013	2014	2015	2016	2017	2018	2019	2020	2021	2022
%	55,6	16,7	74,10	69,70	84,6	22,1	78,3	48	39,9	48,3	25	33,7	31,5	36,1

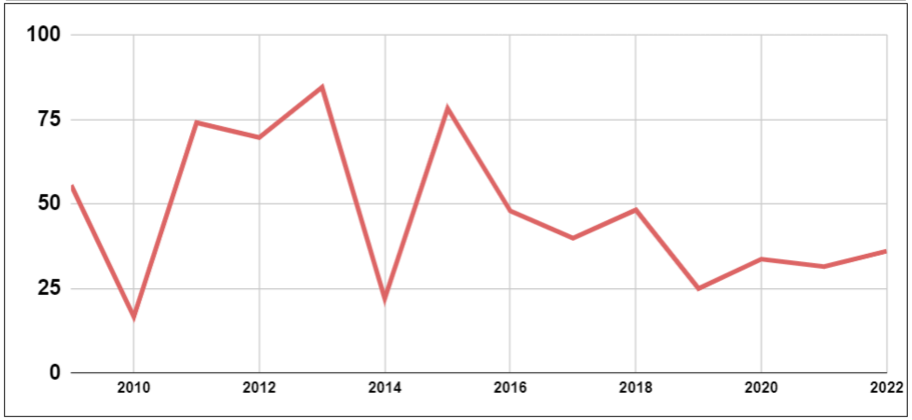

Right

	Right													
Year	2009	2010	2011	2012	2013	2014	2015	2016	2017	2018	2019	2020	2021	2022
%	26,1	63,6	40,5	53,7	57,9	44,5	52,9	46,2	56,2	57,5	36,4	39,3	42,8	49,5

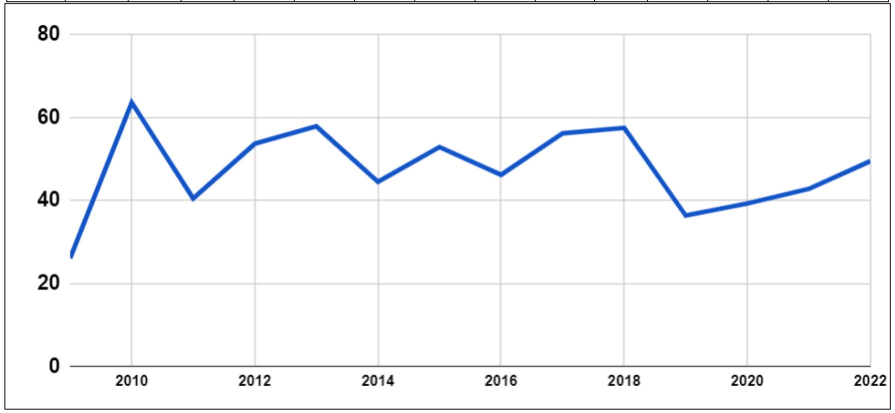

Left

	Left													
Year	2009	2010	2011	2012	2013	2014	2015	2016	2017	2018	2019	2020	2021	2022
%	59,7	58,7	55,7	87,3	57,9	59,8	33,6	33,3	54,7	20,3	19,5	38	28	30

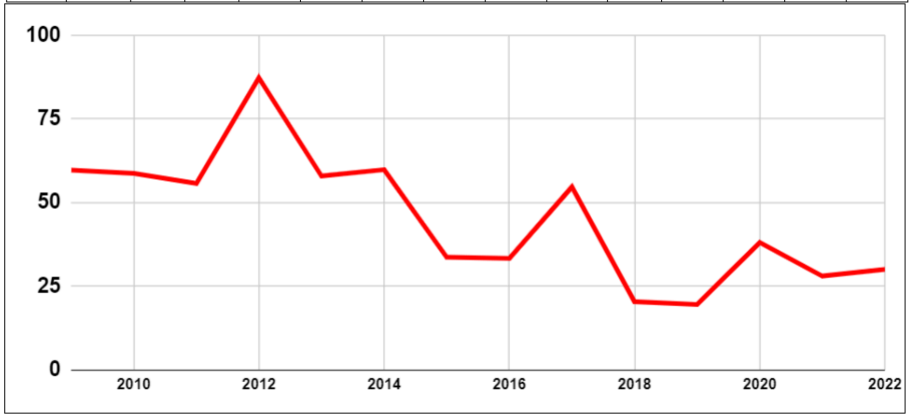

Far Right

	Far Right													
Year	2009	2010	2011	2012	2013	2014	2015	2016	2017	2018	2019	2020	2021	2022
%		100	66,7	57,1	100	100	21,8	27,6	4,4	18,9	29,4	36,9	39,6	41,6

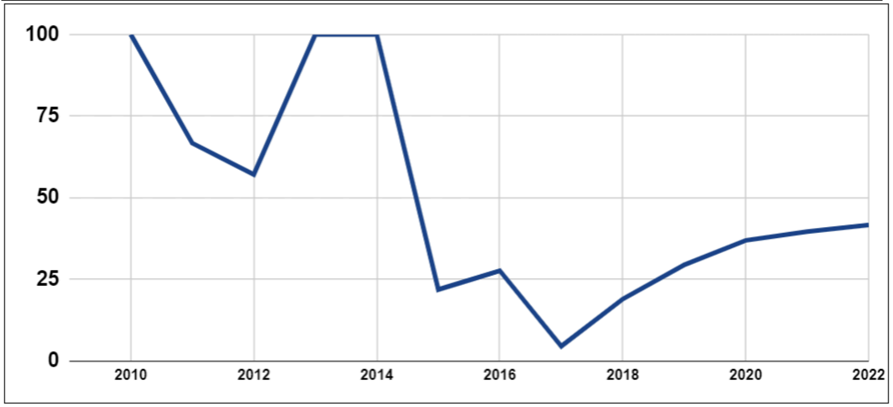

Center Right		
Year	2010	2014
%	49,3	100

Center		
Year	2009	2010
%	75,2	40

Political Spectrum Behavior Overtime

Figure S36

Indigenous Material (State)

Center Left

	Center Left													
Year	2009	2010	2011	2012	2013	2014	2015	2016	2017	2018	2019	2020	2021	2022
%	83,4	63,3	77,30	76,80	86,2	29,7	67,9	73,3	62,5	64,3	46,9	46,9	45,8	45,3

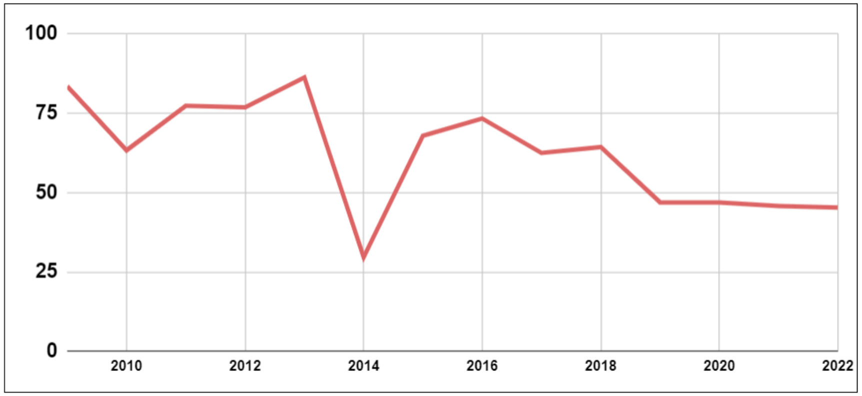

Right

	Right													
Year	2009	2010	2011	2012	2013	2014	2015	2016	2017	2018	2019	2020	2021	2022
%	42	51,6	48,8	49,9	51	56,8	70,4	68,5	83,3	67	39,1	45,1	40,7	48,7

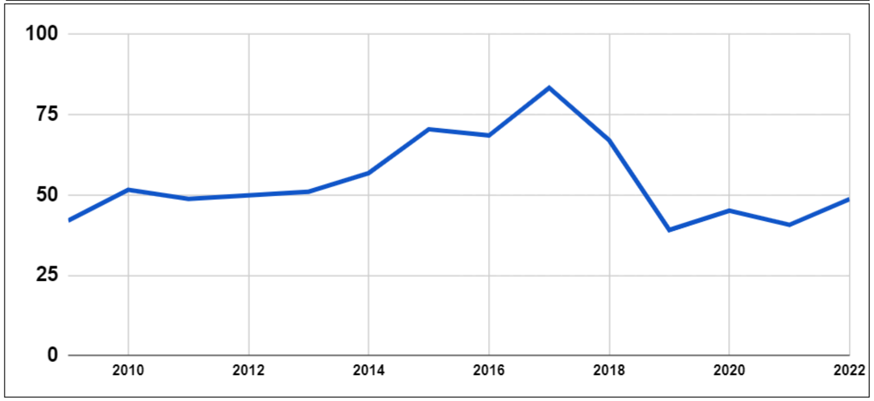

Left

	Left													
Year	2009	2010	2011	2012	2013	2014	2015	2016	2017	2018	2019	2020	2021	2022
%	81,3	77,8	86,1	86,9	77,9	86,7	73,3	86	80,2	86,5	29,2	50	56,1	61,3

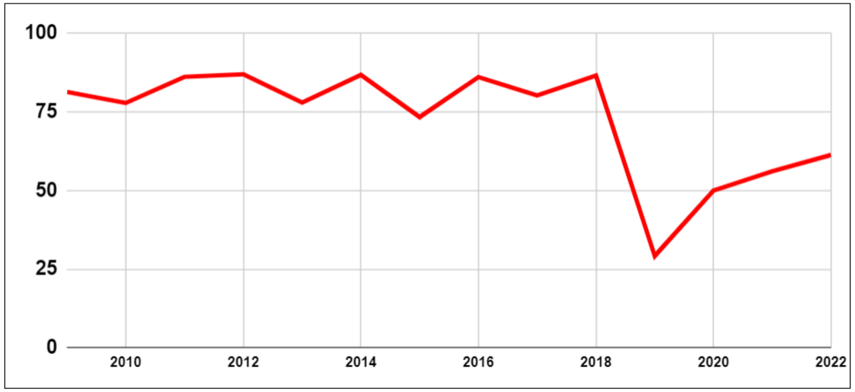

Far Right

	Far Right													
Year	2009	2010	2011	2012	2013	2014	2015	2016	2017	2018	2019	2020	2021	2022
%	50	98,3	82,4	87,5	96,8	89,1	16,3	15,7	22,4	26,5	22,7	29,3	31,6	28,7

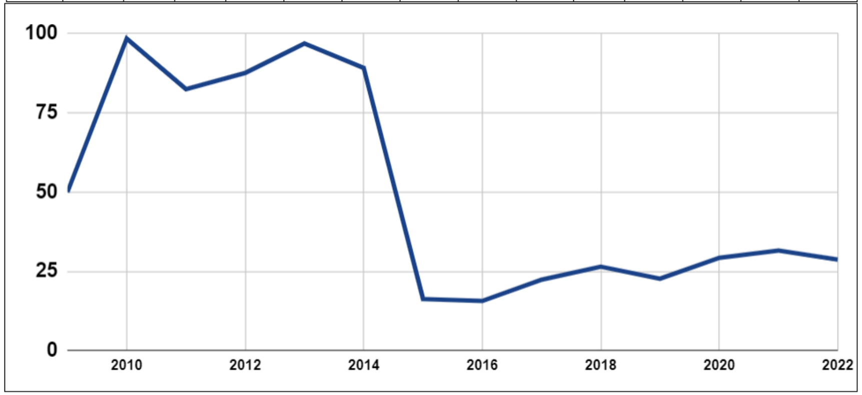

	Center Right		
Year	2010	2014	2018
%	27,3	90,2	55,8

	Center	
Year	2009	2010
%	87,4	86,7

School Per Year

Figure S37

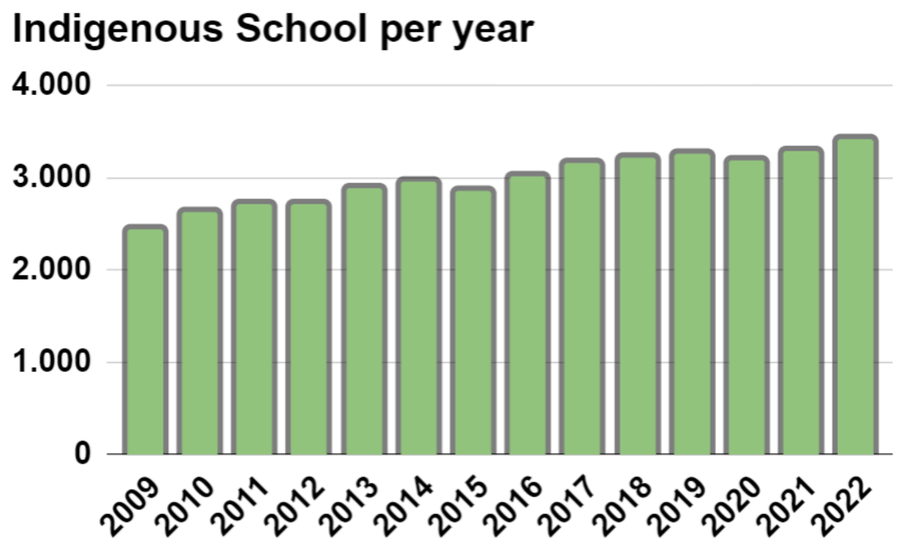

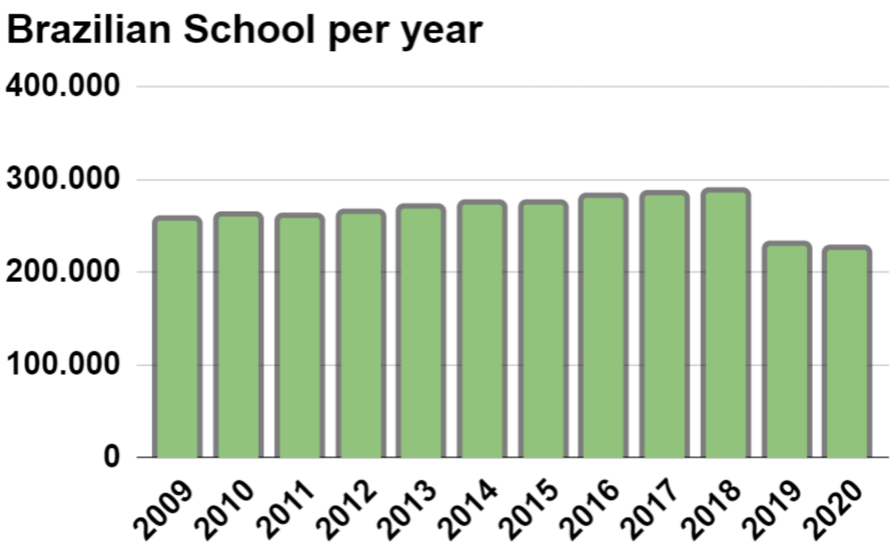

School Census	Indigenous School	Schools in Brazil
**2009**	2.441	255.445
**2010**	2.623	259.831
**2011**	2.714	257.880
**2012**	2.716	262.601
**2013**	2.886	268.166
**2014**	2.960	272.440
**2015**	2.864	272.996
**2016**	3.011	279.358
**2017**	3.152	282.362
**2018**	3.219	286.014
**2019**	3.256	228.521
**2020**	3.188	224.229
**2021**	3.294	221.140
**2022**	3.411	224.649

Indigenous Behavior State / Year

Figure S38

Indigenous Material (State)

State	2019	2020	2021	2022	%
RO	25,5	33,7	32	34,7	%
AC	22,2	27	30,4	29,7	%
AM	19,2	15,1	17,9	17	%
RR	4,3	4,8	4,5	4	%
PA	22,2	50	0	0	%
AP	74,1	79,6	81,1	77,8	%
TO	47,3	55,3	59,6	54,1	%
MA	22	41,6	41,4	41,8	%
CE	74,4	74,4	76,9	76,9	%
RN	22,2	0	0	0	%
PB	90,9	27,3	10	10	%
PE	33,6	45,9	43,9	42,6	%
AL	47,1	42,9	47,1	64,7	%
SE	0	0	0	0	%
BA	40	5	11,8	36,4	%
MG	47,4	52,6	52,6	55	%
ES	0	0	0	0	%
RJ	100	100	100	100	%
SP	25	51,3	40,7	52,6	%
PR	81,6	87,2	86,1	89,5	%
SC	76,7	70	68,8	87,5	%
RS	21,2	22,1	32,9	32,9	%
MS	33,1	40	29,4	35,3	%
MT	57,1	65,7	71	75,7	%
GO	33,3	33,3	33,3	100	%

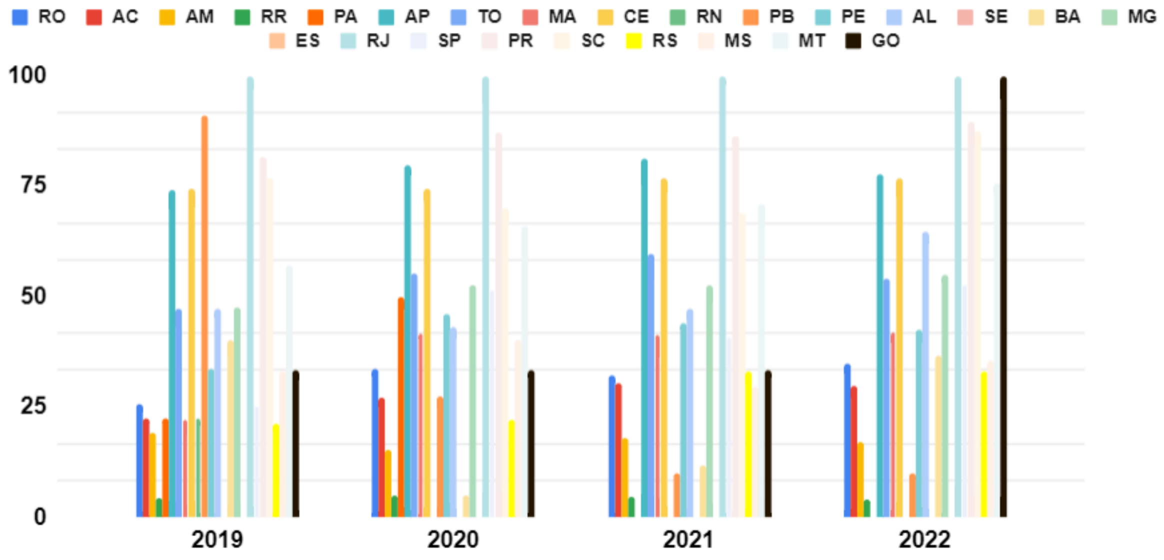

Indigenous Behavior State / Year

Figure S39

Indigenous Material (Municipal)

State	2019	2020	2021	2022	%
AC	0	13,6	11,1	11,1	%
AM	30,3	38,4	44,5	46,3	%
RR	2,1	6,7	4,6	5,4	%
PA	29,8	33,3	36,3	44,1	%
AP	12,5	12,5	0	0	%
TO	33,3	33,3	0	66,7	%
MA	9,5	33,3	32,9	35,2	%
CE	60	40	40	40	%
RN	0	22,2	0	0	%
PB	13	25	25	45	%
PE	0	0	0	0	%
AL	0	0	0	0	%
BA	30,6	41,7	33,3	36,1	%
MG	100	100	83,3	83,3	%
RJ	0	0	0	0	%
SP	50	25	50	50	%
SC	28,6	28,6	14,3	28,6	%
RS	16,7	42,9	57,1	57,1	%
MS	72,1	72,7	76,1	78,7	%
MT	63,3	64,4	63	69,7	%
GO	0	0	0	0	%

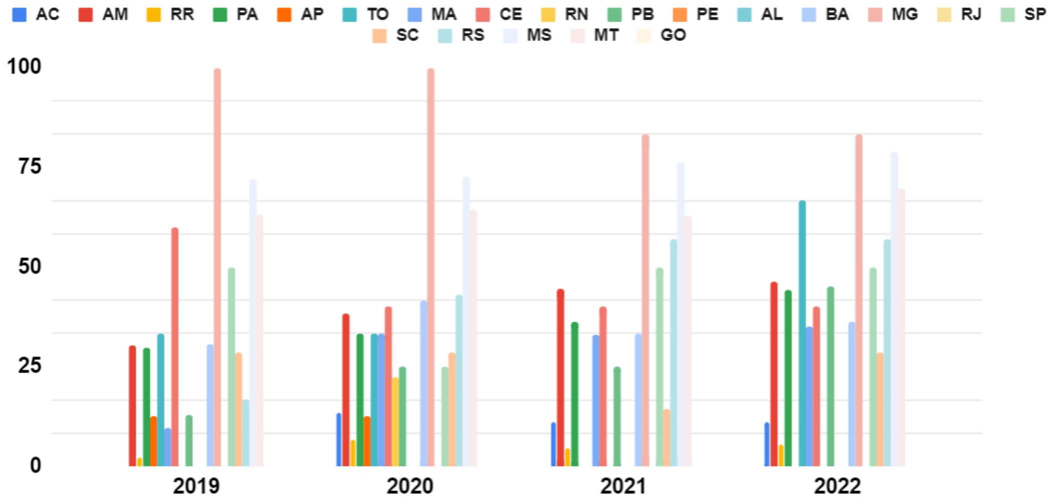

Statistical Test

Figure S40

Hypothesis Test	Number of Valid Cases	gl	Pearson Chi-Square	Likelihood Ratio	Nominal by Nominal		Asymptotic Significance (Bilateral)	p-value	Data Clipping
					Fi	V for Cramer			
Indigenous Material - Influence of the Mayor's ideology	2.468	855	896,348	606,951	0,603	0,270	0,159	0,159 (p > 0,05)	County
Indigenous Language Only - Influence of the Mayor's ideology	2.468	390	377,622	281,140	0,391	0,175	0,664	0,664 (p > 0,05)	County
Indigenous Material - Influence of the Governor's ideology (State Schools)	326	1.025	1.155,089	622,86	1,882	0,842	0,003	**0,003 (p < 0,05)**	State
Indigenous Material - Influence of the Governor's ideology (Municipal Schools)	256	635	696,784	421,65	1,650	0,738	0,045	**0,045 (p < 0,05)**	State
Indigenous Language Only - Influence of the Governor's ideology (State Schools)	326	745	908,065	495,394	1,669	0,746	0,000	**0,000 (p < 0,05)**	State
Indigenous Language Only - Influence of the Governor's ideology (Municipal Schools)	256	450	572,147	298,003	1,495	0,669	0,000	**0,000 (p < 0,05)**	State

Political Spectrum

Political Spectrum						
**Far Right**	**Right**	**Center Right**	**Center**	**Center Left**	**Left**	**Far Left**
Democrats - DEM	Brazilian Democratic Movement Party - MDB	Humanist Solidarity Party - PHS	Socialist People's Party - PPS	Democratic Labor Party - PDT	Communist Party of Brazil - PC do B	Party of Socialism and Freedom - PSOL
Christian Social Democratic Party - PSDC	Free Fatherland Party - PPL	Brazilian Women's Party - PMB	Green Party - PV	Brazilian Socialist Party - PSB	Workers' Party - PT	
National Ecological Party - PEN	Party of the Republic - PR	National Mobilization Party - Mobiliza	REDE (Network)			
Liberal Party - PL	Brazilian Republican Party - PRB	Brazilian Labor Party - Avante				
Progressives - PP	Republican Party of Social Order - PROS	Brazilian Worker's Party - PTB				
Progressive Republican Party - PRP	Brazilian Renewal Labor Party - PRTB	Solidarity - SD				
Christian Social Party - PSC	Social Democratic Party - PSD					
Social Liberal Party - PSL	Brazilian Social Democracy Party - PSDB					
NOVO	Christian Labor Party - Agir					
Union Brazil	National Labor Party - PTN					
	Republicans					
	Podemos (We can)					

Figure S41

Estimated Marginal Means of Governors' Ideology on Indigenous School Language Practices (State Level)

Indigenous Material

Model Coefficients					
Ideology	Estimate	Std. Error	df	t value	Pr(>|t|)
Left	85.5853	10.6823	256.9179	8.0119	**0.000**
Center-Left	-14.1749	13.2252	299.9847	-1.0718	0.285
Center	-20.3928	10.9209	309.9094	-1.8673	**0.063**
Center- Right	-20.5174	9.8497	301.4696	-2.0830	**0.038**
Right	7.2931	10.4370	304.5310	0.6988	0.485
Far´right	-23.3044	10.4884	303.4576	-2.2219	**0.027**

ICC		
ICC_adjusted	ICC_unadjusted	optional
0.0233	0.0231	FALSE

Marginal and Conditional R^2		
R2_conditional	R2_marginal	optional
0.0327	0.0096	FALSE

EMMs Governor's Ideology (Tukey)					
contrast	estimate	SE	df	t.ratio	p.value
Center - (Center-Right)	14.1749	13.2273	1383552.1099	1.0716	0.893
Center - (Center-Left)	20.3928	10.9278	412902.3934	1.8661	0.423
Center - Right	20.5174	9.8520	1237436.9317	2.0826	0.296
Center - Left	-7.2931	10.4410	837956.1572	-0.6985	0.982
Center - (Far-Right)	23.3044	10.4917	937330.3137	2.2212	0.228
(Center-Right) - (Center-Left)	6.2179	10.2563	604145.5730	0.6063	0.991
(Center-Right) - Right	6.3425	9.4642	1377989.0148	0.6702	0.985
(Center-Right) - Left	-21.4681	9.9452	1087546.2951	-2.1586	0.257
(Center-Right) - (Far-Right)	9.1295	9.7773	1285381.6881	0.9337	0.938
(Center-Left) - Right	0.1246	4.4384	42917.5599	0.0281	1.000
(Center-Left) - Left	-27.6860	4.5802	71641.2031	-6.0447	**0.000**
(Center-Left) - (Far-Right)	2.9116	4.4686	88740.6138	0.6516	0.987
Right - Left	-27.8105	3.4600	155747.1739	-8.0377	**0.000**
Right-wing (Far-right)	2.7870	3.6376	240596.7476	0.7662	0.973
Left - (Far-Right)	30.5976	4.0645	248711.2984	7.5279	**0.000**

Figure S42

Estimated Marginal Means of Governors' Ideology on Indigenous School Language Practices (State Level)

Indigenous Language

Model Coefficients					
Ideology	Estimate	Std. Error	df	t value	Pr(>|t|)
Left	8.4763	6.1683	112.8672	1.3742	0.172
Center-Left	1.9572	6.3985	298.0079	0.3059	0.760
Center	7.9240	5.3223	301.5112	1.4888	0.138
Center- Right	5.4726	4.7697	298.4175	1.1474	0.252
Right	7.8081	5.0646	299.4305	1.5417	0.124
Far´right	1.8634	5.0858	299.0970	0.3664	0.714

ICC		
ICC_adjusted	ICC_unadjusted	optional
0.0813	0.0811	FALSE

Marginal and Conditional R^2		
R2_conditional	R2_marginal	optional
0.0826	0.0014	FALSE

EMMs Governor's Ideology (Tukey)					
contrast	estimate	SE	df	t.ratio	p.value
Center - (Center-Right)	-1.9572	6.3994	1401001.0529	-0.3058	1.000
Center - (Center-Left)	-7.9240	5.3247	680483.0036	-1.4882	0.672
Center - Right	-5.4726	4.7705	1339034.6378	-1.1472	0.862
Center - Left	-7.8081	5.0660	1080009.5097	-1.5413	0.637
Center - (Far-Right)	-1.8634	5.0870	1083459.9992	-0.3663	0.999
(Center-Right) - (Center-Left)	-5.9668	4.9903	909053.7982	-1.1957	0.839
(Center-Right) - Right	-3.5154	4.5808	1408927.9225	-0.7674	0.973
(Center-Right) - Left	-5.8509	4.8221	1285195.1133	-1.2133	0.831
(Center-Right) - (Far-Right)	0.0938	4.7354	1355721.8257	0.0198	1.000
(Center-Left) - Right	2.4514	2.2099	100369.7205	1.1093	0.878
(Center-Left) - Left	0.1159	2.2640	165346.7681	0.0512	1.000
(Center-Left) - (Far-Right)	6.0606	2.2043	203230.6353	2.7494	0.066
Right - Left	-2.3355	1.6959	328519.9561	-1.3772	0.741
Right-wing (Far-right)	3.6092	1.7788	412321.5144	2.0290	0.326
Left - (Far-Right)	5.9447	1.9863	501828.6550	2.9929	**0.033**

Figure S43

Estimated Marginal Means of Governors' Ideology on Indigenous School Language Practices (State Level)

Portuguese Language

Model Coefficients					
Ideology	Estimate	Std. Error	df	t value	Pr(>|t|)
Left	32.1411	10.1036	100.9548	3.1811	**0.002**
Center-Left	1.4334	10.2530	297.0291	0.1398	0.889
Center	15.8228	8.5310	300.3295	1.8547	0.065
Center- Right	12.1130	7.6432	297.4108	1.5848	0.114
Right	11.1661	8.1165	298.3627	1.3757	0.170
Far´right	-2.3202	8.1503	298.0503	-0.2847	0.776

ICC		
ICC_adjusted	ICC_unadjusted	optional
0.0897	0.0890	FALSE

Marginal and Conditional R^2		
R2_conditional	R2_marginal	optional
0.0961	0.0071	FALSE

EMMs Governor's Ideology (Tukey)					
contrast	estimate	SE	df	t.ratio	p.value
Center - (Center-Right)	-1.4334	10.2544	1402166.5585	-0.1398	1.000
Center - (Center-Left)	-15.8228	8.5348	718198.7189	-1.8539	0.431
Center - Right	-12.1130	7.6446	1346403.8449	-1.5845	0.609
Center - Left	-11.1661	8.1187	1103080.4260	-1.3754	0.742
Center - (Far-Right)	2.3202	8.1522	1096040.9328	0.2846	1.000
(Center-Right) - (Center-Left)	-14.3894	7.9983	948918.2522	-1.7991	0.466
(Center-Right) - Right	-10.6796	7.3404	1411011.5522	-1.4549	0.693
(Center-Right) - Left	-9.7326	7.7277	1302618.5496	-1.2595	0.807
(Center-Right) - (Far-Right)	3.7536	7.5884	1361152.6715	0.4946	0.996
(Center-Left) - Right	3.7098	3.5454	111613.7546	1.0464	0.902
(Center-Left) - Left	4.6567	3.6310	183402.2898	1.2825	0.795
(Center-Left) - (Far-Right)	18.1429	3.5350	225331.6048	5.1323	**0.000**
Right - Left	0.9470	2.7189	356794.0107	0.3483	0.999
Right-wing (Far-right)	14.4332	2.8516	434477.9787	5.0614	**0.000**
Left - (Far-Right)	13.4862	3.1842	540762.3160	4.2354	**0.000**

Estimated Marginal Means of Governors' Ideology on Indigenous School Language Practices (State Level)

Indigenous and Portuguese Language

Model Coefficients					
Ideology	Estimate	Std. Error	df	t value	Pr(>|t|)
Left	59.7936	11.4508	131.9478	5.2218	**0.000**
Center-Left	-3.3997	12.4109	297.4248	-0.2739	0.784
Center	-23.8462	10.3151	301.9139	-2.3118	**0.021**
Center- Right	-17.6945	9.2507	297.9638	-1.9128	**0.057**
Right	-19.1919	9.8204	299.2697	-1.9543	**0.052**
Far´right	0.3867	9.8624	298.8361	0.0392	0.969

ICC		
ICC_adjusted	ICC_unadjusted	optional
0.0652	0.0647	FALSE

Marginal and Conditional R^2		
R2_conditional	R2_marginal	optional
0.0726	0.0079	FALSE

EMMs Governor's Ideology (Tukey)					
contrast	estimate	SE	df	t.ratio	p.value
Center - (Center-Right)	3.3997	12.4126	1398088.9824	0.2739	1.000
Center - (Center-Left)	23.8462	10.3203	605139.6928	2.3106	0.190
Center - Right	17.6945	9.2524	1321319.3624	1.9124	0.394
Center - Left	19.1919	9.8234	1028212.7105	1.9537	0.369
Center - (Far-Right)	-0.3867	9.8649	1054313.5029	-0.0392	1.000
(Center-Right) - (Center-Left)	20.4465	9.6735	827004.5448	2.1137	0.280
(Center-Right) - Right	14.2948	8.8848	1403780.5367	1.6089	0.593
(Center-Right) - Left	15.7922	9.3511	1245309.4952	1.6888	0.539
(Center-Right) - (Far-Right)	-3.7864	9.1841	1342722.5610	-0.4123	0.998
(Center-Left) - Right	-6.1517	4.2734	80880.5784	-1.4395	0.703
(Center-Left) - Left	-4.6543	4.3813	133884.2388	-1.0623	0.896
(Center-Left) - (Far-Right)	-24.2329	4.2668	164683.6151	-5.6793	**0.000**
Right - Left	1.4974	3.2849	275977.6546	0.4558	0.998
Right-wing (Far-right)	-18.0812	3.4464	367176.0076	-5.2465	**0.000**
Left - (Far-Right)	-19.5786	3.8486	427973.6623	-5.0872	**0.000**

Figure S48

Estimated Marginal Means of Mayor's and Governor's Ideology on Indigenous School Language Practices (Municipal Level)

Indigenous Material

Model Coefficients						
Level of government	Ideology	Estimate	Std. Error	df	t value	Pr(>|t|)
Mayor	Left	65.2748	13.1456	966.7027	4.9655	**0.000**
Mayor	Center-Left	-17.9520	8.6591	2449.7091	-2.0732	**0.038**
Mayor	Center	-21.7785	8.0301	2448.7565	-2.7121	**0.007**
Mayor	Center- Right	-17.1766	7.5410	2451.0406	-2.2778	**0.023**
Mayor	Right	11.5920	7.6843	2451.3672	1.5085	0.132
Mayor	Far´right	-18.0533	7.7782	2451.9135	-2.3210	**0.020**
Governor	Left	-5.2712	11.1655	2454.1063	-0.4721	0.637
Governor	Center-Left	-8.3617	11.0406	2438.8654	-0.7574	0.449
Governor	Center	-8.7992	10.4513	2452.8643	-0.8419	0.400
Governor	Center- Right	-9.4286	11.3102	2323.5791	-0.8336	0.405
Governor	Right	-14.5993	11.1249	2445.1041	-1.3123	0.190
Governor	Far´right	0.4065	0.3318	2379.9989	1.2251	0.221

ICC (Intraclass Correlation)		
ICC_adjusted	ICC_unadjusted	optional
0.01866	0.01853	FALSE

Marginal and Conditional R^2		
R2_conditional	R2_marginal	optional
0.02571	0.00718	FALSE

EMMs Mayor's Ideology (Tukey)					
contrast	estimate	SE	df	t.ratio	p.value
Center - (Center-Right)	17.9520	8.6620	53601.0426	2.0725	0.302
Center - (Center-Left)	21.7785	8.0327	49946.1676	2.7112	0.073
Center - Right	17.1766	7.5436	44602.9281	2.2770	0.203
Center - Left	-11.5920	7.6868	47203.8904	-1.5080	0.659
Center - (Far-Right)	18.0533	7.7809	47526.3273	2.3202	0.186
(Center-Right) - (Center-Left)	3.8266	5.2939	126701.5437	0.7228	0.979
(Center-Right) - Right	-0.7754	4.4888	122090.6256	-0.1727	1.000
(Center-Right) - Left	-29.5439	4.6556	147666.7617	-6.3458	**0.000**
(Center-Right) - (Far-Right)	0.1013	4.9900	109689.1479	0.0203	1.000
(Center-Left) - Right	-4.6019	3.2212	151103.5525	-1.4287	0.710
(Center-Left) - Left	-33.3705	3.4850	196102.5564	-9.5755	**0.000**
(Center-Left) - (Far-Right)	-3.7252	3.8288	129796.5445	-0.9729	0.927
Right - Left	-28.7686	2.0218	514077.7880	-14.2292	**0.000**
Right-wing (Far-right)	0.8767	2.6415	104848.7190	0.3319	0.999
Left - (Far-Right)	29.6453	2.9999	162461.9650	9.8820	**0.000**

EMMs Governor's Ideology (Tukey)					
contrast	estimate	SE	df	t.ratio	p.value
Center - (Center-Right)	5.2712	11.1750	43332.2696	0.4717	0.997
Center - (Center-Left)	8.3617	11.0521	35227.8502	0.7566	0.975
Center - Right	8.7992	10.4607	34969.6157	0.8412	0.960
Center - Left	9.4286	11.3426	17582.2763	0.8313	0.962
Center - (Far-Right)	14.5993	11.1367	34716.4005	1.3109	0.779
(Center-Right) - (Center-Left)	3.0905	5.3572	200406.5047	0.5769	0.993
(Center-Right) - Right	3.5280	4.4633	683140.0354	0.7905	0.969
(Center-Right) - Left	4.1574	6.2847	7848.8002	0.6615	0.986
(Center-Right) - (Far-Right)	9.3281	5.5713	120240.2870	1.6743	0.549
(Center-Left) - Right	0.4375	3.0988	46020.6264	0.1412	1.000
(Center-Left) - Left	1.0669	5.4485	2670.7980	0.1958	1.000
(Center-Left) - (Far-Right)	6.2376	3.1044	91197.4891	2.0093	0.337
Right - Left	0.6294	4.7367	1960.9303	0.1329	1.000
Right-wing (Far-right)	5.8000	3.1348	29071.4391	1.8502	0.433
Left - (Far-Right)	5.1707	5.5508	2182.4254	0.9315	0.938

Figure S49

Estimated Marginal Means of Mayor's and Governor's Ideology on Indigenous School Language Practices (Municipal Level)

Indigenous Language

Model Coefficients						
Level of government	Ideology	Estimate	Std. Error	df	t value	Pr(>|t|)
Mayor	Left	6.3551	6.7389	195.2114	0.9430	0.347
Mayor	Center-Left	1.6900	3.9869	2438.5714	0.4239	0.672
Mayor	Center	-0.9408	3.6966	2437.3312	-0.2545	0.799
Mayor	Center- Right	1.6935	3.4727	2439.3441	0.4877	0.626
Mayor	Right	5.5549	3.5388	2439.5494	1.5697	0.117
Mayor	Far´right	3.1850	3.5826	2440.4737	0.8890	0.374
Governor	Left	-0.6333	5.1604	2455.4755	-0.1227	0.902
Governor	Center-Left	-0.4291	5.1128	2455.6153	-0.0839	0.933
Governor	Center	4.1350	4.8322	2455.8588	0.8557	0.392
Governor	Center- Right	4.3510	5.2634	2443.3306	0.8267	0.409
Governor	Right	-1.2276	5.1482	2455.9993	-0.2385	0.812
Governor	Far´right	-0.1797	0.1540	2455.3658	-1.1674	0.243

ICC (Intraclass Correlation)		
ICC_adjusted	ICC_unadjusted	optional
0.0702	0.0699	FALSE

Marginal and Conditional R^2		
R2_conditional	R2_marginal	optional
0.0732	0.0033	FALSE

EMMs Mayor's Ideology (Tukey)					
contrast	estimate	SE	df	t.ratio	p.value
Center - (Center-Right)	-1.6900	3.9877	53826.3209	-0.4238	0.998
Center - (Center-Left)	0.9408	3.6973	49922.5735	0.2545	1.000
Center - Right	-1.6935	3.4734	44636.1316	-0.4876	0.997
Center - Left	-5.5549	3.5396	47233.2840	-1.5694	0.619
Center - (Far-Right)	-3.1850	3.5833	47608.3465	-0.8888	0.949
(Center-Right) - (Center-Left)	2.6308	2.4384	131237.6286	1.0789	0.890
(Center-Right) - Right	-0.0035	2.0689	125284.5581	-0.0017	1.000
(Center-Right) - Left	-3.8649	2.1461	152429.4869	-1.8009	0.465
(Center-Right) - (Far-Right)	-1.4950	2.3011	115030.1583	-0.6497	0.987
(Center-Left) - Right	-2.6343	1.4836	158671.5942	-1.7756	0.482
(Center-Left) - Left	-6.4957	1.6052	199498.5583	-4.0467	**0.001**
(Center-Left) - (Far-Right)	-4.1258	1.7635	130618.6067	-2.3396	0.178
Right - Left	-3.8614	0.9310	532342.8794	-4.1477	**0.000**
Right-wing (Far-right)	-1.4915	1.2174	110194.4643	-1.2251	0.825
Left - (Far-Right)	2.3699	1.3826	166561.4104	1.7141	0.522

EMMs Governor's Ideology (Tukey)					
contrast	estimate	SE	df	t.ratio	p.value
Center - (Center-Right)	0.6333	5.1644	49212.1124	0.1226	1.000
Center - (Center-Left)	0.4291	5.1175	41512.5762	0.0838	1.000
Center - Right	-4.1350	4.8363	39435.7636	-0.8550	0.957
Center - Left	-4.3510	5.2715	32600.5209	-0.8254	0.963
Center - (Far-Right)	1.2276	5.1527	40918.5206	0.2382	1.000
(Center-Right) - (Center-Left)	-0.2042	2.4760	286472.8318	-0.0825	1.000
(Center-Right) - Right	-4.7683	2.0540	697739.3033	-2.3214	0.185
(Center-Right) - Left	-4.9843	2.9385	43712.7582	-1.6962	0.534
(Center-Right) - (Far-Right)	0.5944	2.5726	193457.4150	0.2310	1.000
(Center-Left) - Right	-4.5641	1.4429	120182.0055	-3.1632	**0.019**
(Center-Left) - Left	-4.7801	2.5912	14196.7116	-1.8447	0.437
(Center-Left) - (Far-Right)	0.7985	1.4392	236451.6071	0.5549	0.994
Right - Left	-0.2160	2.2488	12711.8589	-0.0961	1.000
Right-wing (Far-right)	5.3626	1.4548	77818.3108	3.6862	**0.003**
Left - (Far-Right)	5.5786	2.6345	13188.4401	2.1175	0.278

Figure S50

Estimated Marginal Means of Mayor's and Governor's Ideology on Indigenous School Language Practices (Municipal Level)

Portuguese Language

Model Coefficients						
Level of government	Ideology	Estimate	Std. Error	df	t value	Pr(>|t|)
Mayor	Left	41.0954	13.2804	220.5069	3.0944	**0.002**
Mayor	Center-Left	22.4925	7.9126	2439.9104	2.8426	**0.005**
Mayor	Center	11.9622	7.3367	2438.7278	1.6305	0.103
Mayor	Center- Right	11.5806	6.8921	2440.6927	1.6803	**0.093**
Mayor	Right	-13.8347	7.0234	2440.8851	-1.9698	**0.049**
Mayor	Far´right	13.1852	7.1102	2441.7763	1.8544	**0.064**
Governor	Left	-13.7125	10.2403	2455.6667	-1.3391	0.181
Governor	Center-Left	-1.6867	10.1452	2455.3870	-0.1663	0.868
Governor	Center	-7.8250	9.5889	2455.9472	-0.8161	0.415
Governor	Center- Right	8.6497	10.4427	2441.7158	0.8283	0.408
Governor	Right	9.0393	10.2156	2455.9641	0.8849	0.376
Governor	Far´right	-2.1709	0.3055	2454.9194	-7.1058	**0.000**

ICC (Intraclass Correlation)		
ICC_adjusted	ICC_unadjusted	optional
0.0664	0.0657	FALSE

Marginal and Conditional R^2		
R2_conditional	R2_marginal	optional
0.0763	0.0106	FALSE

EMMs Mayor's Ideology (Tukey)					
contrast	estimate	SE	df	t.ratio	p.value
Center - (Center-Right)	-22.4925	7.9143	53820.7801	-2.8420	**0.051**
Center - (Center-Left)	-11.9622	7.3381	49924.7641	-1.6302	0.578
Center - Right	-11.5806	6.8936	44636.3139	-1.6799	0.545
Center - Left	13.8347	7.0248	47233.2261	1.9694	0.360
Center - (Far-Right)	-13.1852	7.1117	47606.5440	-1.8540	0.431
(Center-Right) - (Center-Left)	10.5302	4.8393	131068.3667	2.1760	0.249
(Center-Right) - Right	10.9119	4.1059	125112.8613	2.6576	**0.084**
(Center-Right) - Left	36.3272	4.2591	152185.3925	8.5293	**0.000**
(Center-Right) - (Far-Right)	9.3073	4.5667	114796.6467	2.0381	0.321
(Center-Left) - Right	0.3817	2.9444	158517.7547	0.1296	1.000
(Center-Left) - Left	25.7970	3.1857	199404.1842	8.0977	**0.000**
(Center-Left) - (Far-Right)	-1.2229	3.4999	130576.0259	-0.3494	0.999
Right - Left	25.4153	1.8477	531839.0274	13.7553	**0.000**
Right-wing (Far-right)	-1.6046	2.4161	110075.4165	-0.6641	0.986
Left - (Far-Right)	-27.0199	2.7439	166427.9557	-9.8474	**0.000**

EMMs Governor's Ideology (Tukey)					
contrast	estimate	SE	df	t.ratio	p.value
Center - (Center-Right)	13.7125	10.2482	48830.7551	1.3380	0.764
Center - (Center-Left)	1.6867	10.1545	41118.5286	0.1661	1.000
Center - Right	7.8250	9.5971	39137.0608	0.8154	0.965
Center - Left	-8.6497	10.4591	31803.1291	-0.8270	0.963
Center - (Far-Right)	-9.0393	10.2247	40557.9311	-0.8841	0.950
(Center-Right) - (Center-Left)	-12.0258	4.9136	282827.8988	-2.4474	0.140
(Center-Right) - Right	-5.8875	4.0766	697523.7212	-1.4442	0.700
(Center-Right) - Left	-22.3622	5.8297	40609.8432	-3.8359	**0.002**
(Center-Right) - (Far-Right)	-22.7518	5.1054	191017.8923	-4.4564	**0.000**
(Center-Left) - Right	6.1383	2.8628	115879.8919	2.1442	0.265
(Center-Left) - Left	-10.3364	5.1386	13134.2013	-2.0115	0.336
(Center-Left) - (Far-Right)	-10.7260	2.8558	228244.8089	-3.7559	**0.002**
Right - Left	-16.4747	4.4597	11692.9288	-3.6941	**0.003**
Right-wing (Far-right)	-16.8643	2.8867	75561.1634	-5.8422	**0.000**
Left - (Far-Right)	-0.3896	5.2247	12146.3184	-0.0746	1.000

Figure S51

Estimated Marginal Means of Mayor’s and Governor’s Ideology on Indigenous School Language Practices (Municipal Level)

Indigenous and Portuguese Language

Model Coefficients						
Level of government	Ideology	Estimate	Std. Error	df	t value	Pr(>|t|)
Mayor	Left	53.0116	13.7740	266.5496	3.8487	**0.000**
Mayor	Center-Left	-24.1890	8.3683	2440.7495	-2.8905	**0.004**
Mayor	Center	-11.0384	7.7594	2439.5228	-1.4226	0.155
Mayor	Center- Right	-13.3020	7.2890	2441.7124	-1.8249	**0.068**
Mayor	Right	8.2554	7.4278	2441.9063	1.1114	0.266
Mayor	Far´right	-16.4204	7.5194	2442.8601	-2.1837	**0.029**
Governor	Left	14.1886	10.8251	2455.9543	1.3107	0.190
Governor	Center-Left	1.9585	10.7227	2454.2269	0.1826	0.855
Governor	Center	3.5433	10.1361	2455.9734	0.3496	0.727
Governor	Center- Right	-13.2958	11.0326	2432.8345	-1.2051	0.228
Governor	Right	-7.9441	10.7979	2455.5308	-0.7357	0.462
Governor	Far´right	2.3478	0.3229	2452.3746	7.2714	**0.000**

ICC (Intraclass Correlation)		
ICC_adjusted	ICC_unadjusted	optional
0.0563	0.0558	FALSE

Marginal and Conditional R^2		
R2_conditional	R2_marginal	optional
0.0648	0.0090	FALSE

EMMs Mayor's Ideology (Tukey)					
contrast	estimate	SE	df	t.ratio	p.value
Center - (Center-Right)	24.1890	8.3702	53802.5681	2.8899	**0.045**
Center - (Center-Left)	11.0384	7.7609	49931.5043	1.4223	0.713
Center - Right	13.3020	7.2906	44636.5565	1.8245	0.450
Center - Left	-8.2554	7.4294	47232.9626	-1.1112	0.877
Center - (Far-Right)	16.4204	7.5211	47600.7051	2.1832	0.245
(Center-Right) - (Center-Left)	-13.1506	5.1178	130543.2948	-2.5696	0.105
(Center-Right) - Right	-10.8870	4.3420	124604.2464	-2.5074	0.122
(Center-Right) - Left	-32.4444	4.5039	151459.2422	-7.2036	**0.000**
(Center-Right) - (Far-Right)	-7.7686	4.8289	114089.5320	-1.6088	0.593
(Center-Left) - Right	2.2636	3.1139	157988.6921	0.7269	0.979
(Center-Left) - Left	-19.2938	3.3691	199097.9482	-5.7267	**0.000**
(Center-Left) - (Far-Right)	5.3820	3.7013	130449.0393	1.4541	0.694
Right - Left	-21.5574	1.9541	530227.4359	-11.0321	**0.000**
Right-wing (Far-right)	3.1184	2.5550	109673.3786	1.2205	0.827
Left - (Far-Right)	24.6758	2.9016	166006.2668	8.5042	**0.000**

EMMs Governor's Ideology (Tukey)					
contrast	estimate	SE	df	t.ratio	p.value
Center - (Center-Right)	-14.1886	10.8337	47738.3990	-1.3097	0.780
Center - (Center-Left)	-1.9585	10.7328	39989.7784	-0.1825	1.000
Center - Right	-3.5433	10.1449	38282.0161	-0.3493	0.999
Center - Left	13.2958	11.0514	29468.0660	1.2031	0.836
Center - (Far-Right)	7.9441	10.8079	39504.9420	0.7350	0.978
(Center-Right) - (Center-Left)	12.2301	5.1948	271711.2602	2.3543	0.173
(Center-Right) - Right	10.6452	4.3116	696736.1985	2.4690	0.133
(Center-Right) - Left	27.4844	6.1577	32441.3345	4.4634	**0.000**
(Center-Right) - (Far-Right)	22.1326	5.3982	183036.7448	4.1000	**0.001**
(Center-Left) - Right	-1.5848	3.0246	103642.0209	-0.5240	0.995
(Center-Left) - Left	15.2543	5.4201	10413.0711	2.8144	**0.055**
(Center-Left) - (Far-Right)	9.9026	3.0186	204093.0411	3.2805	**0.013**
Right - Left	16.8391	4.7049	9094.3405	3.5790	**0.005**
Right-wing (Far-right)	11.4874	3.0509	68582.7215	3.7652	**0.002**
Left - (Far-Right)	-5.3517	5.5120	9490.1224	-0.9709	0.927
